# Computational analysis and validation of UGT1A1/4 missense variants impacting tecovirimat metabolism in monkeypox patients

**DOI:** 10.3389/fsysb.2026.1821230

**Published:** 2026-06-11

**Authors:** Amro A. Abdelazim, Sameh E. Hassanein, Mohamad Maged, Nada S. Al-Theyab, Itoh Kimiko, Kotb A. Attia

**Affiliations:** 1 International Dryland Development Commission (IDDC), Cairo, Egypt; 2 Bioinformatics Program, School of Biotechnology, Nile University, Giza, Egypt; 3 Applied Biotechnology Program, School of Biotechnology, Nile University, Giza, Egypt; 4 Department of Pharmaceutical Chemistry, College of Pharmacy, King Saud University, Riyadh, Saudi Arabia; 5 Institute of Science and Technology, Niigata University, Niigata, Japan; 6 Center of Excellence in Biotechnology Research, King Saud University, Riyadh, Saudi Arabia

**Keywords:** in-silico, missense variant, monkeypox, pharmacogenetics, single nucleotide polymorphisms (SNPs), tecovirimat

## Abstract

**Introduction:**

Single nucleotide polymorphisms (SNPs) in genes encoding drug-metabolizing enzymes can significantly impact a patient’s response to medication. Uridine diphosphate glucuronosyltransferase 1 family A1 and A4 (UGT1A1/4) are crucial enzymes for metabolizing tecovirimat, the first oral antiviral drug approved for treating the monkeypox virus.

**Methods:**

This study used a comprehensive in-silico workflow to assess the deleterious effects of 842 missense mutations and 308 SNPs in the non-coding regions of the UGT1A1 gene, alongside 700 missense mutations and 324 SNPs in the non-coding regions of the UGT1A4 gene. An ensemble of in-silico prediction, structural modelling, and docking tools was employed.

**Results:**

We identified six missense variants that may compromise the structural integrity and function of the UGT1A1/4 enzymes. Specifically, the UGT1A1 SNPs G308R, P356T and G374S, while in UGT1A4 the SNPs G309R, P357T and G375S, were predicted to be the most harmful missense SNPs.

**Discussion:**

These mutations could affect drug-enzyme binding, potentially altering tecovirimat’s therapeutic efficacy.

## 1 Introduction

Human monkeypox (Mpox) is a serious zoonotic infection caused by the monkeypox virus (MPXV) (Huang et al., 2022; Alakunle et al., 2024). The MPXV is a double-stranded DNA virus (dsDNA) classified under the Poxviridae family and the Orthopoxvirus genus, including two distinct clades: West African and Congo Basin (Karagoz et al., 2023). Mpox is an endemic infectious disease in West Africa and parts of central Africa. Only a few cases were reported outside Africa prior to the 2022 outbreak, all of which were linked to imports from endemic regions (Huang et al., 2022). Tecovirimat is a novel drug approved for use in treating Mpox infections in the United States, Canada, and Europe (Gessain et al., 2022; Almehmadi et al., 2022). After the Mpox epidemic in 2022, the Food and Drug Administration (FDA) and European Medicines Agency (EMA) issued emergency authorization for tecovirimat as an oral treatment for Mpox, indicating its potential clinical effectiveness and marked safety profile (McQuiston et al., 2023). Few studies addressed the pharmacokinetics of tecovirimat (Tempestilli et al., 2024), however, there was a notable scarcity of research on the drug’s pharmacogenetics.

The *UGT1A1/4* genes, which encode the enzymes uridine diphosphate glucuronosyl transferase 1A1/4 (UGT1A1/4), are located on human chromosome 2 (2q37.1) in the same locus within the *UGT1A* gene complex. The primary transcript contains nine functional first exons that are independently spliced to four exons, resulting in nine mature UGT1A transcripts with distinct 5′ ends and identical 3′ ends (National Center for Biotechnology Information, 2025a; National Center for Biotechnology Information, 2025b; National Center for Biotechnology Information, 2024). The enzymes are primarily found in the liver and intestines (Hulshof et al., 2023). UGT1A1/4 are major phase II metabolizing enzymes that facilitate the metabolism and detoxification of medications and various endogenous compounds (Topić et al., 2024). These enzymes are primarily responsible for the biotransformation of tecovirimat (Tempestilli et al., 2024; DeLaurentis et al., 2022; Hoy, 2018), *In vitro* data demonstrates that the metabolism of tecovirimat proceeds primarily through conjugation by multiple human uridine diphosphate glucuronosyltransferases (UGTs), with negligible contribution from major CYP P450 enzymes (Grosenbach et al., 2018). Single nucleotide polymorphisms (SNPs) in both coding or non-coding regions of the genes encoding the UGT1A1/4 enzymes can alter the proteins' structure and function. Polymorphic variations within the *UGT1A1* gene can impact its metabolic capacity, leading to altered pharmacokinetics and contributing to the wide interpatient differences observed in the efficacy and toxicity of its substrate drugs (Khatri et al., 2021; Basit et al., 2020). Also, mutated variants of *UGT1A4* can be associated with changes in its metabolic capacity, potentially impacting the disposition of drugs like tecovirimat that are metabolized by this enzyme (Basit et al., 2020; Howlader et al., 2022). Some SNPs are synonymous that do not change the translated protein (Samad et al., 2023), while others are missense SNPs that alter the translated amino acid sequence, hence modifying the shape and/or activity of the resultant protein (Ahammad et al., 2023). However, not all missense SNPs are damaging or deleterious, and it is crucial to identify and differentiate the deleterious from the non-deleterious SNPs (Ahammad et al., 2023).

Limited information exists on the missense SNPs in the gene encoding the UGT1A1/4 drug-metabolizing enzymes. This arises partly from the extensive genetic variants within the human genome and the poor mapping of genetic polymorphisms in some regions of the world. Investigating these polymorphisms is beneficial for providing individualized pharmacotherapeutic decisions.

A multitude of *in silico* algorithms has been used to assess the potential therapeutic outcomes prior to the initiation of clinical trials to guide the clinical investigation (Singh et al., 2022). Advances in bioinformatics and *in silico* technology could help identify and assess the deleteriousness of SNPs (Reza et al., 2021; Abdelazim et al., 2025). Additionally, bioinformatics could be useful when discovery of new drugs or repurposing of existing compounds is needed (Sedky et al., 2024). The accuracy of predicting the biological impact of mutations is enhanced when multiple algorithms are used in conjunction rather than relying on a single tool (Zhao et al., 2023). Furthermore, the accuracy of bioinformatics research is significantly reliant on both the quality of the input data and its volume. Unreliable or insufficient data may result in misleading conclusions and present a notable difficulty in biomedical research. This becomes significant when the outcomes might affect the therapeutic decisions and patient’s healthcare (Kumuthini et al., 2020). Therefore, it is essential to validate all used computational techniques and *in silico* tools to enhance their real-world applicability (Rhee, 2005).

This study examined the potential effects of missense SNPs and mutations in the non-coding regions of the genes encoding the UGT1A1/4 enzymes on their structure and function. Additionally, we differentiated the deleterious SNPs from the benign ones. Also, we performed SNP modelling and molecular docking simulations to predict and analyze enzyme-tecovirimat interactions.

## 2 Methods

### 2.1 Data retrieval and deleteriousness assessment of UGT1A1 SNPs

The ENSEMBL database (Martin et al., 2023) was used to retrieve UGT1A1/4 SNPs data. For the missense variants, filtration was done according to the search parameters; “*homo sapiens*,” “missense variations,” and “coding sequence variants.” While for the non-coding region variants, filtration was done according to the search parameters; “*homo sapiens*,” “5 prime UTR variant”, and “3 prime UTR variant”.

**FIGURE 1:**
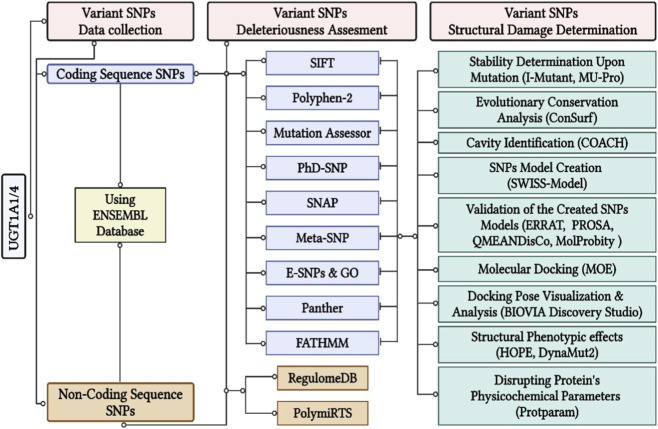
The whole pipeline of the *in silico* methodology illustrated step-by-step including the tools/programs/websites and databases utilized.

The wild-type (WT) amino acid sequences of the enzymes were retrieved from the UniProt database using the ID: “P22309” for UGT1A1 and ID: “P22310” for UGT1A4. All bioinformatics tools used in this study are listed in (Supplementary Table S12). While (Figure 1) illustrates the pipeline of the *in silico* methodology used in this work.

Nine *in silico* tools were used to assess the deleteriousness of UGT1A1/4 variants: SIFT (SIFT4G predictions) with the outcomes (Deleterious or Tolerated) (Sim et al., 2012), PolyPhen-2 (Polymorphism Phenotyping v2) with the outcomes (Probably damaging, Possibly damaging or Benign) (Adzhubei et al., 2013), Mutation Assessor with the outcomes (High, Medium, Low or Neutral) (Reva et al., 2011), PhD-SNP with outcomes (Disease or Neutral) (Capriotti and Fariselli, 2017), SNAP with outcomes (Disease or Neutral) (Bromberg and Rost, 2007), Meta-SNP with outcomes (Disease or Neutral) (Capriotti et al., 2013), E-SNPs&GO with outcomes (Pathogenic or Benign) (Manfredi et al., 2022), PANTHER v19.0 with outcomes (Disease or Neutral) (Tang and Thomas, 2016) and FATHMM v2.3 with outcomes (Damaging or Tolerated) (Shihab et al., 2013). Each SNP was assigned a maximum score of 9 based on the number of tools that indicated deleterious outcomes (Damaging, Deleterious, Disease) as opposed to non-deleterious results (Tolerated, Benign, Neutral). Each tool that returned a “deleterious result” added “+1” to the score of the SNP, whereas each “non-deleterious result” contributed to a “0 score” for the SNP. To generate a high-confidence set of candidate variants for subsequent analysis, we adopted a highly stringent consensus approach. A variant was classified as deleterious only if it was predicted as damaging by all nine *in silico* tools (a score of 9/9). This conservative strategy was chosen to prioritize specificity and minimize the inclusion of false-positive predictions, with the acknowledged trade-off of lower sensitivity (i.e., a higher rate of false negatives).

### 2.2 Stability determination of UGT1A1/4 SNPs upon mutation and evolutionary conservation analysis

Two *in silico* tools, I-Mutant (Capriotti et al., 2006) and MuPro (Cheng et al., 2006), were used to assess the stability of UGT1A1/4 mutant enzymes. Only the SNPs that caused decrease in stability by both tools were selected for further investigations.

The Consurf tool (Ben Chorin et al., 2020) was used to assess protein functional and structural regions (Pavithran and Kumavath, 2021) and performed high-throughput characterization. The evolutionary conservation of amino acid residues was assessed using a numerical scoring system ranging from 1 to 9. Residues were classified into three categories based on their scores: variable (scores 1–3), moderately conserved (scores 4–6), and highly conserved (scores 7–9).

### 2.3 Modelling and model validation of SNPs

Modelling of protein structure is crucial in biomedical research. Homology modelling was done using the “Swiss-Model web service” (Waterhouse et al., 2018) for the UGT1A1/4 wild-type enzymes as well as their respective SNPs.

To enhance the quality and accuracy of the homology modelling output, robust validation was required (Haddad et al., 2020). ERRAT (Colovos and Yeates, 1993), PROSA (Wiederstein and Sippl, 2007), QMEANDisCo (Studer et al., 2020) (Benkert et al., 2011) and MolProbity (Chen et al., 2010) were used to validate the structural models prior to their application in the docking step. The ERRAT score indicated the statistics of non-bonded interactions among various atom types based on distinctive atomic interactions. A value of 95% or higher was the benchmark for high-resolution structures, indicating that the mutant structures examined met the ERRAT standard values (Colovos and Yeates, 1993). The PROSA z-score assessed the overall quality of the model by evaluating total energy deviation based on energy distribution obtained from random conformations and by comparing the model with reference structures of equivalent size from the PDB database. Models with z scores of 4.0 or lower were classified as low-quality models and excluded (Wiederstein and Sippl, 2007). QMEANDisCo global score approaching 1.0. Additionally, models were required to have a high Global Model Quality Estimate (GMQE) score. Finally, MolProbity geometric quality assessment required that over 98% of residues be in Ramachandran favored regions, with Ramachandran outliers near 0%, alongside low overall MolProbity scores and clash scores. Models failing to meet these standards were excluded from further analysis.

### 2.4 UGT1A1/4 protein’s binding cavity prediction

The ligand-binding cavities of the UGT1A1/4 proteins were predicted using the COACH tool with its default parameters (Yang et al., 2013). The analysis was performed on the three-dimensional protein models previously generated by I-TASSER. The highest-ranking predicted binding site was then used for analysis of SNPs that might occur within. Also, the predicted binding cavities were utilized during docking experiments.

### 2.5 Molecular docking analysis and visualization of the docking results

To ensure transparency and reproducibility, the docking workflow was executed in the following stepwise procedure using the Molecular Operating Environment (MOE 2014.0901 i4w9) software (I. Chemical Computing Group and Molecular Operating Environment MOE, 2014).

Step (1) Ligand Preparation: The tecovirimat ligand (PubChem CID: 16124688) was acquired from the PubChem database (Kim et al., 2025). The ligand file was prepared through protonation followed by energy minimization, and the 3D structures were aligned in their most stable conformer to establish the ligand library.
Step (2) Protein Preparation: The modelled enzymes and their corresponding SNP PDB files were prepared by removing water molecules, metal ions, duplicate subunits, and any additional ligands. The proteins were then protonated as the final preparatory step.
Step (3) Molecular Docking Protocol: Docking was performed following a rigid receptor protocol. Ligand placement was executed using the Triangle Matcher algorithm (timeout: 300 s), generating an initial pool of 1,000 poses.
Step (4) Initial Scoring & Refinement: These initial poses were scored using the London dG function, and the top 30 unique poses were retained. Structure refinement was conducted using the Forcefield method (pocket residues included within a 6 Å cutoff; side chains tethered with a strength of 10). The potential setup utilized the reaction field, with final energy calculated using Generalized Born/Volume Integral (GB/VI). The refinement termination criteria were set to a gradient of 0.01 or a maximum of 500 iterations.
Step (5) Final Evaluation & Visualization: Final ranking of the docked poses was determined using the GBVI/WSA dG scoring function. The top 30 unique poses were retained for analysis, with scores expressed in kcal/mol. BIOVIA Discovery Studio 2021 (V21.1.0.20298) (Dassault Systèmes, , 2021) was utilized for 2D/3D visualization. Finally, root mean square deviation (RMSD) values were computed between each ligand-wild-type docking pose and the corresponding ligand-SNP docking pose for subsequent analysis.

### 2.6 Determination of protein’s structural and physicochemical parameters changes upon mutation

Mutations that affect the protein’s 3D conformation could alter its function. Hence, the HOPE (Venselaar et al., 2010) and DynaMut2 (Rodrigues et al., 2021) tools were utilized independently to predict the structural changes corresponding to the selected mutants.

The HOPE tool was utilized to conduct a comprehensive analysis of mutant proteins. The system computed the proteins 3D coordinates. Also, it compiled sequence annotations from the UniProt database and collected data from the Reproof software (Venselaar et al., 2010). The server required the amino acid sequence of the protein alongside information on the location as well as the type of mutation prior to doing the analysis.

The DynaMut2 tool was used to analyze protein dynamics, flexibility, and amino acid interactions. The algorithm provided optimized graph-based signatures alongside normal mode parameters to assess the influence of point mutations on protein stability (Rodrigues et al., 2021).

The ProtParam server (Walker, 2005) was utilized to evaluate the effects of mutations on protein physicochemical parameters. The calculated parameters included molecular weight, theoretical isoelectric point (pI), atomic composition, extinction coefficient, instability index, aliphatic index, and grand average of hydropathicity (GRAVY).

### 2.7 Primers designed for further *in vitro*/*in vivo* investigation

In our efforts to enhance the clinical relevance of the study results, specific PCR primers were designed to detect the identified most deleterious missense SNPs in the UGT1A1/4 genes using the Primer-BLAST Tool (NIH) (Supplementary Table S8). Primers were designed targeting the *Homo sapiens* genome with the following operational parameters: PCR product size was set between 70 and 104 bp; Primer melting temperatures (Tm) were constrained to a minimum of 57 °C, an optimum of 60 °C, and a maximum of 63 °C, with a maximum Tm difference of 3 °C between primer pairs; and Primer specificity was verified against the RefSeq mRNA database for *Homo sapiens*.

### 2.8 Evaluation of non-coding SNPs (5′ UTR and 3′ UTR variant)

The functional significance of non-coding variants was assessed using the RegulomeDB tool (Dong et al., 2023). This tool scores SNPs based on integrated high-throughput data, including ENCODE project datasets. The scores rank variants from 1a (strongest evidence for regulatory function) to 7 (minimal evidence), indicating their potential to impact transcription factor binding or gene expression (Supplementary Table S11).

The tool Polymorphisms in microRNAs and their target sites (PolymiRTS) (Bhattacharya et al., 2014) was utilized to analyze the non-coding SNPs associated with the UGT1A1/4 genes. The output included each SNP influencing miRNA, accompanied by their rsIDs and the corresponding miR IDs of the affected miRNAs. There were four PolymiRTS classes: “D” (the derived allele disrupts a conserved miRNA site), “N” (the derived allele disrupts a non-conserved miRNA site), “C” (the derived allele creates a new miRNA site) and “O” (other cases when the ancestral allele cannot be determined unambiguously).

## 3 Results

Uridine diphosphate glucuronosyltransferase 1A1/4 (UGT1A1/4) are the principal enzymes involved in the metabolism of the Mpox antiviral agent tecovirimat. In our investigation of the UGT1A1/4 missense SNPs and their association with enzyme-drug interactions, multiple levels of analysis were conducted. The initial layer entailed the preliminary exclusion of missense SNPs anticipated to have no deleterious impact. Consequently, we excluded the missense SNPs that are unlikely to influence enzyme integrity, activity, regulation, or enzyme-antiviral binding. This was additionally enhanced by the identification and validation of missense SNPs that may modify antiviral-protein interactions. Subsequently, an analysis of the structural and conformational stability of mutant proteins was conducted. The functional activity and regulation of mutant enzymes were also predicted. Finally, the modelling of the most detrimental SNPs and molecular docking experiments were done.

### 3.1 Data retrieval and *in-silico* determination of the deleteriousness of the missense SNPs

After filtering out data falling outside our study aims, the ENSEMBL search recognized 842 SNPs for UGT1A1 and 700 SNPS in UGT1A4.

**FIGURE 2:**
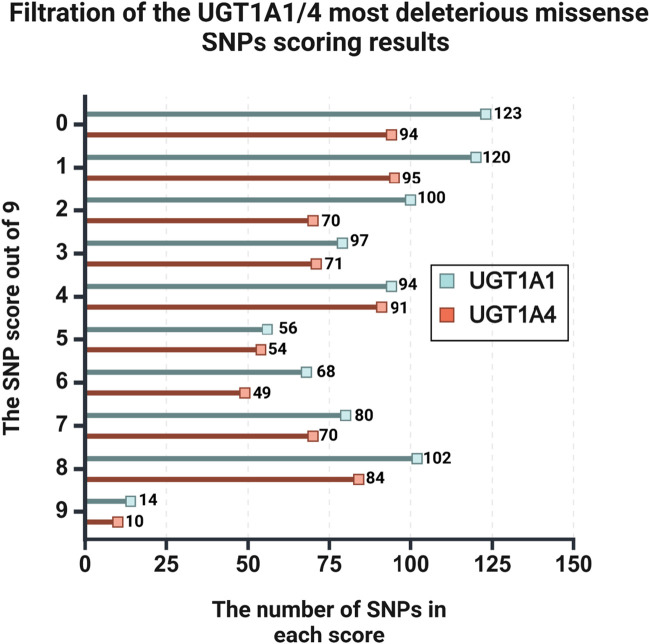
The chart shows the filtration of the UGT1A1/4 most deleterious missense SNPs scoring results. Illustrating the number of SNPs in each deleterious category ranging from 0 “o out of 9 = Benign” to 9 “9 out of 9 = Deleterious”.

14 UGT1A1 SNPs were identified, namely (L50R, G276R, G276C, G276D, G276V, G308R, P356T, G374S, G385S, P387S, P387R, P392S, G470S and G470D) and 10 SNPs in UGT1A4 which were (G309R, G309E, P357T, G375S, G386S, P388S, P388R, P393L, G471S and G471D). The deleteriousness analysis results of all the missense SNPs in UGT1A1/4 were illustrated in (Figure 2) and summarized in (Supplementary Tables S1, 13, 14). Additionally, the allelic frequency of these SNPs is reported in (Supplementary Table S2). The information on SNP frequencies was obtained from the databases dbSNP (Sherry et al., 2001) and gnomAD (Chen et al., 2024) by using the variants’ IDs.

The variants that passed this rigorous filtering criteria are considered strong high-confidence deleterious candidates that will be subjected to more subsequent analysis.

### 3.2 Determination of stability upon mutation and evolutionary conservation analysis

The I-Mutant findings illustrated that all SNPs under investigation showed a decrease in structural stability following mutation except three SNPs, namely, UGT1A1-G276C, UGT1A4-G309E and UGT1A4-P393L which were predicted to have higher stability following mutation. The MuPro findings indicated that all SNPs lowered structural stability owing to the mutation except UGT1A4-P393L SNP that showed increased stability. We excluded the SNPs that were predicted to potentially increase stability upon mutation. The 21 SNPs that showed potential decreased stability (13 in UGT1A1 and 8 in UGT1A4), were considered for subsequent validation as they are most likely to consequently affect the structural stability and function of enzymes. The results of the stability determination of the mutations of the UGT1A1/4 SNPs are listed in (Supplementary Table S3).

**FIGURE 3:**
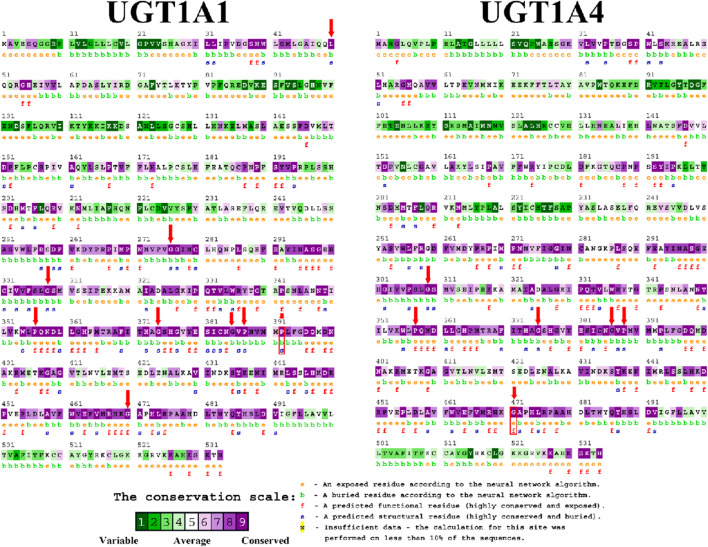
The diagram shows the Consurf results for UGT1A1/4 variants.

The UGT1A1/4 Consurf conservation analysis report is shown in (Figure 3). In UGT1A1/4, all amino acids of the missense SNPs under investigation were “highly conserved”, having a score between 8 or 9, This observation supports the biological premise that evolutionarily conserved residues are intolerant to mutations, which can often be pathogenic. Additionally, all amino acids of the studied SNPs were predicted to be Buried/Structural, except for UGT1A1-P356, UGT1A1- G470, UGT1A4- P357 and UGT1A4-G471 which were determined to be Exposed/Functional. Results of the evolutionary conservation analysis for the UGT1A1/4 SNPs are summarized in (Supplementary Table S4).

### 3.3 Binding site prediction using COACH

An additional layer of analysis was performed to examine the UGT1A1/4 enzyme binding cavity and the effects of amino acid alterations within by using the COACH package. The data showed that some mutated residues could have a deleterious effect by being involved in the enzyme’s binding site. SNPs in amino acids inside the binding cavity of UGT1A1 were identified in (G308, P356, G374) and those identified in the binding cavity of UGT1A4 were: (G309, P357, G375). Only SNPs in these amino acids were selected for the subsequent verification steps. COACH data results are shown in (Supplementary Table S5).

### 3.4 Modelling and model validation of the most deleterious UGT1A1/4 SNPs

SWISS-MODEL was employed to generate models of UGT1A1/4 wild-type proteins and their SNPs for molecular docking, assessing the influence of these SNPs on drug binding mode and affinity. While AlphaFold provides excellent structural predictions for wild-type proteins, homology modeling was deliberately selected for this study. The primary objective was to comparatively analyze structural deviations induced by specific missense SNPs. By utilizing SWISS-MODEL with high-identity templates (up to 100% for UGT1A1 and ∼89.5% for UGT1A4), we ensured that both the wildtype and mutant structures were generated using the exact same modeling pipeline and baseline spatial constraints. This methodological consistency is crucial; it guarantees that the subtle structural and docking deviations observed in the mutant models are strictly attributable to the single amino acid substitutions, rather than algorithmic variations that might occur if using separate predictive runs or different predictive architectures for the wildtype versus the SNPs. The predicted models required validation and verification for reliable use. All six UGT1A1/4 SNP FASTA files, along with the wild type, were uploaded to Swissmodeler. All PDB files for the models were acquired, downloaded, and subsequently underwent additional validation procedures prior to application.

The validation algorithms and metrics applied to the UGT1A1/4 models' PDB files demonstrated that all models' validation parameters were within acceptable ranges, rendering them appropriate for our study. To ensure the reliability of the subsequent molecular docking, the structural quality of the generated SWISS-MODEL templates was rigorously assessed. For the UGT1A1 models (wildtype and SNPs), template sequence identity was exceptionally high, ranging from 99.81% to 100% utilizing the P22309.1.A template. The UGT1A4 models utilized the A0A2K6QNT7.1.A template, showing strong sequence identities between 89.33% and 89.51%. Global Model Quality Estimates (GMQE) were robust across all models, scoring 0.91 for UGT1A1 variants and 0.92 for UGT1A4 variants, indicating high confidence in the template alignments. Furthermore, ERRAT scores demonstrated high overall model quality (94.88–95.27 for UGT1A1; 92.98–93.95 for UGT1A4), while PROSA Z-scores ranged from −8.42 to −8.59, confirming the models' structural features are well within the range of native proteins. Finally, MolProbity geometric assessments confirmed proper stereochemistry, with 96.05%–97.74% of residues situated in Ramachandran favored regions and negligible Ramachandran outliers (0.19%–0.56%). The model validation outcomes for UGT1A1/4 are presented in (Supplementary Table S6).

### 3.5 Molecular docking

**FIGURE 4:**
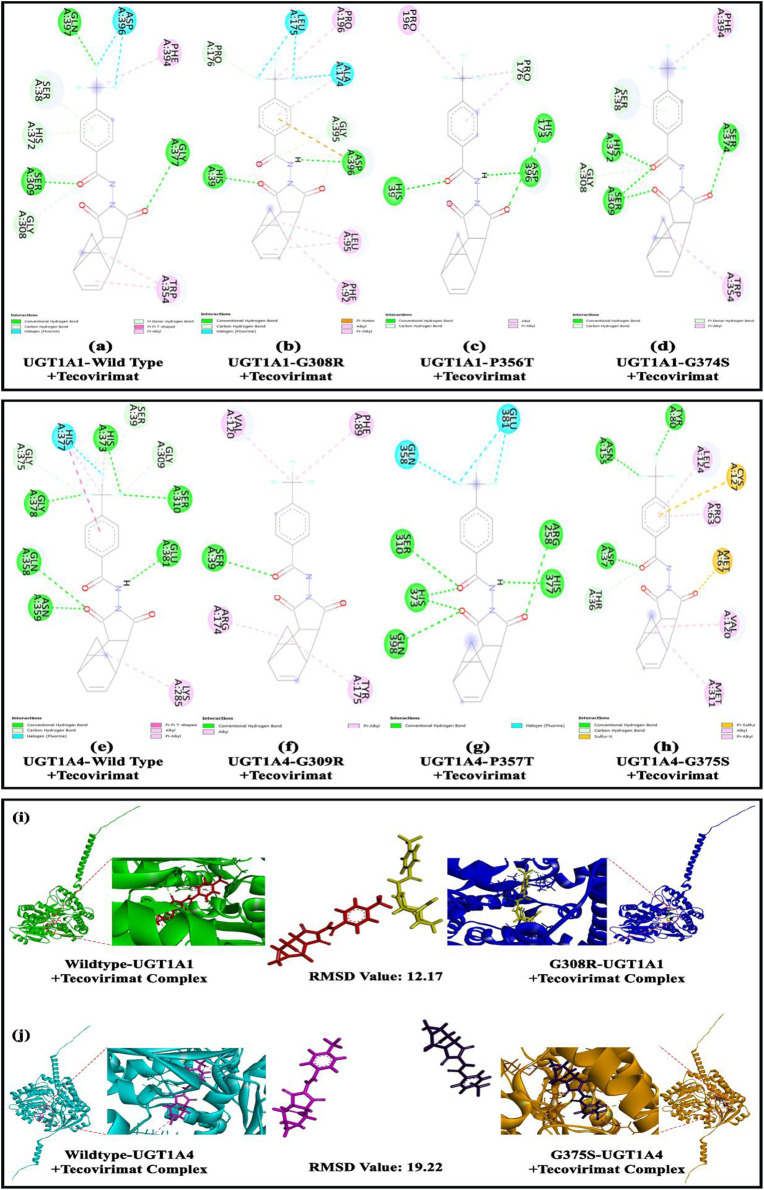
Drug-protein docking poses of wildtype UGT1A1/4 and their most deleterious SNPs with Tecovirimat. **(a)** UGT1A1-Wildtype **(b)** UGT1A1-G308R **(c)** UGT1A1-P356T **(d)** UGT1A1-G374S **(e)** UGT1A4-Wildtype **(f)** UGT1A4-G309R **(g)** UGT1A4-P357T **(h)** UGT1A4-G375S. The figure also illustrates the root mean square deviations (RMSD) value of tecovirimat docking pose with the SNPs scoring the lowest S score in both UGT1A1 and UGT1A4 in comparison with the drug’s docking pose with the wildtype of the same enzymes. **(i)** UGT1A1-G308R RMSD value: 12.17 **(j)** UGT1A4-G375S RMSD value: 19.22.

The make-up of the binding cavity identified by the MOE software is shown in (Table 1). The output of the docking analysis of UGT1A1/4 SNPs with the ligand drug tecovirimat are presented in (Table 2) and illustrated in (Figure 4). The docking S scores for the ligand-wild-type enzyme were −6.230 for tecovirimat-UGT1A1 WT and −6.049 for tecovirimat-UGT1A4 WT. Regarding the docking scores of SNPs-tecovirimat complexes, the UGT1A1 SNPs exhibited the following scores: G308R: −5.573, P356T: −6.109, and G374S: −5.716. In UGT1A4 SNPs, the S scores were recorded as follows: −5.804 for G309R, −5.923 for P357T, and −5.523 for G375S. A comprehensive analysis of the binding interactions between the antiviral drug tecovirimat and the WT forms of UGT1A1/4, alongside their respective SNPs variants can be seen in (Table 3). It details the docking S scores, specific hydrogen bond and hydrophobic interactions, and key deviations from the WT binding profile for select complexes.

**TABLE 1:** The make-up of the binding cavity identified by the MOE software for UGT1A1/4.

UGT1A1/cavity used for docking
Ile33, Pro34, Val35, Asp36, Gly37, Ser38, His39, Leu41, Ser42, Leu60, Ala61, Pro62, Ser65, Ile68, Tyr79, Phe83, Val88, Ser91, Phe92, Leu95, Asn98, Val99, Glu101, Asp103, Arg108, Val109, Lys111, Thr112, Tyr113, Lys115, Ile116, Asp119, Ser120, Leu123, Leu124, Gly126, Cys127, Leu130, Thr150, Asp151, Phe153, Leu154, Cys156, Ser157, His173, Ala174, Leu175, Pro176, Cys177, Ser178, Pro196, Leu197, Gln219, Leu222, Cys223, Val226, Tyr227, Tyr230, Arg257, Ser306, Leu307, Gly308, Ser309, Met310, Val311, Arg336, Trp354, Leu355, Pro356, Gln357, Phe369, Thr371, His372, Gly374, Ser375, His376, Gly377, Glu380, Leu393, Phe394, Gly395, Asp396, Gln397, Met398, Asp399, Asn400, Leu416

UGT1A4/cavity used for docking
Val34, Pro35, Thr36, Asp37, Gly38, Ser39, Pro40, Leu42, Leu61, Thr62, Pro63, Val65, Met67, Ile69, Tyr80, Phe89, Val92, Thr93, Tyr96, Met117, Val120, Ser121, Ala123, Leu124, Cys127, Cys128, Leu131, Asp152, Val154, Asn155, Leu156, Cys157, Trp173, Arg174, Tyr175, Ile176, Pro177, Leu180, Leu197, Leu198, Leu220, Ile223, Cys224, Phe227, Ser228, Tyr231, Arg258, Ser307, Leu308, Gly309, Ser310, Met311, Val312, Trp355, Pro357, Gln358, His373, Gly375, Ser376, His377, Gly378, Glu381, Phe395, Gly396, Asp397, Gln398, Asn401

**TABLE 2:** Docking S scores (Kcal/mol) of 47 different UGT1A1/4 wildtype and the most deleterious missense SNPs with tecovirimat antiviral drug.

Variant ID	Macromolecules		Tecovirimat docking score
		UGT1A1	
	Wildtype		−6.230
rs746111352	G308R		−5.573 (RMSD:12.17)
rs767850186	P356T		−6.109
rs1276913504	G374S		−5.716
		UGT1A4	
	Wildtype		−6.049
rs746111352	G309R		−5.804
rs767850186	P357T		−5.923
rs1276913504	G375S		−5.523 (RMSD: 19.22)

**TABLE 3:** Molecular Docking Profile of Tecovirimat with UGT1A1/4 Enzymes.

Enzyme Model	Docking S Score	Hydrogen Bonds (H-Bonds)	Hydrophobic Interactions	Key Differences from Wildtype
UGT1A1				
UGT1A1 WT (Reference)	-6.23	3 bonds: Ser309, Gly377, Gln397	8 interactions: Trp354, Gly308, His372, Ser38, Asp396, Phe394 Types: Carbon-Hydrogen, Pi-Donor Hydrogen, Pi-Pi T-shaped, Pi-alkyl, Halogen (Fluorine)	Baseline for comparison
G308R	-5.573	2 bonds: His39, Asp396	11 interactions: Phe92, Leu95, Gly395, Ala174, Pro196, Leu175, Pro176 New Type: Pi-Anion	-Bonds: Lost all 3 original bonds; formed 2 new ones with different residues. -Hydrophobic: Lost all 8 original interactions; formed 11 new ones with completely different residues. -Introduced a Pi-Anion interaction. -This SNP exhibited the lowest docking S score, prompting an analysis of its docking pose RMSD value, which was determined to be 12.17. An RMSD value of that magnitude signifies a lower degree of similarity and a greater degree of deviation.
P356T	-6.109	3 bonds: His173, Asp396, His39	4 interactions: Pro176, Pro196	-H-Bonds: Lost all 3 original bonds; formed 3 new ones with different residues. -Hydrophobic: Complete loss of the 8 WT interactions; established only 4 new ones.
G374S	-5.716	4 bonds: Ser309 (retained & new), His372, Ser374	4 interactions: Trp354, Gly308, Ser38, Phe394	-H-Bonds: Increased from 3 to 4. Lost bonds with Gly377 & Gln397 but retained one with Ser309 and added 3 new bonds. -Hydrophobic: Lost half (4 of 8) of the WT interactions.
UGT1A4				
UGT1A4 WT	-6.049	6 bonds: Asn359, Gln358, Glu381,	9 interactions: Lys285, Gly375, His377, His373, Ser39, Gly309 Types: Carbon-	Baseline for comparison
(Reference) G309R	-5.804	Ser310, Gly378, His373 1 bond: Ser39	Hydrogen, Pi-Pi T-shaped, Pi-alkyl, Alkyl, Halogen (Fluorine) 4 interactions: Tyr175, Arg174, Phe89, Val120	-H-Bonds: Lost all 6 original bonds; formed only 1 new one. -Hydrophobic: Forfeited all 9 original interactions; established only 4 new ones with different residues.
P357T	-5.923	6 bonds: Ser310 (retained), His37, Arg258, His377, Gln398	3 interactions: Glu381, Gln358 Type: Halogen only	-H-Bonds: Maintained 6 bonds. Retained the bond with Ser310 but lost the other 5, replacing them with new ones. -Hydrophobic: Lost most original interactions, retaining only 3 Halogen interactions.
G375S	-5.523	3 bonds: Asp37, Tyr80, Asn155	7 interactions: Met311, Val120, Met67, Pro63, Cys127, Leu124, Thr36 New	-H-Bonds: Lost all 6 original bonds; formed only 3 new ones.
			Types: Sulphur-X, Pi-sulfur	-Hydrophobic: Lost all 9 original interactions; formed 7 new ones. -Introduced Sulfur-X and Pi-sulfur interactions. -Among the three most deleterious UGT1A4 SNPs examined, This SNP exhibited the lowest docking S score, prompting an analysis of its docking pose RMSD value. The RMSD value of the G375S variant’s docking pose was 19.22.

The table provides a comparative analysis of the binding interactions between the antiviral drug tecovirimat and the wildtype (WT) forms of UGT1A1 and UGT1A4, alongside their respective single nucleotide polymorphism (SNP) variants. It details the docking S scores, specific hydrogen bond and hydrophobic interactions, and key deviations from the WT binding profile for select complexes.

### 3.6 Structural and physicochemical parameter changes

**FIGURE 5:**
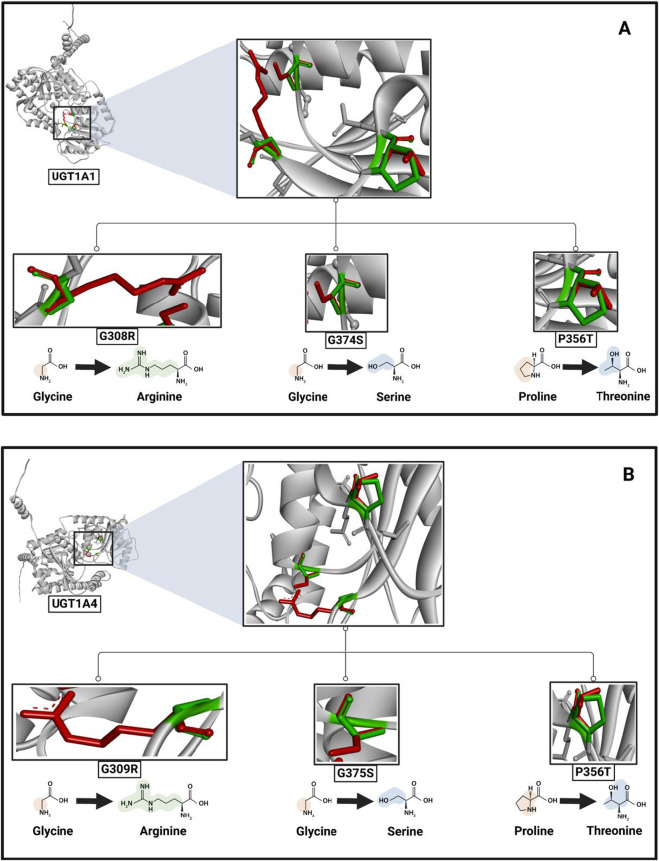
Structural changes associated with mutations of the UGT1A1/4 most deleterious SNPs. Wildtype residues are colored green and the mutated residues in red. **(A)** UGT1A1 enzyme cavity containing the three amino acids and their mutated SNPs **(B)** UGT1A4 enzyme cavity containing the three amino acids and their mutated SNPs. Note: This figure is provided purely as a static visual illustration of the side-chain substitutions within the binding cavities. The comprehensive mechanistic and biophysical generalizations discussed in the text were derived algorithmically via the Project HOPE and DynaMut2 analysis servers.

The tools HOPE and DynaMut2 were used to determine the structural changes associated with the mutations in the validated most deleterious enzyme SNPs (Figure 5). Depicts the structural alterations in the 6 UGT1A1/4 most deleterious mutants. The summary of all observed structural changes in UGT1A1/4 SNPs can be seen in (Table 4) were detailed summary of changes in hydrogen, polar, hydrophobic, and ionic interactions for selected UGT1A1/4 SNPs relative to their WT can be seen.

**TABLE 4:** Analysis of alterations in intermolecular bonds resulting from single nucleotide polymorphisms in the UGT1A1/4 enzymes.

Enzyme	SNP (mutated residue)	Interaction type	Wildtype (WT) residue interactions	SNP (mutated residue) interactions	Net change
UGT1A1	G308R (glycine → arginine at position 308)	Hydrogen bonds	4 total - His372 (1) - Ser306 (1) - Arg336 (1) - Val311 (1)	2 total - His372 (1) - Ser306 (1)	Loss of 2 H-bonds - Lost interactions with Arg336 and Val311
		Polar	4 total - Ser306 (1) - Arg336 (1) - Met310 (1) - Val311 (1)	7 total - Arg336 (1) - Ser306 (2) - His372 (4)	Gain of 3 polar interactions - Lost interactions with Met310 and Val311 - Gained 4 new interactions with His372 - Gained 1 interaction with Ser306
	P356T (proline → threonine at position 356)	Hydrogen bonds	4 total - Asp359 (3) - Leu360 (1)	2 total - Leu360 (1) - Asp359 (1)	Loss of 2 H-bonds - Lost 2 interactions with Asp359
		Hydrophobic	9 total - Asp359 (1) - Leu286 (2) - Leu360 (2) - Phe290 (4)	2 total - Asp359 (1) - Leu286 (1)	Loss of 7 hydrophobic interactions - Lost 4 interactions with Phe290 - Lost 2 interactions with Leu360 - Lost 1 interaction with Leu286
	G374S (glycine → serine at position 374)	Hydrogen bonds	5 total - Gly377 (1) - Val378 (2) - His372 (2)	5 total: (Same as WT)	No change
		Ionic	4 total - His372 (1) - Val378 (1) - Gly377 (1) - His376 (1)	7 total - His372 (3) - Val378 (3) - Gly377 (1)	Gain of 4 interactions - Lost the 1 WT interaction with His376 - Gained 2 interactions with His372 - Gained 2 interactions with Val378
UGT1A4	G309R (glycine → arginine at position 309)	Hydrogen bonds	3 total - His373 (1) - Arg337 (1) - Ser307 (1)	4 total - Arg337 (2) - Ser307 (1) - Gly375 (1)	Gain of 1 H-bond - Lost interaction with His373 - Gained 1 new interaction with Gly375 - Gained 1 interaction with Arg337
​	​	Polar	2 total - Ser307 (1) - Arg337 (1)	8 total - Ser39 (1) - His377 (1) - Ser307 (2) - His373 (4)	Gain of 6 polar interactions - Lost interaction with Arg337 - Gained new interactions with Ser39 (1), His377 (1), and His373 (4) - Gained 1 interaction with Ser307
​	P357T (proline → threonine at position 357)	Hydrogen bonds	3 total - Leu361 (1) - Asp360 (2)	4 total - Asp360 (2) - Leu361 (2)	Gain of 1 H-bond - Gained 1 interaction with Leu361
		Polar	4 total - Asp360 (2) - Leu361 (2)	5 total - Asp360 (3) - Leu361 (2)	Gain of 1 polar interaction - Gained 1 interaction with Asp360
		Hydrophobic	9 total - Phe291 (4) - Leu361 (2) - Leu287 (2) - Asp360 (1)	2 total - Asp360 (1) - Leu287 (1)	Loss of 7 hydrophobic interactions - Lost interactions with Phe291 (4) and Leu361 (2) - Lost 1 interaction with Leu287
​	G375S (glycine → serine at position 375)	Hydrogen bonds	5 total - His373 (2) - Val379 (2) - Gly378 (1)	5 total: (Same as WT)	No change
		Polar	5 total - His373 (2) - Val379 (1) - Gly378 (1) - His377 (1)	7 total - Val379 (2) - His373 (4) - Gly378 (1)	Gain of 2 polar interactions - Lost interaction with His377 - Gained 2 interactions with His373 and 1 with Val379

The ProtParam web service was utilized to indicate impact of the missense variants on the enzymes’ physicochemical parameters. The results were recorded and summarized in (Supplementary Table S7). The results demonstrated that variants induced physicochemical alterations in the entire protein molecules. All six highly deleterious missense SNPs were observed to modify the protein’s molecular weight, atomic composition, instability index, and the grand average of hydropathicity. A shift in the crucial physicochemical properties can destabilize the enzyme-drug complex, thereby altering their association.

### 3.7 Evaluation of non-coding SNPs

The initial data retrieval from the ensemble database yielded 308 non-coding SNPs in UGT1A1, and 324 non-coding SNPs in UGT1A4. Some variants could have a deleterious effect or induce alterations in enzymatic functions; consequently, they were analyzed by the RegulomeDB server. Among these SNPs, the RegulomeDB ranking could be obtained for just 135 SNPs in UGT1A1, categorized as follows; rank 4 (69 SNPs), rank 5 (64 SNPs), rank 2b (15 SNPs), rank 1f (3 SNPs), and rank 3a (2 SNPs).

Additionally, 177 SNPs were recognized in UGT1A4 and were classified into the following ranks: rank 4 (76 SNPs), rank 5 (64 SNPs), rank 2b (32 SNPs), rank 1f (3 SNPs), and rank 3a (2 SNPs) (Supplementary Table S9). Most of these SNPs were anticipated to affect transcription factor binding sites and/or chromatin accessibility.

All SNPs that were obtained from the RegulomeDB were subjected to another layer of investigation using the PolymiRTS database. This was done to recognize the variants that could influence the magnitude, location, and duration of gene expression. The results revealed that only 12 variants, all of which were 3 prime UTR variants, have the potential to cause functional implications on different miRNA binding sites, namely, rs1042640, rs112908387, rs142810023, rs148128252, rs182019761, rs200041554, rs34895241, rs34942353, rs61757316, rs61757317, rs71539604 and rs78684540. The PolymiRTS results of the non-coding variants of UGT1A1/4 are listed in (Supplementary Tables 10,11).

## 4 Discussion

Tecovirimat is the first oral antiviral drug approved for treating human Mpox viral infection. It is a selective inhibitor of the Orthopoxviral VP37 protein (Karagoz et al., 2023). In 2002, it was identified as a potential anti-Orthopoxviral using molecular drug design. The drug was approved for use as an anti-Mpox treatment in the US (2018), Canada (2021), and Europe (2022). Tecovirimat showed notable antiviral efficacy and a marked safety profile in animal studies conducted under the “Animal Rule” protocol. Hence, tecovirimat could offer an effective solution to overcome the current Mpox outbreak. Although a few pharmacokinetics studies were performed to investigate the drug’s bioavailability, safety and efficacy, there is a notable lack in pharmacogenetic studies on tecovirimat.

UGT1A1/4 enzymes are the main drug metabolizing enzymes of tecovirimat (Tempestilli et al., 2024). In the liver, the drug undergoes hydrolysis and glucuronidation, leading to the formation of several pharmacologically inactive metabolites (Sim et al., 2012). The WT conformation of the protein influences the binding with the ligand drug. Genetic variations in the genes encoding UGT1A1/4 enzymes could alter the structure and interactions with drugs, possibly affecting substrate-binding affinity and enzymatic function (Tang and Thomas, 2016).

Although several UGT1A1/4 polymorphisms have been reported (Adzhubei et al., 2013), there were limited data available regarding their impact on the therapeutic outcome of the metabolized drugs. Moreover, there is an absence of tecovirimat pharmacogenetic studies that potentially affects drug response in Mpox infected patients. Hence, there is a vital need to explore the missense SNPs that could alter tecovirimat metabolism and its response in these patients.

In this work, we designed a multilayered computational framework to investigate the interactions between tecovirimat and its drug-metabolizing enzymes, UGT1A1/4. This was done to predict the discrepancies in treatment efficacy and safety profile of the antiviral drug in Mpox patients. We conducted a thorough multistep *in silico* analysis to assess the impact of the missense SNPs on the UGT1A1/4 enzymes associated with the metabolism of the antiviral tecovirimat. Native conformation of the enzyme could influence the proper binding with the ligand drug and thereby the rate and extent of its metabolism (Manfredi et al., 2022). Moreover, we catalogued the deleterious missense SNPs identified to improve the clinical applicability of the study findings. Additionally, we designed and validated specific PCR primers for detecting the most highly deleterious missense SNPs identified in the UGT1A1/4 genes. Providing these primer sequences serves as a direct, practical bridge between our current *in silico* findings and future empirical research. We specifically engineered these tools to facilitate planned *in vitro* enzymatic assays and targeted clinical genotyping studies, streamlining the essential next steps required to translate these computational predictions into validated, patient-based pharmacokinetic profiles.

A collection of 9 benchmarked computational techniques was used to assess the deleteriousness of all SNPs examined. We implemented rigorous criteria; a mutation was deemed detrimental if it received a score of 9 out of 9 (picked up by all 9 tools used) a score representing the highest predicted likelihood of a deleterious effect. Only 14 SNPs in the *UGT1A1* gene alongside 10 in the *UGT1A4* gene were identified as highly deleterious from a total of 842 SNPs in *UGT1A1* and 700 in *UGT1A4* genes. This suggested that around 1.6% of the missense mutations in *UGT1A1* and about 1.4% in *UGT1A4* are highly deleterious. This was a key methodological decision in our study which was the use of a stringent 9/9 consensus score to define a variant as deleterious. We implemented this rigorous criterion to generate a high-confidence list of candidate variants and reduce the likelihood of carrying false positives into further analysis.

However, we recognize that this approach has significant limitations, most notably a potentially high false-negative rate, which may have led to the exclusion of genuinely deleterious variants that did not achieve unanimous prediction by all tools. Different *in silico* tools utilize distinct algorithms and training datasets, and a lack of consensus does not necessarily equate to a benign impact. Despite the varying predictive performances and specificities of these individual tools, an equal weighting approach was deliberately employed. In the absence of a large, experimentally validated dataset of UGT1A1/4-specific mutations to calculate precise tool-specific weights, applying generalized weights could introduce unwarranted bias. By weighting all tools equally and requiring absolute consensus, we mitigated the inherent algorithmic biases of any single program, ensuring that the variants prioritized are those robust enough to trigger diverse predictive criteria. Therefore, the variants identified in this study should be viewed as a conservative shortlist of the most confidently predicted deleterious mutations, rather than a comprehensive catalogue. Future studies aiming for a more exhaustive discovery of pathogenic variants could consider a less stringent threshold (e.g., a majority vote) or a weighted model that accounts for the relative accuracy of each predictive tool.

Our study utilized a panel of established *in silico* tools to predict the functional impact of specific missense variants in the UGT1A1/4 genes. We acknowledge that these predictions, which are based primarily on the biochemical consequences of amino acid changes, are strengthened by the integration of orthogonal evidence. This framework builds a more robust case for a variant’s pathogenicity by combining independent lines of evidence, such as population genetics, clinical data, and evolutionary conservation. A key piece of such evidence is allele frequency from large-scale population databases. Variants that are functionally deleterious are subject to negative selective pressure and are therefore expected to be rare in the general population. In support of our *in silico* findings, we analyzed the global minor allele frequency (GMAF) for the variants identified in our study. All the predicted deleterious SNPs for which frequency data were available are exceptionally rare, with a GMAF of < 0.001. This low frequency provides strong, independent support for the hypothesis that these variants are indeed deleterious and could be functionally significant. This convergence of evidence where a variant is predicted to be damaging and is found to be rare in the population strengthens the confidence of our initial assessment and underscores the value of integrating multiple evidence types for variant interpretation. To assess the potential clinical burden of these variants, we examined their global prevalence. Notably, the variant [rs767850186 “2-234676564-C-A”] shows a high minor allele frequency (<0.00006169) in African population compared to the global average. This suggests that the African population patients receiving tecovirimat may be at a disproportionately higher risk of altered drug metabolism and potential toxicity keeping in mind that Mpox is endemic in Africa (Huang et al., 2022). This population-specific insight suggests that future clinical studies should evaluate the potential benefits of targeted genotyping in this demographic group prior to antiviral administration.

A comprehensive bioinformatics analysis was performed to elucidate the underlying deleterious mechanisms of these SNPs. The SNPs, recognized as deleterious, were subject to an evaluation of structural integrity and stability post-mutation. To this end, two computational tools were used, the I-Mutant and MuPro. The I-Mutant analysis of the 24 UGT1A1/4 SNPs indicated that most mutations resulted in a lowered structural stability, except three SNPs which were predicted to increase rigidity following mutation. The MuPro results demonstrated that all SNPs reduced structural stability due to the mutations, except one SNP, which exhibited enhanced stability. Of note, the protein’s geometry is maintained by a specific set of bonds and balanced molecular interactions. The smallest alteration in such bonds could hinder the proper conformation of the protein and compromise its flexibility. Collectively, these data demonstrated that 21 out of 24 SNPs were classified as deleterious due to a significant alteration in enzyme structural integrity and stability after mutation. The 21 SNPs that exhibited a reduction in stability were selected for further analysis.

The ConSurf tool was used to analyze the evolutionary conservation of the amino acids of UGT1A1/4 enzymes. All amino acids of the missense SNPs under investigation were “highly conserved” showing scores of 8 or 9. Additionally, all amino acids of the studied SNPs were predicted to be “Buried/Structural”, except for UGT1A1-P356, UGT1A1- G470, UGT1A4-P357 and UGT1A4-G471 which were determined to be “Exposed/Functional”. It was sensible that most of the residues examined were highly conserved since mutations in a conserved area of a protein are likely to result in adverse effects on the protein’s structure and/or its function (Capriotti and Fariselli, 2017).

Subsequently, the COACH tool was used to examine the UGT1A1/4 enzyme binding cavity and the potential impact of amino acid modifications. The findings indicated that some mutated residues may adversely affect the drug-enzyme interaction as they were required for the proper conformation of the drug’s binding site. SNPs in amino acids of the binding cavity of UGT1A1 were (G308, P356, G374) and inside the binding cavity UGT1A4 were (G309, P357, G375). These six SNPs underwent subsequent verification stages. Our rationale was that these six UGT1A1/4 SNPs were identified as the most deleterious by nine distinct tools, predicted to compromise structural stability by two separate programs, observed in an evolutionarily conserved region of the protein, and located within the enzyme’s binding site. Hence, they could interfere with proper binding with the ligand drug tecovirimat.

Molecular docking was conducted for each mutant with the antiviral drug tecovirimat, to thoroughly investigate the underlying cause of the detrimental impact of the selected SNPs. Each wild-type protein-drug complex may exhibit multiple predicted poses, with the pose that produced the best docking S score for the specified drug being designated as the reference. We analyzed the ligand molecular interactions and bonding between the WT and mutant proteins in a natural simulation of protein-drug interactions. Structural changes linked to the mutations, as well as the altered intermolecular interactions could be potential contributors to the deleterious effects. It is important to note that the absolute differences in docking S scores between the wild-type and mutant complexes (e.g., −6.23 vs. −5.57 kcal/mol) are relatively modest. In molecular docking, such numerical differences alone are insufficient to definitively establish altered metabolism. However, these modest score shifts were accompanied by profound qualitative changes in the binding profiles. Rather than relying solely on binding affinity estimates, our prediction of functional disruption is primarily driven by the observed loss of critical wildtype hydrogen bonds, the reorganization of the hydrophobic interaction network, and deviations in the ligand’s spatial orientation within the catalytic pocket.

A detailed analysis of the structural changes introduced by these mutations reveals shared mechanistic patterns across both enzymes, driven by the specific biophysical properties of the substituted amino acids.

Glycine-to-Arginine Substitutions (UGT1A1-G308R and UGT1A4-G309R): Both variants involve the replacement of a neutral, highly conserved glycine with a positively charged arginine. The WT residue, glycine, is the most flexible of all amino acids (Senthil et al., 2019). Glycine exhibits distinctive torsional angles due to its high conformational flexibility, which is often necessary for the protein’s proper function and local geometry. Replacing this small, flexible residue with a bulky, positively charged arginine introduces steric hindrance and potential electrostatic repulsion with the ligand or neighboring residues of the same charge. Thus, mutation into another residue could force the local backbone into an unfavorable conformation that disturbs the local structure and abolishes its function. Proline-to-Threonine Substitutions (UGT1A1-P356T and UGT1A4-P357T): These mutations result in the replacement of a rigid proline with a polar threonine. Proline is known for its rigidity and its ability to induce a unique backbone conformation that might be crucial at this position (Bromberg and Rost, 2007). The substitution of proline with threonine could disrupt this special configuration, potentially affecting the protein’s stability and function or causing misfolding. Furthermore, proline is more hydrophobic than threonine; its substitution leads to a marked loss of the stabilizing hydrophobic interactions in the protein core or surface. Glycine-to-Serine Substitutions (UGT1A1-G374S and UGT1A4-G375S): Similarly, replacing the highly flexible WT glycine with a larger serine reduces the enzyme’s functional dynamics. While serine is relatively small, it lacks the unique rotational freedom of glycine. This substitution limits the specific torsion angles available to the polypeptide chain, potentially introducing a kink in the protein’s geometry and forcing the local backbone into an unfavorable conformation that impairs structural integrity.

Analysis of these structural changes could explain the observed differences in hydrogen bonds formation with neighboring residues. Additionally, it could shed light on the deviation seen in molecular docking parameters including docking scores and docking poses between the wildtype complex and that of the SNPs.

Of the three most deleterious UGT1A4 SNPs examined, G375S exhibited the lowest docking S score. More importantly, the RMSD value of the G375S variant’s docking pose compared to the wildtype was 19.22 Å. Similarly, the UGT1A1-G308R variant exhibited an RMSD of 12.17 Å. Biologically, deviations of this magnitude are highly significant. For UGT enzymes to successfully catalyze glucuronidation, the substrate must be positioned with precise geometry relative to the catalytic residues and the co-substrate. An RMSD of >12 Å indicates a drastic spatial reorientation of tecovirimat within the binding cavity. Consequently, even if the mutant enzyme retains some thermodynamic binding affinity for the drug (as reflected by the modest changes in S scores), the binding is likely non-productive, physically preventing the metabolic reaction from occurring.

Analysis of the physicochemical characteristics was done by both the HOPE and DynaMut2 tools. Results showed disparities in the structural characteristics between the wildtype and mutated residues, suggesting potential causes for structural instability and functional variation. Additionally, The ProtParam tool showed that the mutations resulted in alterations throughout the entire protein. All six highly deleterious missense SNPs were predicted to change the protein’s molecular weight, atomic composition, instability index, and hydropathy. Disruption of the delicate physicochemical properties could create an imbalance that alters the enzyme-drug association.

Furthermore, identifying other variants in the non-coding region is sensible because these variants may induce regulatory alterations affecting the gene expression, phenotype. Also, such variations could have a direct influence on the enzyme-drug binding if they introduced global changes in the configuration of the polypeptide chain. Out of the retrieved variants from the ENSEMBL database only 135 in UGT1A1 alongside 177 in UGT1A4 received ranking by the RegulomeDB. The RegulomeDB server offered comprehensive insights into the functional implications of non-coding SNPs, encompassing data on overlapping transcription factor motifs and chromatin states. The ranking assigned by RegulomeDB indicated the comprehensiveness of data available for each SNP. Our investigation classified most non-coding SNPs in the *UGT1A1/4* genes as ranks 4 and 5. This suggested that many SNPs influenced transcription factor binding sites and chromatin accessibility, indicating their impact on gene expression. All SNPs derived from the RegulomeDB analysis have undergone further evaluation using the PolymiRTS database to assess the effects of variants that may affect the intensity, localization, and frequency of gene expression. The results revealed that only twelve variants, which were all 3′UTR variants, had supporting data. All these variants were categorized under the functional classification of D or C which means they had the potential to affect various miRNA binding sites functionally.

## 5 Conclusion

In this work, we identified six deleterious UGT1A1/4 SNPs (namely, G308R, P356T and G374S in UGT1A1, and G309R, P357T and G375S in UGT1A4) that could potentially have significant clinical implications for tecovirimat metabolism. Our findings predict that these mutations alter enzyme function and drug binding, potentially leading to altered drug response in Mpox patients. These predictions highlight the potential value of investigating pharmacogenetic-guided dosage adjustments to improve efficacy and minimize adverse effects, particularly in vulnerable populations. A limitation of our work is its focus on *in silico* predictions. Hence, future studies should employ molecular dynamics simulations to assess interaction stability. While our computational analysis may provide valuable predictions regarding potentially deleterious mutations, these findings are preliminary. It is crucial that they are followed by experimental validation through *in vitro* and *in vivo* studies to define the pharmacodynamic and pharmacokinetic profiles of tecovirimat in patients harboring these variants. Such functional assays and patient-based studies are the essential next steps to translate these computational insights into actionable diagnostic and therapeutic strategies.

## Data Availability

The datasets generated and/or analyzed during the current study are provided within the manuscript or supplementary information files.
