# Supplementary material for: Computational analysis and validation of UGT1A1/4 missense variants impacting tecovirimat metabolism in monkeypox patients
Source: Front Syst Biol. 2026 Jun 11;6:1821230. doi: 10.3389/fsysb.2026.1821230 (PMC13293890; doi:10.3389/fsysb.2026.1821230)
Supplement: Supplementary file 1 [file Table1.docx]

***Computational Analysis and validation of UGT1A1/4 Missense Variants Impacting Tecovirimat Metabolism in Monkeypox Patients***

**Amro A. Abdelazim^1^, Sameh E. Hassanein^1,2^*, Mohamad Maged^3^, Nada S. Al-Theyab^4^, Itoh Kimiko^5^, Kotb A. Attia^6^**

^1^International Dryland Development Commission (IDDC), Cairo, Egypt.

^2^ Bioinformatics Program, School of Biotechnology, Nile University, Giza, Egypt.

^3^Applied Biotechnology Program, School of Biotechnology, Nile University, Giza, Egypt.

^4^Department of Pharmaceutical Chemistry, College of Pharmacy, King Saud University, Riyadh 11451, Saudi Arabia ([naaltheyab@ksu.edu.sa](mailto:heyab@ksu.edu.sa))

^5^Institute of Science and Technology, Niigata University, Ikarashi-2, Nishiku, Niigata 950-2181, Japan ([kim@agr.niigata-u.ac.jp](mailto:kim@agr.niigata-u.ac.jp))

^6^Center of Excellence in Biotechnology Research, King Saud University, Riyadh 11451, Saudi Arabia

*Corresponding author: sibrahem@nu.edu.eg

| Table S1. The most deleterious UGT1A1/4 SNPs and their scoring using SIFT, PolyPhen, Mutation Assessor, PhD-SNP, SNAP, Meta-SNP, E-SNPs&GO, PANTHER and FATHMM (D= Disease causing/Deleterious/Damaging, P=Pathogenic). | | | | | | | | | | |
| --- | --- | --- | --- | --- | --- | --- | --- | --- | --- | --- |
| UGT1A1 Variant ID | **AA SNPs** | **sift** | **polyphen** | **mutation assessor** | **PhD-SNP** | **SNAP** | **Meta-SNP** | **E-SNPs  & GO** | **Panther** | **FATHMM** |
| UGT1A1 SNPs | | | | | | | | | | |
| rs2125984045 | **L50R** | D | D | high | D | D | D | P | D | D |
| rs72551345 | **G276R** | D | D | high | D | D | D | P | D | D |
| rs72551345 | **G276C** | D | D | high | D | D | D | P | D | D |
| rs570314042 | **G276D** | D | D | high | D | D | D | P | D | D |
| rs570314042 | **G276V** | D | D | high | D | D | D | P | D | D |
| rs746111352 | **G308R** | D | D | high | D | D | D | P | D | D |
| rs767850186 | **P356T** | D | D | high | D | D | D | P | D | D |
| rs1276913504 | **G374S** | D | D | high | D | D | D | P | D | D |
| rs1699597581 | **G385S** | D | D | high | D | D | D | P | D | D |
| rs901936528 | **P387S** | D | D | high | D | D | D | P | D | D |
| rs1559415403 | **P387R** | D | D | high | D | D | D | P | D | D |
| rs1286993592 | **P392S** | D | D | high | D | D | D | P | D | D |
| rs2126066747 | **G470S** | D | D | high | D | D | D | P | D | D |
| rs1187321852 | **G470D** | D | D | high | D | D | D | P | D | D |
| UGT1A4 SNPs | | | | | | | | | | |
| rs746111352 | **G309R** | D | D | high | D | D | D | P | D | D |
| rs62625011 | **G309E** | D | D | high | D | D | D | P | D | D |
| rs767850186 | **P357T** | D | D | high | D | D | D | P | D | D |
| rs1276913504 | **G375S** | D | D | high | D | D | D | P | D | D |
| rs1699597581 | **G386S** | D | D | high | D | D | D | P | D | D |
| rs901936528 | **P388S** | D | D | high | D | D | D | P | D | D |
| rs1559415403 | **P388R** | D | D | high | D | D | D | P | D | D |
| rs886043066 | **P393L** | D | D | high | D | D | D | P | D | D |
| rs2126066747 | **G471S** | D | D | high | D | D | D | P | D | D |
| rs1187321852 | **G471D** | D | D | high | D | D | D | P | D | D |

| **Table S2.**  **UGT1A1/4 deleterious SNPs with minor allelic frequency (Chr:bp= base pairs location on chromosome, gmaf= global minor allelic frequency, AA=amino acid, coord=coordinates/location, N/A=not available)** | | | | | | | | |
| --- | --- | --- | --- | --- | --- | --- | --- | --- |
| **Variant ID** | **SNPs** | **Location**  **Chr:bp** | **Alleles** | **gmaf_allele** | **gmaf_freq** | **Global MAF** | **AA** | **AA coord** |
| **UGT1A1** | | | | | | | | |
| **rs746111352** | **G308R** | 2:233767091 | G/A | A | 0.000004 | < 0.001 | G/R | 308 |
| **rs767850186** | **P356T** | 2:233767918 | C/A | A | 0.000297 | < 0.001 | P/T | 356 |
| **rs1276913504** | **G374S** | 2:233768255 | G/A | A | 0.000004 | < 0.001 | G/S | 374 |
| **rs2125984045** | **L50R** | 2:233760436 | T/G | G | 0.000004 | < 0.001 | L/R | 50 |
| **rs72551345** | **G276R** | 2:233761113 | G/C | C | **N/A** | **N/A** | G/R | 276 |
| **rs72551345** | **G276C** | 2:233761113 | G/T | T | **N/A** | **N/A** | G/C | 276 |
| **rs570314042** | **G276D** | 2:233761114 | G/A | A | 0.0002 | < 0.001 | G/D | 276 |
| **rs570314042** | **G276V** | 2:233761114 | G/T | T | 0.00009 | < 0.001 | G/V | 276 |
| **rs1699597581** | **G385S** | 2:233768288 | G/A | A | 0.000004 | < 0.001 | G/S | 385 |
| **rs901936528** | **P387S** | 2:233768294 | C/T | T | 0.000004 | < 0.001 | P/S | 387 |
| **rs1559415403** | **P387R** | 2:233768295 | C/G | G | **N/A** | **N/A** | P/R | 387 |
| **rs1286993592** | **P392S** | 2:233768309 | C/T | T | 0.000004 | < 0.001 | P/S | 392 |
| **rs2126066747** | **G470S** | 2:233772365 | G/A | A | **N/A** | **N/A** | G/S | 470 |
| **rs1187321852** | **G470D** | 2:233772366 | G/A | A | 0.000004 | < 0.001 | G/D | 470 |
| **UGT1A4** | | | | | | | | |
| **rs746111352** | **G309R** | 2:233767091 | G/A | A | 0.000004 | < 0.001 | G/R | 309 |
| **rs767850186** | **P357T** | 2:233767918 | C/A | A | 0.000297 | < 0.001 | P/T | 357 |
| **rs1276913504** | **G375S** | 2:233768255 | G/A | A | 0.000004 | < 0.001 | G/S | 375 |
| **rs62625011** | **G309E** | 2:233767092 | G/A | A | 0.000012 | < 0.001 | G/E | 309 |
| **rs1699597581** | **G386S** | 2:233768288 | G/A | A | 0.000004 | < 0.001 | G/E | 386 |
| **rs901936528** | **P388S** | 2:233768294 | C/T | T | 0.000004 | < 0.001 | P/S | 388 |
| **rs1559415403** | **P388R** | 2:233768295 | C/G | G | **N/A** | **N/A** | P/R | 388 |
| **rs886043066** | **P393L** | 2:233768310 | C/T | T | T=0.000019 | < 0.001 | P/L | 393 |
| **rs2126066747** | **G471S** | 2:233772365 | G/A | A | **N/A** | **N/A** | G/S | 471 |
| **rs1187321852** | **G471D** | 2:233772366 | G/A | A | 0.000004 | < 0.001 | G/D | 471 |

| **Table S3. Stability determination upon mutation of UGT1A1/4 SNPs using I-Mutant and Mu-Pro** | | | | | |
| --- | --- | --- | --- | --- | --- |
| **Variant ID** | **SNPs** | **I-Mutant** | | **MU-Pro** | |
|  |  | **Stability** | **I-Mutant DDG** | **Stability** | **MU-Pro DDG** |
| **UGT1A1** | | | | | |
| **rs2125984045** | **L50R** | Decrease Stability | -1.30 | Decrease Stability | -1.16 |
| **rs72551345** | **G276R** | Decrease Stability | -0.08 | Decrease Stability | -0.44 |
| **rs72551345** | ***G276C*** | *Increase Stability* | *0.03* | Decrease Stability | -0.26 |
| **rs570314042** | **G276D** | Decrease Stability | -0.33 | Decrease Stability | -0.27 |
| **rs570314042** | **G276V** | Decrease Stability | -0.48 | Decrease Stability | -0.44 |
| **rs746111352** | **G308R** | Decrease Stability | -0.41 | Decrease Stability | -0.59 |
| **rs767850186** | **P356T** | Decrease Stability | -1.27 | Decrease Stability | -1.26 |
| **rs1276913504** | **G374S** | Decrease Stability | -0.78 | Decrease Stability | -0.97 |
| **rs1699597581** | **G385S** | Decrease Stability | -0.33 | Decrease Stability | -0.75 |
| **rs901936528** | **P387S** | Decrease Stability | -2.08 | Decrease Stability | -0.96 |
| **rs1559415403** | **P387R** | Decrease Stability | -0.95 | Decrease Stability | -0.74 |
| **rs1286993592** | **P392S** | Decrease Stability | -1.10 | Decrease Stability | -0.77 |
| **rs2126066747** | **G470S** | Decrease Stability | -0.21 | Decrease Stability | -0.68 |
| **rs1187321852** | **G470D** | Decrease Stability | -0.90 | Decrease Stability | -0.61 |
| **UGT1A4** | | | | | |
| **rs746111352** | **G309R** | Decrease Stability | -0.41 | Decrease Stability | -0.59 |
| **rs62625011** | ***G309E*** | *Increase Stability* | *0.64* | Decrease Stability | -0.58 |
| **rs767850186** | **P357T** | Decrease Stability | -1.27 | Decrease Stability | -1.26 |
| **rs1276913504** | **G375S** | Decrease Stability | -0.78 | Decrease Stability | -0.97 |
| **rs1699597581** | **G386S** | Decrease Stability | -0.33 | Decrease Stability | -0.75 |
| **rs901936528** | **P388S** | Decrease Stability | -2.08 | Decrease Stability | -0.96 |
| **rs1559415403** | **P388R** | Decrease Stability | -0.95 | Decrease Stability | -0.74 |
| **rs886043066** | ***P393L*** | *Increase Stability* | *0.41* | *Increase Stability* | *0.39* |
| **rs2126066747** | **G471S** | Decrease Stability | -0.21 | Decrease Stability | -0.68 |
| **rs1187321852** | **G471D** | Decrease Stability | -0.90 | Decrease Stability | -0.61 |
| The stability is determined by calculating the difference in Gibbs free energy (DDG) between the wild-type and mutant proteins. This involves evaluating changes in interactions such as hydrogen bonds, van der Waals forces, and electrostatic interaction. The more negative (DDG) value is the less stable the mutant structure becomes. | | | | | |

| **Table S4. Conservation analysis results of UGT1A1/4 amino acids using Consurf** | | |
| --- | --- | --- |
| **Residues** | **Consurf score** | **Predictions** |
| **UGT1A1 residues** | | |
| **L50** | 9 | Buried/Structural |
| **G276** | 9 | Buried/Structural |
| **G308** | 9 | Buried/Structural |
| **P356** | 9 | Exposed/Functional |
| **G374** | 9 | Buried/Structural |
| **G385** | 8 | Buried/Structural |
| **P387** | 9 | Buried/Structural |
| **P392** | 9 | Buried/Structural |
| **G470** | 9 | Exposed/Functional |
| **UGT1A4 residues** | | |
| **G309** | 9 | Buried/Structural |
| **P357** | 9 | Exposed/Functional |
| **G375** | 9 | Buried/Structural |
| **G386** | 8 | Buried/Structural |
| **P388** | 9 | Buried/Structural |
| **G471** | 9 | Exposed/Functional |

| **Table S5. COACH Residue identifier for UGT1A1/4, Some of the SNPs under investigation occur in the amino acids within the enzyme's binding site and are highlighted in red.** | | | |
| --- | --- | --- | --- |
| **C-score** | **Cluster size** | **Lig Name** | **Consensus Binding Residues** |
| **UGT1A1** | | | |
| **COACH Results** | | | |
| 0.79 | 75 | UDP | 38,306,**308**,309,336,354,355,357,372,**374**,375,376,377,380 |
| **TM-SITE Results** | | | |
| 0.48 | 16 | UDP | 38,41,**308**,309,336,354,355,357,372,**374**,375,376,377,380,394 |
| **S-SITE Results** | | | |
| 0.34 | 8 | UDP | 38,41,287,290,306,**308**,309,310,336,337,338,354,355,**356**,357,372,**374**,375,376,377,380 |
| **COFACTOR Results** | | | |
| 0.49 | - | UDP | 38,306,309,336,354,355,357,372,**374**,375,376,377,380 |
| **FINDSITE Results** | | | |
| 0.41 | 14 | UDP | 37,38,39,41,306,**308**,309,310,336,354,355,357,372,**374**,375,376,377,380,394,396,397 |
| **ConCavity Results** | | | |
| 0.66 | - | - | 38,39,40,41,151,284,306,**308**,309,310,311,335,337,354,355,**356**,357,358,371,372,**374**,375,376,377,380,381,395,396,397 |
| **UGT1A4** | | | |
| **COACH Results** | | | |
| 0.68 | 78 | U2F | 39,42,174,**309**,310,337,355,356,358,373,**375**,376,377,378,381,395,396,397,398 |
| **TM-SITE Results** | | | |
| 0.48 | 18 | UDP, UDF | 38,39,307,**309**,310,311,337,339,355,356,358,373,**375**,376,377,378 |
| **S-SITE Results** | | | |
| 0.34 | 8 | UDP | 39,42,283,288,291,307,**309**,310,311,337,338,339,355,356,**357**,358,373,**375**,376,377,378,381 |
| **COFACTOR Results** | | | |
| 0.65 | - | UDP | **309**,310,337,355,356,358,373,376,377,378,381 |
| **FINDSITE Results** | | | |
| 0.41 | 14 | UDP | 38,39,40,42,285,307,**309**,310,311,337,355,356,358,373,**375**,376,377,378,381,395,397,398 |
| **ConCavity Results** | | | |
| 0.42 | - | - | 39,42,174,281,306,**309**,310,337,339,355,**357**,358,373,374,**375**,376,377,378,395,396,397,398,401 |
| -C-score is the confidence score of the prediction. C-score ranges [0-1], where a higher score indicates a more reliable prediction.  -Cluster size is the total number of templates in a cluster.  -UDP: Uridine-5'-Diphosphate, U2F: Uridine-5'-Diphosphate-2-Deoxy-2-Fluoro-Alpha-D-Glucose. | | | |

| **Table S6.** **SWISS-MODEL UGT1A1/4 models validation.** | | | | | | | | | | | | | | | | |
| --- | --- | --- | --- | --- | --- | --- | --- | --- | --- | --- | --- | --- | --- | --- | --- | --- |
| **Variant ID** | **Macromolecule** | **ERRAT & PROSA Scores** | | **QMEANDisCo** | | | | | | | | | **MolProbity** | | | |
|  |  | **ERRAT**  **score** | **PROSA Z score** | **Seq.Identity** | **Qmean** | **C-Beta(Cβ)** | **All Atoms** | **Solvation** | **Torsion** | **GMQE** | **QMEANDisCo Global** | **Template** | **MolProbity Score** | **Clash Score** | **Ramachandran Favoured** | **Ramachandran Outliers** |
| **UGT1A1** | | | | | | | | | | | | | | | | |
|  | **UGT1A1 Wildtype** | 95.0787 | -8.45 | 100 | 0.94 | 0.59 | 3.34 | 0.38 | 1.08 | 0.91 | 0.60 | P22309.1.A | 0.83 | 0.12 | 96.23% | 0.56% |
| **rs746111352** | **G308R** | 94.8819 | -8.44 | 99.81 | 1.12 | 0.70 | 3.29 | 0.40 | 1.24 | 0.91 | 0.59 | P22309.1.A | 0.99 | 0.59 | 96.05% | 0.56% |
| **rs767850186** | **P356T** | 95.0787 | -8.42 | 99.81 | 1.08 | 0.64 | 3.31 | 0.36 | 1.20 | 0.91 | 0.59 | P22309.1.A | 0.84 | 0.12 | 96.05% | 0.56% |
| **rs1276913504** | **G374S** | 95.2756 | -8.44 | 99.81 | 0.93 | 0.71 | 3.32 | 0.40 | 1.05 | 0.91 | 0.59 | P22309.1.A | 0.82 | 0.12 | 96.23% | 0.56% |
| **UGT1A4** | | | | | | | | | | | | | | | | |
|  | **UGT1A4 Wildtype** | 93.9571 | -8.56 | 89.51 | 0.79 | 0.39 | 3.50 | 0.53 | 1.02 | 0.92 | 0.58 | A0A2K6QNT7.1.A | 0.87 | 1.06 | 97.74% | 0.19% |
| **rs746111352** | **G309R** | 92.9825 | -8.59 | 89.33 | 0.92 | 0.55 | 3.52 | 0.55 | 1.14 | 0.92 | 0.58 | A0A2K6QNT7.1.A | 0.90 | 0.71 | 96.99% | 0.56% |
| **rs767850186** | **P357T** | 93.9571 | -8.52 | 89.33 | 0.88 | 0.39 | 3.49 | 0.55 | 1.12 | 0.92 | 0.58 | A0A2K6QNT7.1.A | 0.90 | 1.06 | 97.56% | 0.19% |
| **rs1276913504** | **G375S** | 93.7622 | -8.58 | 89.33 | 0.78 | 0.49 | 3.54 | 0.53 | 1.01 | 0.92 | 0.58 | A0A2K6QNT7.1.A | 0.87 | 1.06 | 97.74% | 0.19% |

| **Table S7. Disrupting Protein's Physicochemical Parameters of UGT1A1/4 SNPs in comparison with the wildtype Protein's Physicochemical Parameters.** | | | | | | | | | | |
| --- | --- | --- | --- | --- | --- | --- | --- | --- | --- | --- |
| **Variant ID** | **Macromolecules** | **Molecular**  **weight** | **Theoretical isoelectric point (pI)** | **Atomic composition** | **Total  -ve AA** | **Total +ve AA** | **Extinction**  **coefficients** | **Instability**  **index** | **Aliphatic**  **index** | **GRAVY** |
| **UGT1A1** | | | | | | | | | | |
|  | **Wild Type** | 59591.39 | 8.19 | C_2700_H_4222_N_710_O_748_S_31_ | 47 | 50 | 58955 | 34.84 | 95.63 | 0.064 |
| **rs746111352** | **G308R** | 59690.53 | 8.36 | C_2704_H_4231_N_713_O_748_S_31_ | 47 | 51 | 58955 | 36.02 | 95.63 | 0.057 |
| **rs767850186** | **P356T** | 59595.38 | 8.19 | C_2699_H_4222_N_710_O_749_S_31_ | 47 | 50 | 58955 | 33.97 | 95.63 | 0.066 |
| **rs1276913504** | **G374S** | 59621.42 | 8.19 | C_2701_H_4224_N_710_O_749_S_31_ | 47 | 50 | 58955 | 35.20 | 95.63 | 0.064 |
| **UGT1A4** | | | | | | | | | | |
|  | **Wild Type** | 60025.02 | 8.79 | C_2721_H_4253_N_723_O_744_S_32_ | 46 | 54 | 78435 | 28.16 | 94.38 | 0.040 |
| **rs746111352** | **G309R** | 60124.16 | 8.87 | C_2725_H_4262_N_726_O_744_S_32_ | 46 | 55 | 78435 | 29.35 | 94.38 | 0.033 |
| **rs767850186** | **P357T** | 60029.01 | 8.79 | C_2720_H_4253_N_723_O_745_S_32_ | 46 | 54 | 78435 | 27.30 | 94.38 | 0.042 |
| **rs1276913504** | **G375S** | 60055.05 | 8.79 | C_2722_H_4255_N_723_O_745_S_32_ | 46 | 54 | 78435 | 28.53 | 94.38 | 0.040 |

| Table S8. PCR Primer pairs for the most deleterious missense SNPs in UGT1A1/4 identified in this study. | | | | | | | | | |
| --- | --- | --- | --- | --- | --- | --- | --- | --- | --- |
| 1.UGT1A1- G308R - rs746111352 ( chr2:233767091 G to A) Primer pair | | | | | | | | | |
|  | **Sequence (5'->3')** | **Template strand** | **Length** | **Start** | **Stop** | **Tm** | **GC%** | **Self-complementarity** | **Self 3' complementarity** |
| Forward primer | TCTGGAGAACATGGAATTGTGG | **Plus** | **22** | **233767058** | **233767079** | **58.31** | **45.45** | **4** | **0** |
| Reverse primer | TGCCATAGCTTTCTTCTCTGG | **Minus** | **21** | **233767132** | **233767112** | **57.73** | **47.62** | **4** | **1** |
| Product length | **75** | | | | | | | | |
| 2.UGT1A1- P356T - rs767850186 (chr2:233767918 C to A) Primer pair | | | | | | | | | |
|  | **Sequence (5'->3')** | **Template strand** | **Length** | **Start** | **Stop** | **Tm** | **GC%** | **Self-complementarity** | **Self 3' complementarity** |
| Forward primer | CGAATCTTGCGAACAACACGA | **Plus** | **21** | **233767880** | **233767900** | **59.81** | **47.62** | **3** | **0** |
| Reverse primer | CCAATCCGCCCAACATACCA | **Minus** | **20** | **233767954** | **233767935** | **60.40** | **55** | **2** | **0** |
| Product length | **75** | | | | | | | | |
| 3.UGT1A1- G374S - rs1276913504 (chr2:233768255 G to A) Primer pair | | | | | | | | | |
|  | **Sequence (5'->3')** | **Template strand** | **Length** | **Start** | **Stop** | **Tm** | **GC%** | **Self-complementarity** | **Self 3' complementarity** |
| Forward primer | ATTTTGCATCTCAGGTCACCC | **Plus** | **21** | **233768206** | **233768226** | **58.55** | **47.62** | **5** | **3** |
| Reverse primer | TCATCACCATGGGAACGCC | **Minus** | **19** | **233768306** | **233768288** | **60.08** | **57.89** | **6** | **1** |
| Product length | **101** | | | | | | | | |
| 4.UGT1A4 - G309R - rs746111352 ( chr2:233767091 G to A) Primer pair | | | | | | | | | |
|  | **Sequence (5'->3')** | **Template strand** | **Length** | **Start** | **Stop** | **Tm** | **GC%** | **Self-complementarity** | **Self 3' complementarity** |
| Forward primer | TCTGGAGAACATGGAATTGTGG | **Plus** | **22** | **233767058** | **233767079** | **58.31** | **45.45** | **4** | **0** |
| Reverse primer | TGCCATAGCTTTCTTCTCTGG | **Minus** | **21** | **233767132** | **233767112** | **57.73** | **47.62** | **4** | **1** |
| Product length | **75** | | | | | | | | |
| 5.UGT1A4 - P357T - rs767850186 ( chr2:233767918 C to A) Primer pair | | | | | | | | | |
|  | **Sequence (5'->3')** | **Template strand** | **Length** | **Start** | **Stop** | **Tm** | **GC%** | **Self-complementarity** | **Self 3' complementarity** |
| Forward primer | CGAATCTTGCGAACAACACGA | **Plus** | **21** | **233767880** | **233767900** | **59.81** | **47.62** | **3** | **0** |
| Reverse primer | CCAATCCGCCCAACATACCA | **Minus** | **20** | **233767954** | **233767935** | **60.40** | **55** | **2** | **0** |
| Product length | **75** | | | | | | | | |
| 6.UGT1A4 - G375S - rs1276913504 ( chr2:233768255 G to A) Primer pair | | | | | | | | | |
|  | **Sequence (5'->3')** | **Template strand** | **Length** | **Start** | **Stop** | **Tm** | **GC%** | **Self-complementarity** | **Self 3' complementarity** |
| Forward primer | ATTTTGCATCTCAGGTCACCC | **Plus** | **21** | **233768206** | **233768226** | **58.55** | **47.62** | **5** | **3** |
| Reverse primer | ATCATCACCATGGGAACGCC | **Minus** | **20** | **233768307** | **233768288** | **60.47** | **55.00** | **6** | **1** |
| Product length | **102** | | | | | | | | |

| Table S9. Non-coding sequence variants in UGT1A1/4 and their RegulomeDB ranking | |
| --- | --- |
| Variant ID | **Ranking** |
| UGT1A1 | |
| rs113386420 | 4 |
| rs1040602725 | 4 |
| rs1365658904 | 4 |
| rs112008667 | 4 |
| rs752018052 | 4 |
| rs376515645 | 4 |
| rs773424672 | 5 |
| rs1287050195 | 5 |
| rs1042710 | 5 |
| rs1250920366 | 5 |
| rs1387922746 | 5 |
| rs760844779 | 5 |
| rs1299968482 | 5 |
| rs370727977 | 5 |
| rs774491304 | 5 |
| rs945548426 | 5 |
| rs1228021328 | 5 |
| rs1042595424 | 5 |
| rs906816673 | 5 |
| rs1313882407 | 5 |
| rs1003147991 | 5 |
| rs754114806 | 5 |
| rs34895241 | 5 |
| rs894885863 | 5 |
| rs1343648655 | 5 |
| rs182019761 | 5 |
| rs78684540 | 5 |
| rs1430291963 | 5 |
| rs1004907287 | 5 |
| rs1338074257 | 5 |
| rs1017104769 | 2b |
| rs1369161059 | 4 |
| rs542172440 | 4 |
| rs1314986383 | 4 |
| rs561946796 | 4 |
| rs1170923656 | 4 |
| rs1415303510 | 4 |
| rs769331263 | 4 |
| rs1471091659 | 4 |
| rs1307369412 | 4 |
| rs1029569602 | 4 |
| rs955306134 | 4 |
| rs1259415832 | 4 |
| rs1019873826 | 4 |
| rs1441521064 | 4 |
| rs200041554 | 2b |
| rs901299936 | 2b |
| rs997044425 | 2b |
| rs1353393417 | 4 |
| rs1028385252 | 4 |
| rs541532523 | 4 |
| rs910513508 | 4 |
| rs10929303 | 1f |
| rs989549467 | 4 |
| rs1450071408 | 4 |
| rs1358825451 | 4 |
| rs1332210568 | 4 |
| rs1402527560 | 2b |
| rs532329443 | 2b |
| rs1342292441 | 2b |
| rs61757316 | 2b |
| rs945545582 | 2b |
| rs762669023 | 4 |
| rs1472229025 | 4 |
| rs1271510565 | 4 |
| rs1314535454 | 4 |
| rs1021003584 | 4 |
| rs1237319377 | 4 |
| rs966644080 | 4 |
| rs759810185 | 4 |
| rs982401399 | 4 |
| rs906785821 | 4 |
| rs939644938 | 4 |
| rs927905587 | 4 |
| rs988153178 | 4 |
| rs1378630900 | 4 |
| rs1433214773 | 4 |
| rs1248675472 | 4 |
| rs554656848 | 4 |
| rs1320013324 | 4 |
| rs1042640 | 1f |
| rs1167184820 | 4 |
| rs1404550600 | 4 |
| rs112908387 | 4 |
| rs1169260027 | 4 |
| rs1449737327 | 4 |
| rs930433907 | 4 |
| rs1186146273 | 2b |
| rs1463887536 | 4 |
| rs1005644336 | 4 |
| rs1038101651 | 4 |
| rs569101199 | 4 |
| rs907498423 | 4 |
| rs751998224 | 4 |
| rs34942353 | 4 |
| rs1216509840 | 4 |
| rs1405131407 | 4 |
| rs944391861 | 4 |
| rs138626513 | 4 |
| rs8330 | 1f |
| rs61757317 | 4 |
| rs142810023 | 4 |
| rs1267960235 | 3a |
| rs1347868003 | 3a |
| rs1330670663 | 4 |
| rs997006332 | 4 |
| rs1049943141 | 4 |
| rs893890445 | 4 |
| rs1455189353 | 2b |
| rs1425331506 | 2b |
| rs1192252609 | 2b |
| rs1488458773 | 2b |
| rs1245298451 | 2b |
| rs1220355812 | 5 |
| rs1483468157 | 5 |
| rs1278315823 | 5 |
| rs1321886255 | 5 |
| rs1559421928 | 5 |
| rs1274189847 | 5 |
| rs1226545500 | 5 |
| rs1210765993 | 5 |
| rs886055800 | 5 |
| rs553436183 | 5 |
| rs1302116091 | 5 |
| rs1218531095 | 5 |
| rs768182403 | 5 |
| rs1365431114 | 5 |
| rs955192619 | 5 |
| rs1410214491 | 5 |
| rs1006776000 | 5 |
| rs1018620195 | 5 |
| rs1464666615 | 5 |
| rs1436243452 | 5 |
| rs964716810 | 5 |
| rs1191094075 | 5 |
| rs1174769439 | 5 |
| rs989910459 | 5 |
| rs570239690 | 5 |
| rs71539604 | 5 |
| rs915315951 | 5 |
| rs1473113676 | 5 |
| rs760814936 | 5 |
| rs1251603336 | 5 |
| rs1203251078 | 5 |
| rs1484380021 | 5 |
| rs1191731023 | 5 |
| rs967229603 | 5 |
| rs148128252 | 5 |
| rs1310299264 | 5 |
| rs1428279395 | 5 |
| rs868491556 | 5 |
| rs1468042895 | 5 |
| rs1290134629 | 5 |
| UGT1A4 | |
| rs545724404 | 4 |
| rs1216581751 | 4 |
| rs1346816253 | 4 |
| rs1318689875 | 4 |
| rs545172755 | 2b |
| rs1312767733 | 2b |
| rs372600165 | 4 |
| rs994719163 | 4 |
| rs1384235813 | 4 |
| rs1181429824 | 4 |
| rs374057137 | 4 |
| rs1347368179 | 2b |
| rs1216894118 | 2b |
| rs765403855 | 2b |
| rs767988524 | 2b |
| rs199517966 | 2b |
| rs372445273 | 2b |
| rs544842461 | 2b |
| rs911216236 | 2b |
| rs780688906 | 2b |
| rs745402322 | 2b |
| rs1294546267 | 2b |
| rs766693959 | 2b |
| rs375687360 | 2b |
| rs1190381622 | 2b |
| rs1241026493 | 2b |
| rs779656703 | 4 |
| rs748979755 | 4 |
| rs369738416 | 4 |
| rs747487357 | 4 |
| rs773424672 | 5 |
| rs1287050195 | 5 |
| rs1042710 | 5 |
| rs1250920366 | 5 |
| rs1387922746 | 5 |
| rs760844779 | 5 |
| rs1299968482 | 5 |
| rs370727977 | 5 |
| rs945548426 | 5 |
| rs774491304 | 5 |
| rs1228021328 | 5 |
| rs1042595424 | 5 |
| rs906816673 | 5 |
| rs1313882407 | 5 |
| rs1003147991 | 5 |
| rs754114806 | 5 |
| rs34895241 | 5 |
| rs894885863 | 5 |
| rs1343648655 | 5 |
| rs182019761 | 5 |
| rs78684540 | 5 |
| rs1430291963 | 5 |
| rs1004907287 | 5 |
| rs1338074257 | 5 |
| rs1017104769 | 2b |
| rs1369161059 | 4 |
| rs542172440 | 4 |
| rs1314986383 | 4 |
| rs561946796 | 4 |
| rs1415303510 | 4 |
| rs1170923656 | 4 |
| rs769331263 | 4 |
| rs1471091659 | 4 |
| rs1307369412 | 4 |
| rs1029569602 | 4 |
| rs955306134 | 4 |
| rs1259415832 | 4 |
| rs1019873826 | 4 |
| rs1441521064 | 4 |
| rs200041554 | 2b |
| rs901299936 | 2b |
| rs997044425 | 2b |
| rs1353393417 | 4 |
| rs1028385252 | 4 |
| rs541532523 | 4 |
| rs910513508 | 4 |
| rs10929303 | 1f |
| rs989549467 | 4 |
| rs1450071408 | 4 |
| rs1358825451 | 4 |
| rs1332210568 | 4 |
| rs1402527560 | 2b |
| rs532329443 | 2b |
| rs1342292441 | 2b |
| rs61757316 | 2b |
| rs945545582 | 2b |
| rs762669023 | 4 |
| rs1472229025 | 4 |
| rs1271510565 | 4 |
| rs1314535454 | 4 |
| rs1021003584 | 4 |
| rs1237319377 | 4 |
| rs966644080 | 4 |
| rs759810185 | 4 |
| rs982401399 | 4 |
| rs906785821 | 4 |
| rs939644938 | 4 |
| rs927905587 | 4 |
| rs1378630900 | 4 |
| rs988153178 | 4 |
| rs1433214773 | 4 |
| rs1248675472 | 4 |
| rs554656848 | 4 |
| rs1320013324 | 4 |
| rs1042640 | 1f |
| rs1167184820 | 4 |
| rs1404550600 | 4 |
| rs112908387 | 4 |
| rs1169260027 | 4 |
| rs1449737327 | 4 |
| rs930433907 | 4 |
| rs1186146273 | 2b |
| rs1463887536 | 4 |
| rs1005644336 | 4 |
| rs1038101651 | 4 |
| rs569101199 | 4 |
| rs907498423 | 4 |
| rs751998224 | 4 |
| rs34942353 | 4 |
| rs1216509840 | 4 |
| rs1405131407 | 4 |
| rs944391861 | 4 |
| rs138626513 | 4 |
| rs8330 | 1f |
| rs61757317 | 4 |
| rs142810023 | 4 |
| rs1267960235 | 3a |
| rs1347868003 | 3a |
| rs1330670663 | 4 |
| rs997006332 | 4 |
| rs1049943141 | 4 |
| rs893890445 | 4 |
| rs1455189353 | 2b |
| rs1425331506 | 2b |
| rs1192252609 | 2b |
| rs1488458773 | 2b |
| rs1245298451 | 2b |
| rs1220355812 | 5 |
| rs1483468157 | 5 |
| rs1278315823 | 5 |
| rs1321886255 | 5 |
| rs1559421928 | 5 |
| rs1274189847 | 5 |
| rs1226545500 | 5 |
| rs1210765993 | 5 |
| rs886055800 | 5 |
| rs553436183 | 5 |
| rs1302116091 | 5 |
| rs1218531095 | 5 |
| rs768182403 | 5 |
| rs1365431114 | 5 |
| rs955192619 | 5 |
| rs1410214491 | 5 |
| rs1006776000 | 5 |
| rs1018620195 | 5 |
| rs1464666615 | 5 |
| rs1436243452 | 5 |
| rs964716810 | 5 |
| rs1191094075 | 5 |
| rs1174769439 | 5 |
| rs989910459 | 5 |
| rs570239690 | 5 |
| rs71539604 | 5 |
| rs915315951 | 5 |
| rs1473113676 | 5 |
| rs760814936 | 5 |
| rs1251603336 | 5 |
| rs1203251078 | 5 |
| rs1484380021 | 5 |
| rs1191731023 | 5 |
| rs967229603 | 5 |
| rs148128252 | 5 |
| rs1310299264 | 5 |
| rs1428279395 | 5 |
| rs868491556 | 5 |
| rs1468042895 | 5 |
| rs1290134629 | 5 |

| Table S10. PolymiRTS results of non-coding variants in UGT1A1/4 | | | | |
| --- | --- | --- | --- | --- |
| **dbSNP ID** | **Consequence** | **miR ID** | **miRSite** | **Function Class** |
| rs1042640 | 3 prime UTR variant | hsa-miR-885-3p | tcccacCGCTGCC | O |
| rs112908387 | 3 prime UTR variant | hsa-miR-6085 | ggctGCCCCTAct | D |
|  |  | hsa-miR-6743-5p | ggcTGCCCCTAct | D |
|  |  | hsa-miR-6789-5p | ggctGCCCCTAct | D |
|  |  | hsa-miR-6813-5p | ggctGCCCCTAct | D |
| rs142810023 | 3 prime UTR variant | hsa-miR-4457 | caCCTTGTGtgtt | D |
|  |  | hsa-miR-4731-3p | cacCTTGTGTgtt | D |
|  |  | hsa-miR-4801 | cacCTTGTGTgtt | D |
|  |  | hsa-miR-624-3p | cACCTTGTgtgtt | D |
| rs148128252 | 3 prime UTR variant | hsa-miR-2115-3p | ttttTTCTGATgt | D |
|  |  | hsa-miR-3613-3p | ttTTTTTTGAtgt | C |
| rs182019761 | 3 prime UTR variant | hsa-miR-141-3p | CAGTGTTAaattc | D |
|  |  | hsa-miR-200a-3p | CAGTGTTAaattc | D |
|  |  | hsa-miR-302c-5p | cagTGTTAAAttc | D |
|  |  | hsa-miR-590-3p | cagtgTAAAATTc | C |
|  |  | hsa-miR-6757-3p | CAGTGTAaaattc | C |
| rs200041554 | 3 prime UTR variant | hsa-miR-3671 | agaTATTTGAAta | D |
|  |  | hsa-miR-4272 | agatatTTGAATA | D |
|  |  | hsa-miR-607 | agatATTTGAAta | D |
|  |  | hsa-miR-130a-5p | agatATGTGAAta | C |
|  |  | hsa-miR-23a-3p | agatATGTGAAta | C |
|  |  | hsa-miR-23b-3p | agatATGTGAAta | C |
|  |  | hsa-miR-23c | agatATGTGAAta | C |
| rs34895241 | 3 prime UTR variant | hsa-miR-3978 | tagtcaTTTCCAA | D |
|  |  | hsa-miR-6079 | tagtcaCTTCCAA | C |
| rs34942353 | 3 prime UTR variant | hsa-miR-591 | caataATGGTCAg | D |
|  |  | hsa-miR-6512-5p | caaTAATGGTcag | D |
| rs61757316 | 3 prime UTR variant | hsa-miR-3909 | AGAGGACgtgcag | D |
|  |  | hsa-miR-6852-3p | AGAGGACgtgcag | D |
| rs61757317 | 3 prime UTR variant | hsa-miR-8060 | CTTCATGgtgcc | O |
| rs71539604 | 3 prime UTR variant | hsa-miR-383-3p | tgaAGTGCTGggc | D |
| rs78684540 | 3 prime UTR variant | hsa-miR-3163 | attcATTTTATtc | D |
|  |  | hsa-miR-3646 | aTTCATTTtattc | D |
|  |  | hsa-miR-3662 | atTCATTTTAttc | D |
|  |  | hsa-miR-4426 | attCATCTTAttc | C |
|  |  | hsa-miR-4647 | attCATCTTAttc | C |
|  |  | hsa-miR-4662b | attCATCTTAttc | C |

| **Table S11. RegulomeDB webserver ranks and what the supported data for each rank.** | |
| --- | --- |
| **The RegulomeDB Rank** | **Related supporting Data** |
| **1a** | eQTL/caQTL, TF binding, matched TF motif, matched Footprint, chromatin accessibility peak |
| **1b** | eQTL/caQTL, TF binding, any motif, Footprint, chromatin accessibility peak |
| **1c** | eQTL/caQTL, TF binding, matched TF motif, chromatin accessibility peak |
| **1d** | eQTL/caQTL, TF binding, any motif, chromatin accessibility peak |
| **1e** | eQTL/caQTL, TF binding, matched TF motif |
| **1f** | eQTL/caQTL, TF binding or chromatin accessibility peak |
| **2a** | TF binding, matched TF motif, matched Footprint, chromatin accessibility peak |
| **2b** | TF binding, any motif, Footprint, chromatin accessibility peak |
| **2c** | TF binding, matched TF motif, chromatin accessibility peak |
| **3a** | TF binding, any motif, chromatin accessibility peak |
| **3b** | TF binding + matched TF motif |
| **4** | TF binding, chromatin accessibility peak |
| **5** | TF binding or chromatin accessibility peak |
| **6** | Motif hit |
| **7** | Other |

| Table S12. List of all bioinformatics tools used for determination of deleteriousness, stability, conservation and structural damage. | | | | |
| --- | --- | --- | --- | --- |
| Determination category | **Tool’s name** | **Tool’s prediction outcomes** | **URL** | **References** |
| Determination of deleteriousness | SIFT  (SIFT4G predictions) | Deleterious/Tolerated | https://sift.bii.a-star.edu.sg | Sim et al.[29] |
|  | PANTHER v 19.0 | Damaging/Benign | http://pantherdb.org/tools/csnpScoreForm.jsp | Tang et al.[36] |
|  | Polyphen-2 ( Polymorphism Phenotyping v2) | Probably damaging/Possibly damaging/Benign | http://genetics.bwh.harvard.edu/pph2/dbsearch.shtml | Adzhubei et al.[30] |
|  | E-SNPs&GO | Pathogenic/ Benign | https://esnpsandgo.biocomp.unibo.it/ | Manfredi et al.[35] |
|  | PHD-SNP | Disease/Neutral | https://snps.biofold.org/phd-snp/phd-snp.html | Capriotti et al.[32] |
|  | FATHMM v2.3 | Damaging/Tolerated | http://fathmm.biocompute.org.uk/ | Shihab et al.[37] |
|  | SNAP | Disease/Neutral | https://www.rostlab.org/services/snap/ | Bromberg et al.[33] |
|  | MutationAssessor | High/Medium/Low/Neutral | http://mutationassessor.org/r3/ | Frousios et al.[31] |
|  | Meta-SNP | Disease/Neutral | https://snps.biofold.org/meta-snp/ | Capriotti et al.[34] |
| Determination of stability upon mutation | MuPro | Decreased stability/ Increased stability | http://mupro.proteomics.ics.uci.edu/ | Khan et al.[39] |
|  | I-Mutant v.3.0 | Decreased stability/ Increased stability | https://folding.biofold.org/i-mutant/i-mutant2.0.html | Capriotti et al.[38] |
| Determination of conservation score | ConSurf | A conservation scale from 0 to 9 | http://consurf.tau.ac.il | Chorin et al.[40] |
| Identification of protein binding sites | COACH | The binding residues of the enzyme | https://zhanggroup.org/COACH/ | Yang et al[49] |
| Modelling of the SNPs | Swiss-Model | PDB files of the modelled SNPs | https://swissmodel.expasy.org/ | Waterhouse et al.[42] |
| Models’ validation | ERRAT | ERRAT scores help to evaluate the quality of the modelled structure to be examined further. | https://www.doe-mbi.ucla.edu/errat/ | Colovos & Yeates et al.[44] |
|  | PROSA | The PROSA z score reflects the overall quality of the model | https://prosa.services.came.sbg.ac.at/prosa.php | Wiederstein & Sippl et al.[45] |
|  | QMEANDisCo | Sequence identity, QMEAN, C-beta (Cβ), all atoms, solvation, torsion, GMQE and QMEANDisCo Global | https://swissmodel.expasy.org/qmean/ | Studer et al.[46]  Benkert et al.[47] |
|  | MolProbity | MolProbity score, Clash score, Ramachandran favoured and amachandran Outliers | http://molprobity.biochem.duke.edu/ | Chen et al.[48] |
| Docking | MOE | Scores of docking poses | https://www.chemcomp.com/Products.htm | Chemical Computing Group et al.[51] |
| Docking results 2D/3D visualization | BIOVIA Discovery studio | 2D and 3D picture representations of all drug-protein docking poses illustrating the ponds and their types | https://www.3ds.com/products/biovia/discovery-studio/ | Dassault Systèmes BIOVIA et al.[52] |
| Structural changes upon mutation | HOPE | Amino acid changes following mutation and the inflicted consequential structural damage. | https://www3.cmbi.umcn.nl/hope/input/ | Venselaar et al.[53] |
|  | DynaMut2 | Impact of point mutations on amino acid interactions. | https://biosig.lab.uq.edu.au/dynamut2/ | Rodrigues et al.[54] |
| Disrupting Protein's Physicochemical Parameters | Protparam | The tool facilitates the calculation of diverse physical and chemical parameters for a specified protein sequence. | https://web.expasy.org/protparam/ | Walker et al.[55] |
| Evaluation of non-coding SNPs | RegulomeDB | RegulomeDB score based on various high-throughput databases | https://regulomedb.org/regulome-search | Dong et al.[56] |
|  | PolymiRTS | PolymiRTS estimates SNPs located within miRNA target sites, which either disrupt or generate miRNA seed target sites. | https://compbio.uthsc.edu/miRSNP/ | Bhattacharya et al.[57] |

| **Table S13.** **Raw predictions for all retrieved UGT1A1 SNPS from all utilized in-silico tools** | | | | | | | | | | | |
| --- | --- | --- | --- | --- | --- | --- | --- | --- | --- | --- | --- |
| **Transcript** | **Variant ID** | **AA mutation** | **sift** | **Polyphen2** | **Mutation assessor** | **PhD-SNP** | **SNAP** | **Meta-SNP** | **E-SNPs&GO** | **Panther** | **FATHMM** |
| ENST00000305208.11 | rs2125981972 | A2T | tolerated - low confidence | possibly damaging | low | neutral | neutral | neutral | Benign | Benign | tolerated |
| ENST00000305208.11 | rs2125981972 | A2P | deleterious - low confidence | probably damaging | medium | neutral | neutral | neutral | Benign | Benign | tolerated |
| ENST00000305208.11 | rs2125981972 | A2S | tolerated - low confidence | possibly damaging | medium | neutral | neutral | neutral | Benign | Benign | tolerated |
| ENST00000305208.11 | rs1437444805 | A2G | deleterious - low confidence | possibly damaging | medium | neutral | neutral | neutral | Benign | Benign | tolerated |
| ENST00000305208.11 | rs1437444805 | A2V | tolerated - low confidence | possibly damaging | medium | neutral | neutral | neutral | Benign | Benign | tolerated |
| ENST00000305208.11 | rs1697387669 | V3M | tolerated - low confidence | benign | neutral | neutral | neutral | neutral | Benign | Benign | tolerated |
| ENST00000305208.11 | rs1697387669 | V3L | tolerated - low confidence | benign | neutral | neutral | neutral | neutral | Benign | Benign | tolerated |
| ENST00000305208.11 | rs74720349 | V3G | tolerated - low confidence | benign | low | neutral | neutral | neutral | Benign | Benign | tolerated |
| ENST00000305208.11 | rs2125982051 | E4K | tolerated - low confidence | possibly damaging | neutral | neutral | neutral | neutral | Benign | Benign | tolerated |
| ENST00000305208.11 | rs2125982051 | E4Q | tolerated - low confidence | possibly damaging | neutral | neutral | neutral | neutral | Benign | Benign | tolerated |
| ENST00000305208.11 | rs1697389087 | E4G | tolerated - low confidence | possibly damaging | neutral | neutral | neutral | neutral | Benign | Benign | tolerated |
| ENST00000305208.11 | rs1697389087 | E4V | tolerated - low confidence | possibly damaging | neutral | neutral | neutral | neutral | Benign | Benign | tolerated |
| ENST00000305208.11 | rs781590934 | E4D | tolerated - low confidence | possibly damaging | neutral | neutral | neutral | neutral | Benign | Benign | tolerated |
| ENST00000305208.11 | rs1271688129 | S5T | tolerated - low confidence | possibly damaging | low | neutral | neutral | neutral | Benign | Benign | tolerated |
| ENST00000305208.11 | rs1271688129 | S5A | tolerated - low confidence | benign | low | neutral | neutral | neutral | Benign | Benign | tolerated |
| ENST00000305208.11 | rs1697391714 | S5Y | tolerated - low confidence | possibly damaging | low | neutral | Disease | neutral | Benign | Benign | tolerated |
| ENST00000305208.11 | rs1697391714 | S5C | tolerated - low confidence | possibly damaging | low | neutral | neutral | neutral | Benign | Benign | tolerated |
| ENST00000305208.11 | rs2125982207 | Q6K | tolerated - low confidence | benign | low | neutral | neutral | neutral | Benign | Benign | tolerated |
| ENST00000305208.11 | rs2125982207 | Q6E | deleterious - low confidence | benign | low | neutral | Disease | neutral | Benign | Benign | tolerated |
| ENST00000305208.11 | rs2125982244 | Q6P | tolerated - low confidence | benign | neutral | neutral | Disease | neutral | Benign | Benign | tolerated |
| ENST00000305208.11 | rs2125982244 | Q6R | tolerated - low confidence | benign | neutral | neutral | neutral | neutral | Benign | Benign | tolerated |
| ENST00000305208.11 | rs2125982244 | Q6L | tolerated - low confidence | benign | low | neutral | neutral | neutral | Benign | Benign | tolerated |
| ENST00000305208.11 | rs745957787 | Q6H | tolerated - low confidence | benign | low | neutral | neutral | neutral | Benign | Benign | tolerated |
| ENST00000305208.11 | rs1006073064 | G7S | tolerated - low confidence | probably damaging | neutral | neutral | neutral | neutral | Benign | Benign | tolerated |
| ENST00000305208.11 | rs1006073064 | G7R | deleterious - low confidence | probably damaging | low | Disease | Disease | neutral | Benign | Benign | tolerated |
| ENST00000305208.11 | rs2125982302 | G7D | deleterious - low confidence | probably damaging | medium | Disease | Disease | Disease | Benign | Benign | tolerated |
| ENST00000305208.11 | rs2125982302 | G7A | tolerated - low confidence | probably damaging | low | neutral | neutral | neutral | Benign | Benign | tolerated |
| ENST00000305208.11 | rs749552053 | G8R | tolerated - low confidence | benign | neutral | neutral | Disease | neutral | Benign | Benign | tolerated |
| ENST00000305208.11 | rs768521253 | G8E | tolerated - low confidence | benign | neutral | Disease | Disease | neutral | Benign | Benign | tolerated |
| ENST00000305208.11 | rs768521253 | G8A | tolerated - low confidence | benign | neutral | neutral | neutral | neutral | Benign | Benign | tolerated |
| ENST00000305208.11 | rs768521253 | G8V | tolerated - low confidence | benign | neutral | neutral | neutral | neutral | Benign | Benign | tolerated |
| ENST00000305208.11 | rs370790922 | R9S | tolerated - low confidence | benign | neutral | neutral | neutral | neutral | Benign | Benign | tolerated |
| ENST00000305208.11 | rs370790922 | R9G | tolerated - low confidence | benign | low | neutral | neutral | neutral | Benign | Benign | tolerated |
| ENST00000305208.11 | rs370790922 | R9C | tolerated - low confidence | benign | neutral | Disease | Disease | neutral | Benign | Benign | tolerated |
| ENST00000305208.11 | rs386656374 | R9V | tolerated - low confidence | benign | neutral | neutral | Disease | neutral | Benign | Benign | tolerated |
| ENST00000305208.11 | rs761736540 | R9H | tolerated - low confidence | benign | neutral | neutral | Disease | neutral | Benign | Benign | tolerated |
| ENST00000305208.11 | rs761736540 | R9P | tolerated - low confidence | benign | neutral | Disease | Disease | Disease | Benign | Benign | tolerated |
| ENST00000305208.11 | rs761736540 | R9L | tolerated - low confidence | benign | neutral | Disease | neutral | neutral | Benign | Benign | tolerated |
| ENST00000305208.11 | rs2125982493 | P10A | tolerated - low confidence | benign | medium | neutral | neutral | neutral | Benign | Benign | tolerated |
| ENST00000305208.11 | rs2125982506 | P10Q | tolerated - low confidence | benign | low | neutral | neutral | neutral | Benign | Benign | tolerated |
| ENST00000305208.11 | rs2125982543 | L11V | tolerated - low confidence | possibly damaging | low | neutral | neutral | neutral | Benign | Benign | tolerated |
| ENST00000305208.11 | rs201984525 | L11H | tolerated - low confidence | probably damaging | medium | Disease | Disease | Disease | Benign | Benign | tolerated |
| ENST00000305208.11 | rs201984525 | L11P | tolerated - low confidence | possibly damaging | low | Disease | Disease | Disease | Benign | Benign | tolerated |
| ENST00000305208.11 | rs2125982570 | V12L | tolerated - low confidence | benign | neutral | neutral | neutral | neutral | Benign | Benign | tolerated |
| ENST00000305208.11 | rs2125982587 | V12D | tolerated - low confidence | possibly damaging | medium | neutral | Disease | Disease | Benign | Benign | tolerated |
| ENST00000305208.11 | rs2125982587 | V12G | tolerated - low confidence | possibly damaging | low | neutral | neutral | neutral | Benign | Benign | tolerated |
| ENST00000305208.11 | rs2125982623 | L13M | deleterious - low confidence | possibly damaging | medium | neutral | neutral | neutral | Benign | Benign | tolerated |
| ENST00000305208.11 | rs2125982623 | L13V | tolerated - low confidence | possibly damaging | low | neutral | neutral | neutral | Benign | Benign | tolerated |
| ENST00000305208.11 | rs2125982664 | G14R | tolerated - low confidence | benign | low | neutral | Disease | neutral | Pathogenic | Benign | tolerated |
| ENST00000305208.11 | rs1559406694 | G14D | tolerated - low confidence | benign | medium | neutral | neutral | neutral | Pathogenic | Benign | tolerated |
| ENST00000305208.11 | rs1559406694 | G14A | tolerated - low confidence | benign | low | neutral | neutral | neutral | Benign | Benign | tolerated |
| ENST00000305208.11 | rs2125982719 | L15V | tolerated - low confidence | possibly damaging | low | neutral | neutral | neutral | Benign | damaging | tolerated |
| ENST00000305208.11 | rs111033541 | L15P | deleterious - low confidence | possibly damaging | medium | Disease | Disease | Disease | Benign | damaging | tolerated |
| ENST00000305208.11 | rs111033541 | L15R | deleterious - low confidence | possibly damaging | medium | Disease | Disease | Disease | Pathogenic | damaging | tolerated |
| ENST00000305208.11 | rs1559406714 | L16M | deleterious - low confidence | possibly damaging | medium | neutral | neutral | neutral | Benign | damaging | tolerated |
| ENST00000305208.11 | rs1559406714 | L16V | tolerated - low confidence | possibly damaging | low | neutral | neutral | neutral | Benign | damaging | tolerated |
| ENST00000305208.11 | rs1575771125 | L16Q | deleterious - low confidence | possibly damaging | medium | neutral | Disease | neutral | Pathogenic | damaging | tolerated |
| ENST00000305208.11 | rs1472715638 | L17M | deleterious - low confidence | possibly damaging | medium | neutral | neutral | neutral | Benign | damaging | tolerated |
| ENST00000305208.11 | rs2125982853 | C18R | deleterious - low confidence | possibly damaging | medium | Disease | Disease | Disease | Pathogenic | Benign | tolerated |
| ENST00000305208.11 | rs2125982870 | C18Y | tolerated - low confidence | possibly damaging | medium | Disease | neutral | Disease | Benign | Benign | tolerated |
| ENST00000305208.11 | rs2125982885 | C18W | tolerated - low confidence | possibly damaging | medium | Disease | Disease | Disease | Benign | Benign | tolerated |
| ENST00000305208.11 | rs2125982896 | V19M | tolerated - low confidence | possibly damaging | low | neutral | neutral | neutral | Benign | Benign | tolerated |
| ENST00000305208.11 | rs2125982896 | V19L | tolerated - low confidence | benign | neutral | neutral | neutral | neutral | Benign | Benign | tolerated |
| ENST00000305208.11 | rs2125982923 | V19E | tolerated - low confidence | possibly damaging | medium | Disease | Disease | Disease | Pathogenic | Benign | tolerated |
| ENST00000305208.11 | rs2125982923 | V19G | tolerated - low confidence | possibly damaging | low | neutral | neutral | neutral | Benign | Benign | tolerated |
| ENST00000305208.11 | rs2125982956 | L20M | tolerated - low confidence | possibly damaging | medium | neutral | neutral | neutral | Benign | Benign | tolerated |
| ENST00000305208.11 | rs2125982956 | L20V | tolerated - low confidence | possibly damaging | low | neutral | neutral | neutral | Benign | Benign | tolerated |
| ENST00000305208.11 | rs759212811 | G21S | tolerated - low confidence | benign | neutral | neutral | neutral | neutral | Benign | Benign | tolerated |
| ENST00000305208.11 | rs1697415689 | V24M | tolerated - low confidence | benign | low | neutral | neutral | neutral | Benign | Benign | tolerated |
| ENST00000305208.11 | rs751894919 | S25T | tolerated - low confidence | benign | low | neutral | neutral | neutral | Benign | Benign | tolerated |
| ENST00000305208.11 | rs1400895192 | H26N | tolerated - low confidence | benign | low | neutral | neutral | neutral | Benign | Benign | tolerated |
| ENST00000305208.11 | rs1697418455 | H26R | tolerated - low confidence | benign | low | neutral | neutral | neutral | Benign | Benign | tolerated |
| ENST00000305208.11 | rs1447031389 | A27T | deleterious - low confidence | benign | low | neutral | neutral | Disease | Pathogenic | Benign | tolerated |
| ENST00000305208.11 | rs1337235315 | G28A | deleterious - low confidence | possibly damaging | low | neutral | neutral | Disease | Benign | damaging | tolerated |
| ENST00000305208.11 | rs1032753915 | I30L | tolerated - low confidence | benign | neutral | neutral | neutral | neutral | Pathogenic | Benign | tolerated |
| ENST00000305208.11 | rs375204962 | I30T | deleterious - low confidence | benign | medium | neutral | neutral | Disease | Pathogenic | Benign | tolerated |
| ENST00000305208.11 | rs1697424081 | I30M | deleterious - low confidence | benign | low | neutral | neutral | Disease | Pathogenic | Benign | tolerated |
| ENST00000305208.11 | rs1697426202 | L32W | deleterious - low confidence | probably damaging | medium | Disease | neutral | Disease | Pathogenic | Benign | tolerated |
| ENST00000305208.11 | rs1029837369 | L32F | deleterious - low confidence | possibly damaging | medium | Disease | neutral | neutral | Pathogenic | Benign | tolerated |
| ENST00000305208.11 | rs368348566 | I33N | deleterious - low confidence | possibly damaging | low | neutral | neutral | Disease | Pathogenic | Benign | tolerated |
| ENST00000305208.11 | rs368348566 | I33T | deleterious - low confidence | benign | low | neutral | neutral | neutral | Pathogenic | Benign | tolerated |
| ENST00000305208.11 | rs756175438 | P34S | deleterious - low confidence | probably damaging | medium | Disease | Disease | Disease | Pathogenic | damaging | tolerated |
| ENST00000305208.11 | rs1697429657 | D36N | deleterious - low confidence | probably damaging | high | Disease | neutral | Disease | Pathogenic | damaging | tolerated |
| ENST00000305208.11 | rs1697429657 | D36H | deleterious - low confidence | probably damaging | high | neutral | Disease | Disease | Benign | damaging | tolerated |
| ENST00000305208.11 | rs1244863017 | D36A | deleterious - low confidence | probably damaging | medium | Disease | Disease | Disease | Pathogenic | damaging | tolerated |
| ENST00000305208.11 | rs1244863017 | D36G | deleterious - low confidence | possibly damaging | medium | neutral | Disease | Disease | Pathogenic | damaging | tolerated |
| ENST00000305208.11 | rs1244863017 | D36V | deleterious - low confidence | probably damaging | high | Disease | Disease | Disease | Pathogenic | damaging | tolerated |
| ENST00000305208.11 | rs1697431093 | D36E | tolerated - low confidence | benign | neutral | neutral | neutral | Disease | Pathogenic | damaging | tolerated |
| ENST00000305208.11 | rs1697431780 | G37S | deleterious - low confidence | probably damaging | medium | Disease | Disease | Disease | Pathogenic | damaging | tolerated |
| ENST00000305208.11 | rs780016114 | G37D | deleterious - low confidence | probably damaging | high | Disease | Disease | Disease | Pathogenic | damaging | tolerated |
| ENST00000305208.11 | rs780016114 | G37A | deleterious - low confidence | probably damaging | medium | Disease | neutral | Disease | Pathogenic | damaging | tolerated |
| ENST00000305208.11 | rs780016114 | G37V | deleterious - low confidence | probably damaging | high | Disease | neutral | Disease | Pathogenic | damaging | tolerated |
| ENST00000305208.11 | rs2125983475 | S38N | deleterious - low confidence | probably damaging | high | Disease | Disease | Disease | Pathogenic | damaging | tolerated |
| ENST00000305208.11 | rs2125983475 | S38T | deleterious - low confidence | probably damaging | high | Disease | Disease | Disease | Pathogenic | damaging | tolerated |
| ENST00000305208.11 | rs2125983475 | S38I | deleterious - low confidence | probably damaging | high | Disease | Disease | Disease | Pathogenic | damaging | tolerated |
| ENST00000305208.11 | rs2125983506 | S38R | deleterious - low confidence | probably damaging | high | Disease | Disease | Disease | Pathogenic | damaging | tolerated |
| ENST00000305208.11 | rs72551339 | H39N | deleterious - low confidence | probably damaging | high | Disease | Disease | Disease | Pathogenic | damaging | tolerated |
| ENST00000305208.11 | rs72551339 | H39D | deleterious - low confidence | probably damaging | high | Disease | Disease | Disease | Pathogenic | damaging | tolerated |
| ENST00000305208.11 | rs72551339 | H39Y | deleterious - low confidence | probably damaging | high | Disease | Disease | Disease | Pathogenic | damaging | tolerated |
| ENST00000305208.11 | rs2125983562 | H39P | tolerated - low confidence | possibly damaging | medium | Disease | Disease | Disease | Pathogenic | damaging | tolerated |
| ENST00000305208.11 | rs2125983562 | H39L | deleterious - low confidence | probably damaging | high | Disease | Disease | Disease | Pathogenic | damaging | tolerated |
| ENST00000305208.11 | rs1211296854 | H39Q | deleterious - low confidence | possibly damaging | high | Disease | Disease | Disease | Pathogenic | damaging | tolerated |
| ENST00000305208.11 | rs1183811071 | W40R | deleterious - low confidence | probably damaging | high | Disease | Disease | Disease | Pathogenic | damaging | tolerated |
| ENST00000305208.11 | rs2125983610 | W40L | deleterious - low confidence | probably damaging | medium | Disease | neutral | Disease | Pathogenic | damaging | tolerated |
| ENST00000305208.11 | rs2125983643 | L41M | deleterious - low confidence | possibly damaging | medium | neutral | neutral | neutral | Benign | damaging | tolerated |
| ENST00000305208.11 | rs1575771994 | S42G | deleterious - low confidence | benign | medium | neutral | neutral | Disease | Pathogenic | damaging | tolerated |
| ENST00000305208.11 | rs1575771994 | S42C | deleterious - low confidence | probably damaging | high | Disease | neutral | Disease | Pathogenic | damaging | tolerated |
| ENST00000305208.11 | rs533404227 | S42N | deleterious - low confidence | possibly damaging | low | neutral | neutral | neutral | Benign | damaging | tolerated |
| ENST00000305208.11 | rs533404227 | S42T | tolerated - low confidence | benign | medium | neutral | neutral | Disease | Pathogenic | damaging | tolerated |
| ENST00000305208.11 | rs2125983699 | S42R | deleterious - low confidence | probably damaging | high | Disease | neutral | Disease | Pathogenic | damaging | tolerated |
| ENST00000305208.11 | rs2125983714 | M43L | tolerated - low confidence | benign | low | neutral | neutral | Disease | Benign | damaging | tolerated |
| ENST00000305208.11 | rs2125983730 | M43K | deleterious - low confidence | possibly damaging | high | Disease | Disease | Disease | Pathogenic | damaging | tolerated |
| ENST00000305208.11 | rs1575772039 | M43I | deleterious - low confidence | benign | medium | neutral | neutral | Disease | Benign | damaging | tolerated |
| ENST00000305208.11 | rs2125983770 | L44I | deleterious - low confidence | benign | low | neutral | neutral | Disease | Benign | Benign | tolerated |
| ENST00000305208.11 | rs2125983770 | L44V | tolerated - low confidence | benign | neutral | neutral | neutral | Disease | Benign | Benign | tolerated |
| ENST00000305208.11 | rs2125983770 | L44F | deleterious - low confidence | benign | neutral | neutral | neutral | Disease | Benign | Benign | tolerated |
| ENST00000305208.11 | rs1178608845 | L44H | tolerated - low confidence | benign | neutral | neutral | neutral | Disease | Benign | Benign | tolerated |
| ENST00000305208.11 | rs1697440373 | G45R | tolerated - low confidence | benign | low | neutral | neutral | Disease | Pathogenic | Benign | tolerated |
| ENST00000305208.11 | rs1697440373 | G45W | deleterious - low confidence | possibly damaging | medium | Disease | neutral | Disease | Pathogenic | Benign | tolerated |
| ENST00000305208.11 | rs2125983841 | G45E | tolerated - low confidence | benign | neutral | neutral | neutral | Disease | Pathogenic | Benign | tolerated |
| ENST00000305208.11 | rs2125983841 | G45V | tolerated - low confidence | benign | neutral | neutral | neutral | Disease | Pathogenic | Benign | tolerated |
| ENST00000305208.11 | rs886044683 | A46D | deleterious - low confidence | benign | low | Disease | Disease | Disease | Benign | Benign | tolerated |
| ENST00000305208.11 | rs886044683 | A46G | deleterious - low confidence | benign | low | neutral | neutral | Disease | Benign | Benign | tolerated |
| ENST00000305208.11 | rs886044683 | A46V | tolerated - low confidence | benign | neutral | neutral | neutral | neutral | Benign | Benign | tolerated |
| ENST00000305208.11 | rs2125983914 | I47L | tolerated - low confidence | benign | neutral | neutral | neutral | neutral | Benign | Benign | tolerated |
| ENST00000305208.11 | rs2125983914 | I47F | deleterious - low confidence | benign | medium | neutral | neutral | Disease | Benign | Benign | tolerated |
| ENST00000305208.11 | rs747942373 | Q48E | tolerated - low confidence | benign | neutral | neutral | neutral | neutral | Benign | Benign | tolerated |
| ENST00000305208.11 | rs2125983968 | Q48H | deleterious - low confidence | possibly damaging | medium | neutral | neutral | Disease | Benign | Benign | tolerated |
| ENST00000305208.11 | rs587776765 | Q49K | tolerated - low confidence | benign | neutral | neutral | neutral | Disease | Benign | Benign | tolerated |
| ENST00000305208.11 | rs587776765 | Q49E | tolerated - low confidence | benign | neutral | neutral | neutral | neutral | Benign | Benign | tolerated |
| ENST00000305208.11 | rs771774728 | Q49R | tolerated - low confidence | benign | low | Disease | neutral | neutral | Benign | Benign | tolerated |
| ENST00000305208.11 | rs771774728 | Q49L | tolerated - low confidence | benign | low | neutral | neutral | Disease | Benign | Benign | tolerated |
| ENST00000305208.11 | rs2125984030 | L50M | deleterious - low confidence | possibly damaging | medium | Disease | neutral | Disease | Benign | damaging | damaging |
| ENST00000305208.11 | rs2125984030 | L50V | deleterious - low confidence | possibly damaging | high | Disease | neutral | Disease | Benign | damaging | damaging |
| ENST00000305208.11 | rs2125984045 | L50R | deleterious - low confidence | probably damaging | high | Disease | Disease | Disease | Pathogenic | damaging | damaging |
| ENST00000305208.11 | rs2125984063 | Q51K | tolerated - low confidence | benign | medium | neutral | neutral | Disease | Benign | Benign | tolerated |
| ENST00000305208.11 | rs2125984063 | Q51E | tolerated - low confidence | benign | medium | neutral | neutral | Disease | Benign | Benign | tolerated |
| ENST00000305208.11 | rs1697446829 | Q51P | tolerated - low confidence | benign | medium | Disease | neutral | Disease | Pathogenic | Benign | tolerated |
| ENST00000305208.11 | rs1697446829 | Q51R | tolerated - low confidence | benign | low | neutral | neutral | Disease | Benign | Benign | tolerated |
| ENST00000305208.11 | rs1697446829 | Q51L | tolerated - low confidence | benign | low | neutral | neutral | Disease | Benign | Benign | tolerated |
| ENST00000305208.11 | rs1697447579 | Q51H | tolerated - low confidence | benign | neutral | neutral | neutral | neutral | Benign | Benign | tolerated |
| ENST00000305208.11 | rs2125984132 | Q52E | tolerated - low confidence | benign | low | neutral | neutral | Disease | Benign | damaging | tolerated |
| ENST00000305208.11 | rs773136953 | Q52H | tolerated - low confidence | benign | medium | neutral | neutral | Disease | Benign | damaging | tolerated |
| ENST00000305208.11 | rs2125984157 | R53G | deleterious - low confidence | probably damaging | high | Disease | Disease | Disease | Pathogenic | Benign | tolerated |
| ENST00000305208.11 | rs2125984169 | R53K | tolerated - low confidence | benign | low | neutral | neutral | Disease | Benign | Benign | tolerated |
| ENST00000305208.11 | rs2125984169 | R53M | deleterious - low confidence | probably damaging | high | Disease | Disease | Disease | Pathogenic | Benign | tolerated |
| ENST00000305208.11 | rs149071335 | R53S | deleterious - low confidence | possibly damaging | high | Disease | Disease | Disease | Benign | Benign | tolerated |
| ENST00000305208.11 | rs2125984206 | G54R | deleterious - low confidence | probably damaging | high | Disease | Disease | Disease | Pathogenic | damaging | tolerated |
| ENST00000305208.11 | rs770426284 | G54E | deleterious - low confidence | probably damaging | high | Disease | Disease | Disease | Benign | damaging | tolerated |
| ENST00000305208.11 | rs770426284 | G54A | deleterious - low confidence | probably damaging | high | Disease | Disease | Disease | Benign | damaging | tolerated |
| ENST00000305208.11 | rs770426284 | G54V | deleterious - low confidence | probably damaging | high | Disease | Disease | Disease | Pathogenic | damaging | tolerated |
| ENST00000305208.11 | rs1218272105 | H55D | deleterious - low confidence | probably damaging | high | Disease | Disease | Disease | Pathogenic | damaging | tolerated |
| ENST00000305208.11 | rs1218272105 | H55Y | deleterious - low confidence | probably damaging | high | Disease | neutral | Disease | Pathogenic | damaging | tolerated |
| ENST00000305208.11 | rs1697451536 | H55Q | deleterious - low confidence | probably damaging | high | Disease | Disease | Disease | Pathogenic | damaging | tolerated |
| ENST00000305208.11 | rs1035578503 | E56Q | tolerated - low confidence | benign | neutral | neutral | neutral | neutral | Benign | Benign | tolerated |
| ENST00000305208.11 | rs140365717 | E56A | deleterious - low confidence | benign | medium | neutral | neutral | Disease | Benign | Benign | tolerated |
| ENST00000305208.11 | rs140365717 | E56G | deleterious - low confidence | possibly damaging | high | neutral | neutral | neutral | Benign | Benign | tolerated |
| ENST00000305208.11 | rs2125984333 | E56D | tolerated - low confidence | benign | low | neutral | neutral | Disease | Benign | Benign | tolerated |
| ENST00000305208.11 | rs1341101614 | I57L | tolerated - low confidence | benign | low | neutral | neutral | Disease | Benign | Benign | tolerated |
| ENST00000305208.11 | rs2125984359 | I57K | deleterious - low confidence | possibly damaging | medium | Disease | neutral | Disease | Pathogenic | Benign | tolerated |
| ENST00000305208.11 | rs1336419159 | I57M | deleterious - low confidence | benign | medium | neutral | neutral | Disease | Benign | Benign | tolerated |
| ENST00000305208.11 | rs2125984399 | V58L | deleterious - low confidence | benign | medium | Disease | neutral | Disease | Benign | damaging | tolerated |
| ENST00000305208.11 | rs2125984411 | V58D | deleterious - low confidence | possibly damaging | medium | Disease | neutral | Disease | Benign | damaging | tolerated |
| ENST00000305208.11 | rs2125984411 | V58A | deleterious - low confidence | possibly damaging | medium | neutral | neutral | Disease | Benign | damaging | tolerated |
| ENST00000305208.11 | rs1297484811 | V59I | deleterious - low confidence | possibly damaging | medium | neutral | neutral | Disease | Benign | damaging | tolerated |
| ENST00000305208.11 | rs1297484811 | V59L | deleterious - low confidence | benign | medium | Disease | neutral | Disease | Benign | damaging | tolerated |
| ENST00000305208.11 | rs1297484811 | V59F | deleterious - low confidence | probably damaging | medium | Disease | neutral | Disease | Pathogenic | damaging | tolerated |
| ENST00000305208.11 | rs2125984475 | L60V | tolerated - low confidence | benign | neutral | neutral | neutral | neutral | Benign | Benign | tolerated |
| ENST00000305208.11 | rs370892808 | L60Q | deleterious - low confidence | probably damaging | high | Disease | neutral | Disease | Pathogenic | Benign | tolerated |
| ENST00000305208.11 | rs370892808 | L60P | deleterious - low confidence | probably damaging | high | Disease | Disease | Disease | Pathogenic | Benign | tolerated |
| ENST00000305208.11 | rs2125984523 | A61T | tolerated - low confidence | benign | low | neutral | neutral | neutral | Benign | damaging | tolerated |
| ENST00000305208.11 | rs2125984523 | A61P | deleterious - low confidence | possibly damaging | medium | Disease | neutral | Disease | Pathogenic | damaging | tolerated |
| ENST00000305208.11 | rs1273237448 | A61E | deleterious - low confidence | possibly damaging | medium | Disease | neutral | Disease | Pathogenic | damaging | tolerated |
| ENST00000305208.11 | rs1273237448 | A61G | deleterious - low confidence | benign | medium | neutral | neutral | Disease | Benign | damaging | tolerated |
| ENST00000305208.11 | rs1273237448 | A61V | tolerated - low confidence | benign | low | neutral | neutral | neutral | Benign | damaging | tolerated |
| ENST00000305208.11 | rs1697459307 | P62S | deleterious - low confidence | possibly damaging | low | neutral | neutral | Disease | Benign | damaging | tolerated |
| ENST00000305208.11 | rs1344623676 | P62R | deleterious - low confidence | probably damaging | high | Disease | Disease | Disease | Pathogenic | damaging | tolerated |
| ENST00000305208.11 | rs1344623676 | P62L | deleterious - low confidence | possibly damaging | medium | Disease | neutral | Disease | Pathogenic | damaging | tolerated |
| ENST00000305208.11 | rs2125984618 | D63N | deleterious - low confidence | benign | low | neutral | neutral | Disease | Benign | Benign | tolerated |
| ENST00000305208.11 | rs2125984618 | D63H | deleterious - low confidence | benign | low | neutral | neutral | Disease | Benign | Benign | tolerated |
| ENST00000305208.11 | rs2125984618 | D63Y | deleterious - low confidence | possibly damaging | low | Disease | Disease | Disease | Pathogenic | Benign | tolerated |
| ENST00000305208.11 | rs2125984650 | D63A | deleterious - low confidence | benign | neutral | neutral | neutral | Disease | Benign | Benign | tolerated |
| ENST00000305208.11 | rs2125984650 | D63G | deleterious - low confidence | benign | low | Disease | neutral | Disease | Benign | Benign | tolerated |
| ENST00000305208.11 | rs2125984650 | D63V | deleterious - low confidence | benign | low | Disease | neutral | Disease | Pathogenic | Benign | tolerated |
| ENST00000305208.11 | rs191471887 | D63E | tolerated - low confidence | benign | neutral | neutral | neutral | neutral | Benign | Benign | tolerated |
| ENST00000305208.11 | rs775184773 | A64T | tolerated - low confidence | benign | low | neutral | neutral | neutral | Benign | Benign | tolerated |
| ENST00000305208.11 | rs775184773 | A64P | deleterious - low confidence | possibly damaging | medium | Disease | neutral | neutral | Benign | Benign | tolerated |
| ENST00000305208.11 | rs775184773 | A64S | tolerated - low confidence | benign | low | neutral | neutral | Disease | Benign | Benign | tolerated |
| ENST00000305208.11 | rs2125984695 | A64G | deleterious - low confidence | benign | low | neutral | neutral | neutral | Benign | Benign | tolerated |
| ENST00000305208.11 | rs2125984695 | A64V | tolerated - low confidence | benign | neutral | neutral | neutral | neutral | Benign | Benign | tolerated |
| ENST00000305208.11 | rs2125984721 | S65P | deleterious - low confidence | probably damaging | medium | Disease | neutral | Disease | Pathogenic | Benign | tolerated |
| ENST00000305208.11 | rs762109713 | S65W | deleterious - low confidence | probably damaging | high | Disease | Disease | Disease | Pathogenic | Benign | tolerated |
| ENST00000305208.11 | rs762109713 | S65L | deleterious - low confidence | possibly damaging | medium | Disease | neutral | Disease | Benign | Benign | tolerated |
| ENST00000305208.11 | rs750890851 | L66M | tolerated - low confidence | benign | neutral | neutral | neutral | neutral | Benign | Benign | tolerated |
| ENST00000305208.11 | rs750890851 | L66V | tolerated - low confidence | benign | neutral | neutral | neutral | neutral | Benign | Benign | tolerated |
| ENST00000305208.11 | rs2125984803 | L66F | deleterious - low confidence | benign | low | neutral | neutral | Disease | Benign | Benign | tolerated |
| ENST00000305208.11 | rs2125984836 | Y67N | tolerated - low confidence | benign | low | Disease | neutral | neutral | Benign | Benign | tolerated |
| ENST00000305208.11 | rs2125984836 | Y67H | tolerated - low confidence | benign | neutral | neutral | neutral | Disease | Benign | Benign | tolerated |
| ENST00000305208.11 | rs2125984836 | Y67D | deleterious - low confidence | benign | low | Disease | neutral | Disease | Benign | Benign | tolerated |
| ENST00000305208.11 | rs2125984867 | Y67S | tolerated - low confidence | benign | low | neutral | neutral | Disease | Benign | Benign | tolerated |
| ENST00000305208.11 | rs2125984867 | Y67C | deleterious - low confidence | possibly damaging | medium | Disease | neutral | Disease | Pathogenic | Benign | tolerated |
| ENST00000305208.11 | rs2125984867 | Y67F | tolerated - low confidence | benign | neutral | neutral | neutral | neutral | Benign | Benign | tolerated |
| ENST00000305208.11 | rs756552149 | I68L | tolerated - low confidence | benign | medium | neutral | neutral | Disease | Benign | damaging | tolerated |
| ENST00000305208.11 | rs756552149 | I68V | tolerated - low confidence | benign | low | neutral | neutral | neutral | Benign | damaging | tolerated |
| ENST00000305208.11 | rs756552149 | I68F | deleterious - low confidence | benign | medium | Disease | neutral | Disease | Benign | damaging | tolerated |
| ENST00000305208.11 | rs2125984919 | I68M | tolerated - low confidence | benign | medium | neutral | neutral | Disease | Benign | damaging | tolerated |
| ENST00000305208.11 | rs2125984938 | R69K | tolerated - low confidence | benign | neutral | neutral | neutral | neutral | Benign | Benign | tolerated |
| ENST00000305208.11 | rs2125984938 | R69T | deleterious - low confidence | benign | neutral | neutral | neutral | Disease | Benign | Benign | tolerated |
| ENST00000305208.11 | rs2125984938 | R69I | deleterious - low confidence | benign | low | neutral | neutral | Disease | Benign | Benign | tolerated |
| ENST00000305208.11 | rs2125984967 | D70N | tolerated - low confidence | benign | neutral | neutral | neutral | Disease | Benign | Benign | tolerated |
| ENST00000305208.11 | rs2125984967 | D70H | deleterious - low confidence | benign | neutral | neutral | neutral | Disease | Benign | Benign | tolerated |
| ENST00000305208.11 | rs766949747 | D70G | tolerated - low confidence | benign | neutral | Disease | neutral | Disease | Benign | Benign | tolerated |
| ENST00000305208.11 | rs766949747 | D70V | tolerated - low confidence | benign | neutral | Disease | neutral | Disease | Benign | Benign | tolerated |
| ENST00000305208.11 | rs1553620689 | D70E | tolerated - low confidence | benign | neutral | neutral | neutral | neutral | Benign | Benign | tolerated |
| ENST00000305208.11 | rs4148323 | G71R | deleterious - low confidence | possibly damaging | low | Disease | Disease | Disease | Benign | Benign | tolerated |
| ENST00000305208.11 | rs937258833 | G71E | tolerated - low confidence | benign | neutral | neutral | Disease | Disease | Benign | Benign | tolerated |
| ENST00000305208.11 | rs937258833 | G71A | tolerated - low confidence | benign | neutral | neutral | neutral | neutral | Benign | Benign | tolerated |
| ENST00000305208.11 | rs2125985080 | A72T | tolerated - low confidence | benign | low | neutral | neutral | Disease | Benign | Benign | tolerated |
| ENST00000305208.11 | rs2125985080 | A72P | tolerated - low confidence | benign | low | neutral | neutral | Disease | Benign | Benign | tolerated |
| ENST00000305208.11 | rs534361076 | A72G | tolerated - low confidence | benign | low | neutral | neutral | Disease | Benign | Benign | tolerated |
| ENST00000305208.11 | rs534361076 | A72V | tolerated - low confidence | benign | medium | neutral | neutral | Disease | Benign | Benign | tolerated |
| ENST00000305208.11 | rs2125985118 | F73I | tolerated - low confidence | benign | medium | neutral | neutral | Disease | Benign | damaging | tolerated |
| ENST00000305208.11 | rs2125985118 | F73L | tolerated - low confidence | benign | medium | neutral | neutral | Disease | Benign | damaging | tolerated |
| ENST00000305208.11 | rs2125985118 | F73V | tolerated - low confidence | benign | medium | neutral | neutral | Disease | Benign | damaging | tolerated |
| ENST00000305208.11 | rs1199756469 | Y74N | deleterious - low confidence | probably damaging | medium | Disease | neutral | Disease | Pathogenic | damaging | tolerated |
| ENST00000305208.11 | rs1199756469 | Y74H | deleterious - low confidence | probably damaging | medium | Disease | neutral | Disease | Pathogenic | damaging | tolerated |
| ENST00000305208.11 | rs983416663 | Y74S | deleterious - low confidence | possibly damaging | medium | Disease | neutral | neutral | Pathogenic | damaging | tolerated |
| ENST00000305208.11 | rs983416663 | Y74C | tolerated - low confidence | benign | medium | Disease | neutral | Disease | Benign | damaging | tolerated |
| ENST00000305208.11 | rs983416663 | Y74F | tolerated - low confidence | benign | neutral | neutral | neutral | neutral | Benign | damaging | tolerated |
| ENST00000305208.11 | rs2125985192 | T75S | tolerated - low confidence | benign | low | neutral | neutral | neutral | Benign | damaging | tolerated |
| ENST00000305208.11 | rs2125985199 | T75N | deleterious - low confidence | benign | medium | Disease | neutral | Disease | Benign | damaging | tolerated |
| ENST00000305208.11 | rs2125985199 | T75S | tolerated - low confidence | benign | low | neutral | neutral | Disease | Benign | damaging | tolerated |
| ENST00000305208.11 | rs2125985199 | T75I | tolerated - low confidence | benign | medium | Disease | neutral | Disease | Benign | damaging | tolerated |
| ENST00000305208.11 | rs2125985224 | L76M | tolerated - low confidence | benign | low | neutral | neutral | neutral | Benign | Benign | tolerated |
| ENST00000305208.11 | rs944619304 | L76S | deleterious - low confidence | benign | medium | neutral | neutral | Disease | Benign | Benign | tolerated |
| ENST00000305208.11 | rs1697473118 | L76F | deleterious - low confidence | benign | neutral | neutral | neutral | Disease | Benign | Benign | tolerated |
| ENST00000305208.11 | rs2125985278 | K77M | deleterious - low confidence | possibly damaging | medium | neutral | neutral | Disease | Benign | Benign | tolerated |
| ENST00000305208.11 | rs2125985288 | K77N | deleterious - low confidence | possibly damaging | medium | Disease | neutral | Disease | Benign | Benign | tolerated |
| ENST00000305208.11 | rs1575773178 | T78P | deleterious - low confidence | benign | medium | Disease | neutral | Disease | Benign | damaging | tolerated |
| ENST00000305208.11 | rs1575773178 | T78A | deleterious - low confidence | benign | medium | neutral | neutral | Disease | Benign | damaging | tolerated |
| ENST00000305208.11 | rs1575773178 | T78S | tolerated - low confidence | benign | low | neutral | neutral | Disease | Benign | damaging | tolerated |
| ENST00000305208.11 | rs779218106 | T78K | tolerated - low confidence | benign | low | Disease | neutral | neutral | Benign | damaging | tolerated |
| ENST00000305208.11 | rs779218106 | T78R | tolerated - low confidence | benign | low | Disease | neutral | Disease | Benign | damaging | tolerated |
| ENST00000305208.11 | rs779218106 | T78M | tolerated - low confidence | benign | medium | neutral | neutral | Disease | Benign | damaging | tolerated |
| ENST00000305208.11 | rs2125985371 | Y79N | deleterious - low confidence | probably damaging | medium | Disease | Disease | Disease | Pathogenic | damaging | tolerated |
| ENST00000305208.11 | rs2125985371 | Y79H | deleterious - low confidence | benign | medium | Disease | neutral | Disease | Pathogenic | damaging | tolerated |
| ENST00000305208.11 | rs1697475352 | Y79S | deleterious - low confidence | probably damaging | medium | Disease | Disease | Disease | Benign | damaging | tolerated |
| ENST00000305208.11 | rs1697475352 | Y79C | deleterious - low confidence | probably damaging | medium | Disease | Disease | Disease | Benign | damaging | tolerated |
| ENST00000305208.11 | rs1697475352 | Y79F | tolerated - low confidence | benign | low | neutral | neutral | Disease | Pathogenic | damaging | tolerated |
| ENST00000305208.11 | rs2125985436 | P80T | deleterious - low confidence | benign | low | neutral | neutral | Disease | Benign | damaging | tolerated |
| ENST00000305208.11 | rs2125985436 | P80A | tolerated - low confidence | benign | low | neutral | neutral | Disease | Benign | damaging | tolerated |
| ENST00000305208.11 | rs2125985436 | P80S | tolerated - low confidence | benign | low | neutral | neutral | Disease | Benign | damaging | tolerated |
| ENST00000305208.11 | rs2125985458 | P80H | deleterious - low confidence | benign | medium | Disease | neutral | Disease | Benign | damaging | tolerated |
| ENST00000305208.11 | rs2125985458 | P80R | deleterious - low confidence | benign | medium | Disease | neutral | Disease | Benign | damaging | tolerated |
| ENST00000305208.11 | rs2125985458 | P80L | deleterious - low confidence | benign | medium | Disease | neutral | Disease | Benign | damaging | tolerated |
| ENST00000305208.11 | rs2125985500 | V81M | deleterious - low confidence | probably damaging | medium | neutral | neutral | Disease | Benign | damaging | tolerated |
| ENST00000305208.11 | rs2125985500 | V81L | deleterious - low confidence | benign | medium | neutral | neutral | Disease | Benign | damaging | tolerated |
| ENST00000305208.11 | rs1169787218 | V81E | deleterious - low confidence | probably damaging | medium | Disease | neutral | Disease | Pathogenic | damaging | tolerated |
| ENST00000305208.11 | rs1169787218 | V81A | deleterious - low confidence | benign | medium | neutral | neutral | Disease | Benign | damaging | tolerated |
| ENST00000305208.11 | rs1397137648 | P82T | tolerated - low confidence | benign | medium | neutral | neutral | Disease | Benign | damaging | tolerated |
| ENST00000305208.11 | rs1397137648 | P82A | deleterious - low confidence | benign | medium | neutral | neutral | Disease | Benign | damaging | tolerated |
| ENST00000305208.11 | rs1397137648 | P82S | tolerated - low confidence | benign | low | neutral | neutral | Disease | Benign | damaging | tolerated |
| ENST00000305208.11 | rs2125985552 | P82R | deleterious - low confidence | possibly damaging | medium | Disease | Disease | Disease | Benign | damaging | tolerated |
| ENST00000305208.11 | rs2125985552 | P82L | deleterious - low confidence | benign | medium | neutral | neutral | Disease | Benign | damaging | tolerated |
| ENST00000305208.11 | rs56059937 | F83I | deleterious - low confidence | benign | medium | neutral | neutral | neutral | Benign | damaging | tolerated |
| ENST00000305208.11 | rs56059937 | F83L | deleterious - low confidence | benign | low | neutral | neutral | neutral | Benign | damaging | tolerated |
| ENST00000305208.11 | rs1425697704 | F83C | deleterious - low confidence | benign | medium | Disease | neutral | Disease | Pathogenic | Benign | tolerated |
| ENST00000305208.11 | rs2125985634 | F83L | deleterious - low confidence | benign | low | neutral | neutral | neutral | Benign | Benign | tolerated |
| ENST00000305208.11 | rs2125985634 | F83L | deleterious - low confidence | benign | low | neutral | neutral | neutral | Benign | Benign | tolerated |
| ENST00000305208.11 | rs2125985654 | Q84R | tolerated - low confidence | benign | neutral | neutral | neutral | neutral | Benign | Benign | tolerated |
| ENST00000305208.11 | rs2125985654 | Q84L | deleterious - low confidence | benign | low | neutral | neutral | Disease | Benign | Benign | tolerated |
| ENST00000305208.11 | rs777452318 | R85G | deleterious - low confidence | benign | medium | neutral | neutral | Disease | Benign | Benign | tolerated |
| ENST00000305208.11 | rs1321370763 | R85K | tolerated - low confidence | benign | neutral | neutral | neutral | neutral | Benign | Benign | tolerated |
| ENST00000305208.11 | rs1697483152 | R85S | tolerated - low confidence | benign | low | neutral | neutral | neutral | Benign | Benign | tolerated |
| ENST00000305208.11 | rs2125985711 | E86K | deleterious - low confidence | benign | low | Disease | neutral | Disease | Benign | damaging | tolerated |
| ENST00000305208.11 | rs1287938183 | E86A | deleterious - low confidence | benign | medium | neutral | neutral | Disease | Benign | damaging | tolerated |
| ENST00000305208.11 | rs1287938183 | E86G | deleterious - low confidence | possibly damaging | medium | neutral | neutral | Disease | Benign | damaging | tolerated |
| ENST00000305208.11 | rs2125985740 | D87N | tolerated - low confidence | benign | low | neutral | neutral | Disease | Benign | Benign | tolerated |
| ENST00000305208.11 | rs2125985740 | D87H | tolerated - low confidence | benign | low | neutral | neutral | Disease | Benign | Benign | tolerated |
| ENST00000305208.11 | rs533766461 | D87G | deleterious - low confidence | benign | low | neutral | neutral | Disease | Benign | Benign | tolerated |
| ENST00000305208.11 | rs533766461 | D87V | deleterious - low confidence | benign | low | Disease | neutral | Disease | Benign | Benign | tolerated |
| ENST00000305208.11 | rs2125985768 | D87E | tolerated - low confidence | benign | neutral | neutral | neutral | neutral | Benign | Benign | tolerated |
| ENST00000305208.11 | rs2125985791 | V88M | tolerated - low confidence | benign | neutral | neutral | neutral | neutral | Benign | Benign | tolerated |
| ENST00000305208.11 | rs2125985805 | V88E | deleterious - low confidence | benign | low | neutral | neutral | Disease | Pathogenic | Benign | tolerated |
| ENST00000305208.11 | rs2125985805 | V88A | deleterious - low confidence | benign | neutral | neutral | neutral | neutral | Benign | Benign | tolerated |
| ENST00000305208.11 | rs2125985830 | K89R | tolerated - low confidence | benign | neutral | neutral | neutral | neutral | Benign | Benign | tolerated |
| ENST00000305208.11 | rs2125985867 | E90K | tolerated - low confidence | benign | neutral | neutral | neutral | Disease | Benign | Benign | tolerated |
| ENST00000305208.11 | rs2125985867 | E90Q | tolerated - low confidence | benign | low | neutral | neutral | neutral | Benign | Benign | tolerated |
| ENST00000305208.11 | rs1285506084 | E90G | tolerated - low confidence | benign | low | neutral | neutral | neutral | Benign | Benign | tolerated |
| ENST00000305208.11 | rs1285506084 | E90V | tolerated - low confidence | benign | low | neutral | neutral | Disease | Benign | Benign | tolerated |
| ENST00000305208.11 | rs2125985913 | E90D | tolerated - low confidence | benign | neutral | neutral | neutral | neutral | Benign | Benign | tolerated |
| ENST00000305208.11 | rs2125985928 | S91T | tolerated - low confidence | benign | neutral | neutral | neutral | neutral | Benign | Benign | tolerated |
| ENST00000305208.11 | rs776620061 | S91Y | tolerated - low confidence | benign | low | neutral | neutral | neutral | Benign | Benign | tolerated |
| ENST00000305208.11 | rs776620061 | S91C | tolerated - low confidence | possibly damaging | medium | neutral | neutral | neutral | Benign | Benign | tolerated |
| ENST00000305208.11 | rs1218423176 | F92S | deleterious - low confidence | possibly damaging | medium | Disease | neutral | Disease | Pathogenic | damaging | tolerated |
| ENST00000305208.11 | rs2125985983 | V93D | tolerated - low confidence | benign | neutral | neutral | neutral | neutral | Benign | Benign | tolerated |
| ENST00000305208.11 | rs2125985983 | V93A | tolerated - low confidence | benign | neutral | neutral | neutral | neutral | Benign | Benign | tolerated |
| ENST00000305208.11 | rs1697488656 | S94G | tolerated - low confidence | benign | neutral | neutral | neutral | neutral | Benign | Benign | tolerated |
| ENST00000305208.11 | rs1252353903 | S94N | tolerated - low confidence | benign | neutral | neutral | neutral | neutral | Benign | Benign | tolerated |
| ENST00000305208.11 | rs1252353903 | S94T | tolerated - low confidence | benign | neutral | neutral | neutral | neutral | Benign | Benign | tolerated |
| ENST00000305208.11 | rs146052898 | S94R | tolerated - low confidence | benign | neutral | neutral | neutral | Disease | Benign | Benign | tolerated |
| ENST00000305208.11 | rs2125986077 | L95I | tolerated - low confidence | benign | low | neutral | neutral | neutral | Benign | Benign | tolerated |
| ENST00000305208.11 | rs2125986077 | L95V | tolerated - low confidence | benign | low | neutral | neutral | neutral | Benign | Benign | tolerated |
| ENST00000305208.11 | rs2125986095 | L95H | tolerated - low confidence | benign | low | neutral | neutral | Disease | Benign | Benign | tolerated |
| ENST00000305208.11 | rs2125986095 | L95P | deleterious - low confidence | benign | medium | Disease | neutral | Disease | Pathogenic | Benign | tolerated |
| ENST00000305208.11 | rs1208621089 | G96R | tolerated - low confidence | benign | low | neutral | neutral | neutral | Pathogenic | damaging | tolerated |
| ENST00000305208.11 | rs1287104845 | G96E | tolerated - low confidence | benign | medium | neutral | neutral | neutral | Pathogenic | damaging | tolerated |
| ENST00000305208.11 | rs1287104845 | G96A | tolerated - low confidence | benign | low | neutral | neutral | neutral | Benign | damaging | tolerated |
| ENST00000305208.11 | rs1287104845 | G96V | tolerated - low confidence | benign | neutral | neutral | neutral | neutral | Benign | damaging | tolerated |
| ENST00000305208.11 | rs2125986208 | H97N | tolerated - low confidence | benign | neutral | neutral | neutral | neutral | Benign | damaging | tolerated |
| ENST00000305208.11 | rs2125986208 | H97D | tolerated - low confidence | benign | neutral | neutral | neutral | neutral | Benign | damaging | tolerated |
| ENST00000305208.11 | rs2125986208 | H97Y | tolerated - low confidence | benign | low | neutral | neutral | neutral | Benign | damaging | tolerated |
| ENST00000305208.11 | rs1222761790 | H97R | tolerated - low confidence | benign | neutral | neutral | neutral | neutral | Benign | damaging | tolerated |
| ENST00000305208.11 | rs2125986257 | H97Q | tolerated - low confidence | benign | neutral | neutral | neutral | neutral | Benign | damaging | tolerated |
| ENST00000305208.11 | rs2125986282 | N98D | tolerated - low confidence | benign | neutral | neutral | neutral | neutral | Benign | Benign | tolerated |
| ENST00000305208.11 | rs1277566287 | N98I | tolerated - low confidence | benign | neutral | neutral | neutral | Disease | Benign | Benign | tolerated |
| ENST00000305208.11 | rs138183896 | N98K | tolerated - low confidence | benign | neutral | neutral | neutral | neutral | Benign | Benign | tolerated |
| ENST00000305208.11 | rs2125986328 | F100I | deleterious - low confidence | benign | medium | neutral | neutral | Disease | Benign | damaging | tolerated |
| ENST00000305208.11 | rs374655757 | F100L | deleterious - low confidence | benign | low | neutral | neutral | Disease | Benign | damaging | tolerated |
| ENST00000305208.11 | rs2125986357 | E101K | tolerated - low confidence | benign | low | neutral | neutral | Disease | Benign | damaging | tolerated |
| ENST00000305208.11 | rs2125986357 | E101Q | tolerated - low confidence | benign | medium | neutral | neutral | Disease | Benign | damaging | tolerated |
| ENST00000305208.11 | rs2125986376 | E101G | tolerated - low confidence | benign | low | neutral | neutral | Disease | Benign | damaging | tolerated |
| ENST00000305208.11 | rs2125986394 | N102S | tolerated - low confidence | benign | low | neutral | neutral | neutral | Benign | Benign | tolerated |
| ENST00000305208.11 | rs2125986406 | N102K | tolerated - low confidence | benign | neutral | neutral | neutral | neutral | Benign | Benign | tolerated |
| ENST00000305208.11 | rs2125986416 | D103N | tolerated - low confidence | benign | low | neutral | neutral | neutral | Benign | Benign | tolerated |
| ENST00000305208.11 | rs2125986416 | D103H | tolerated - low confidence | benign | medium | neutral | neutral | Disease | Benign | Benign | tolerated |
| ENST00000305208.11 | rs763853117 | S104T | tolerated - low confidence | benign | low | neutral | neutral | neutral | Benign | Benign | tolerated |
| ENST00000305208.11 | rs763853117 | S104A | tolerated - low confidence | benign | low | neutral | neutral | neutral | Benign | Benign | tolerated |
| ENST00000305208.11 | rs1373476296 | S104Y | tolerated - low confidence | benign | medium | Disease | neutral | Disease | Benign | Benign | tolerated |
| ENST00000305208.11 | rs1373476296 | S104C | deleterious - low confidence | possibly damaging | medium | Disease | neutral | Disease | Benign | Benign | tolerated |
| ENST00000305208.11 | rs2125986480 | F105I | deleterious - low confidence | benign | medium | neutral | neutral | neutral | Benign | damaging | tolerated |
| ENST00000305208.11 | rs2125986492 | F105L | tolerated - low confidence | benign | low | neutral | neutral | neutral | Benign | damaging | tolerated |
| ENST00000305208.11 | rs2125986517 | L106Q | deleterious - low confidence | benign | medium | Disease | neutral | Disease | Benign | damaging | tolerated |
| ENST00000305208.11 | rs2125986542 | Q107K | tolerated - low confidence | benign | neutral | neutral | neutral | Disease | Benign | Benign | tolerated |
| ENST00000305208.11 | rs2125986542 | Q107E | tolerated - low confidence | benign | neutral | neutral | neutral | neutral | Benign | Benign | tolerated |
| ENST00000305208.11 | rs587784538 | R108C | deleterious - low confidence | possibly damaging | medium | Disease | neutral | Disease | Pathogenic | Benign | tolerated |
| ENST00000305208.11 | rs1389365341 | R108L | tolerated - low confidence | benign | low | neutral | neutral | Disease | Benign | Benign | tolerated |
| ENST00000305208.11 | rs144217005 | V109A | tolerated - low confidence | benign | low | neutral | neutral | neutral | Benign | Benign | tolerated |
| ENST00000305208.11 | rs558023429 | K111Q | tolerated - low confidence | benign | low | neutral | neutral | neutral | Benign | Benign | tolerated |
| ENST00000305208.11 | rs766978023 | T112R | tolerated - low confidence | benign | medium | neutral | Disease | Disease | Benign | damaging | tolerated |
| ENST00000305208.11 | rs1305127348 | Y113D | deleterious - low confidence | benign | medium | Disease | neutral | Disease | Pathogenic | damaging | tolerated |
| ENST00000305208.11 | rs1330446381 | K114N | tolerated - low confidence | benign | low | neutral | neutral | Disease | Benign | Benign | tolerated |
| ENST00000305208.11 | rs759620086 | K115E | tolerated - low confidence | benign | low | neutral | neutral | neutral | Benign | Benign | tolerated |
| ENST00000305208.11 | rs765441127 | K115N | tolerated - low confidence | benign | neutral | neutral | neutral | neutral | Benign | Benign | tolerated |
| ENST00000305208.11 | rs1467566165 | I116V | tolerated - low confidence | benign | neutral | neutral | neutral | neutral | Benign | Benign | tolerated |
| ENST00000305208.11 | rs140867457 | I116K | tolerated - low confidence | benign | low | Disease | neutral | Disease | Pathogenic | Benign | tolerated |
| ENST00000305208.11 | rs1697508597 | I116M | tolerated - low confidence | benign | neutral | neutral | neutral | neutral | Benign | Benign | tolerated |
| ENST00000305208.11 | rs1559407230 | K117E | tolerated - low confidence | benign | medium | neutral | neutral | Disease | Benign | Benign | tolerated |
| ENST00000305208.11 | rs1399740159 | K117R | tolerated - low confidence | benign | low | neutral | neutral | neutral | Benign | Benign | tolerated |
| ENST00000305208.11 | rs752920136 | K117N | tolerated - low confidence | benign | low | neutral | neutral | neutral | Benign | Benign | tolerated |
| ENST00000305208.11 | rs200734586 | K118N | tolerated - low confidence | benign | neutral | neutral | neutral | neutral | Benign | Benign | tolerated |
| ENST00000305208.11 | rs534521374 | D119G | tolerated - low confidence | benign | low | neutral | neutral | neutral | Benign | Benign | tolerated |
| ENST00000305208.11 | rs751311281 | S120T | tolerated - low confidence | benign | low | neutral | neutral | neutral | Benign | Benign | tolerated |
| ENST00000305208.11 | rs751311281 | S120P | deleterious - low confidence | possibly damaging | medium | Disease | neutral | Disease | Pathogenic | Benign | tolerated |
| ENST00000305208.11 | rs1697515818 | A121P | tolerated - low confidence | possibly damaging | medium | Disease | neutral | Disease | Benign | Benign | tolerated |
| ENST00000305208.11 | rs1358018329 | M122V | tolerated - low confidence | benign | neutral | neutral | neutral | neutral | Benign | Benign | tolerated |
| ENST00000305208.11 | rs1222597079 | L123F | tolerated - low confidence | benign | neutral | neutral | neutral | neutral | Benign | Benign | tolerated |
| ENST00000305208.11 | rs1293428675 | L123P | deleterious - low confidence | possibly damaging | medium | Disease | neutral | Disease | Pathogenic | Benign | tolerated |
| ENST00000305208.11 | rs757137518 | L124F | tolerated - low confidence | benign | low | neutral | neutral | neutral | Benign | Benign | tolerated |
| ENST00000305208.11 | rs1215512591 | G126A | tolerated - low confidence | benign | neutral | neutral | neutral | neutral | Benign | Benign | tolerated |
| ENST00000305208.11 | rs994268425 | C127S | deleterious - low confidence | possibly damaging | medium | Disease | neutral | Disease | Pathogenic | damaging | tolerated |
| ENST00000305208.11 | rs1575774776 | H129Y | tolerated - low confidence | benign | low | neutral | neutral | Disease | Benign | Benign | tolerated |
| ENST00000305208.11 | rs1191873899 | H129R | tolerated - low confidence | benign | low | neutral | neutral | neutral | Benign | Benign | tolerated |
| ENST00000305208.11 | rs1191873899 | H129L | tolerated - low confidence | benign | low | neutral | neutral | Disease | Benign | Benign | tolerated |
| ENST00000305208.11 | rs1697524405 | H129Q | tolerated - low confidence | benign | low | neutral | neutral | Disease | Benign | Benign | tolerated |
| ENST00000305208.11 | rs555950591 | L130F | deleterious - low confidence | possibly damaging | medium | neutral | neutral | Disease | Pathogenic | damaging | tolerated |
| ENST00000305208.11 | rs1697526377 | L131R | deleterious - low confidence | probably damaging | high | Disease | Disease | Disease | Pathogenic | damaging | tolerated |
| ENST00000305208.11 | rs769242961 | H132N | tolerated - low confidence | benign | low | neutral | neutral | neutral | Benign | Benign | tolerated |
| ENST00000305208.11 | rs769242961 | H132Y | tolerated - low confidence | benign | neutral | neutral | neutral | Disease | Benign | Benign | tolerated |
| ENST00000305208.11 | rs575799001 | N133S | tolerated - low confidence | possibly damaging | medium | neutral | neutral | Disease | Benign | damaging | tolerated |
| ENST00000305208.11 | rs1553620770 | N133K | deleterious - low confidence | possibly damaging | high | Disease | neutral | Disease | Benign | damaging | tolerated |
| ENST00000305208.11 | rs1697531217 | L136F | deleterious - low confidence | benign | medium | neutral | neutral | Disease | Pathogenic | damaging | tolerated |
| ENST00000305208.11 | rs1165584842 | M137L | tolerated - low confidence | benign | low | neutral | neutral | neutral | Pathogenic | Benign | tolerated |
| ENST00000305208.11 | rs1165584842 | M137V | tolerated - low confidence | benign | medium | neutral | neutral | Disease | Benign | Benign | tolerated |
| ENST00000305208.11 | rs1697532735 | M137T | deleterious - low confidence | benign | high | neutral | neutral | Disease | Pathogenic | Benign | tolerated |
| ENST00000305208.11 | rs1396464237 | L140R | deleterious - low confidence | probably damaging | high | Disease | Disease | Disease | Pathogenic | damaging | tolerated |
| ENST00000305208.11 | rs774109568 | A141E | tolerated - low confidence | benign | neutral | neutral | neutral | Disease | Benign | Benign | tolerated |
| ENST00000305208.11 | rs1559407347 | S143N | tolerated - low confidence | benign | medium | neutral | neutral | Disease | Benign | Benign | tolerated |
| ENST00000305208.11 | rs1697539194 | S144I | deleterious - low confidence | benign | medium | Disease | neutral | Disease | Benign | Benign | tolerated |
| ENST00000305208.11 | rs1289728507 | M148V | tolerated - low confidence | benign | neutral | neutral | neutral | neutral | Benign | Benign | tolerated |
| ENST00000305208.11 | rs371418452 | T150R | deleterious - low confidence | probably damaging | high | Disease | Disease | Disease | Pathogenic | damaging | tolerated |
| ENST00000305208.11 | rs371418452 | T150M | tolerated - low confidence | possibly damaging | medium | neutral | neutral | neutral | Benign | damaging | tolerated |
| ENST00000305208.11 | rs1257207244 | D151G | deleterious - low confidence | probably damaging | high | Disease | Disease | Disease | Pathogenic | damaging | tolerated |
| ENST00000305208.11 | rs760143782 | P152H | deleterious - low confidence | probably damaging | high | Disease | Disease | Disease | Pathogenic | damaging | tolerated |
| ENST00000305208.11 | rs760143782 | P152R | deleterious - low confidence | probably damaging | high | Disease | Disease | Disease | Pathogenic | damaging | tolerated |
| ENST00000305208.11 | rs1697549301 | F153Y | tolerated - low confidence | possibly damaging | high | neutral | neutral | Disease | Benign | damaging | tolerated |
| ENST00000305208.11 | rs772315032 | F153L | tolerated - low confidence | benign | medium | neutral | neutral | Disease | Benign | damaging | tolerated |
| ENST00000305208.11 | rs762472856 | S157N | deleterious - low confidence | benign | low | neutral | neutral | Disease | Benign | Benign | tolerated |
| ENST00000305208.11 | rs1301858882 | P158T | tolerated - low confidence | benign | low | neutral | neutral | neutral | Benign | Benign | tolerated |
| ENST00000305208.11 | rs1301858882 | P158S | tolerated - low confidence | benign | neutral | neutral | neutral | neutral | Benign | Benign | tolerated |
| ENST00000305208.11 | rs1218181857 | P158H | tolerated - low confidence | possibly damaging | medium | neutral | Disease | neutral | Benign | Benign | tolerated |
| ENST00000305208.11 | rs1218181857 | P158R | tolerated - low confidence | benign | low | neutral | Disease | neutral | Pathogenic | Benign | tolerated |
| ENST00000305208.11 | rs587784539 | I159T | deleterious - low confidence | possibly damaging | medium | neutral | neutral | Disease | Pathogenic | damaging | tolerated |
| ENST00000305208.11 | rs199766420 | I159M | deleterious - low confidence | probably damaging | medium | neutral | neutral | Disease | Benign | damaging | tolerated |
| ENST00000305208.11 | rs764212365 | V160M | deleterious - low confidence | possibly damaging | medium | neutral | neutral | Disease | Benign | damaging | tolerated |
| ENST00000305208.11 | rs587784540 | V160E | deleterious - low confidence | probably damaging | medium | Disease | neutral | Disease | Pathogenic | damaging | tolerated |
| ENST00000305208.11 | rs1260954458 | A161T | deleterious - low confidence | probably damaging | high | Disease | neutral | Disease | Pathogenic | damaging | tolerated |
| ENST00000305208.11 | rs1260954458 | A161S | deleterious - low confidence | probably damaging | medium | Disease | neutral | Disease | Pathogenic | damaging | tolerated |
| ENST00000305208.11 | rs756981269 | A161V | deleterious - low confidence | probably damaging | high | Disease | neutral | Disease | Pathogenic | damaging | tolerated |
| ENST00000305208.11 | rs780985622 | Q162H | deleterious - low confidence | benign | neutral | neutral | neutral | neutral | Benign | Benign | tolerated |
| ENST00000305208.11 | rs541409164 | L166R | deleterious - low confidence | probably damaging | medium | Disease | neutral | Disease | Pathogenic | Benign | tolerated |
| ENST00000305208.11 | rs756010756 | T168A | tolerated - low confidence | benign | neutral | neutral | neutral | neutral | Benign | Benign | tolerated |
| ENST00000305208.11 | rs1185395607 | V169I | deleterious - low confidence | possibly damaging | medium | neutral | neutral | neutral | Benign | damaging | tolerated |
| ENST00000305208.11 | rs1185395607 | V169L | deleterious - low confidence | possibly damaging | medium | Disease | neutral | Disease | Pathogenic | damaging | tolerated |
| ENST00000305208.11 | rs1035248479 | F170L | deleterious - low confidence | benign | medium | neutral | neutral | Disease | Pathogenic | Benign | tolerated |
| ENST00000305208.11 | rs1365770885 | L172V | deleterious - low confidence | benign | low | neutral | neutral | neutral | Pathogenic | damaging | tolerated |
| ENST00000305208.11 | rs748734877 | L172F | tolerated - low confidence | benign | medium | neutral | neutral | Disease | Pathogenic | damaging | tolerated |
| ENST00000305208.11 | rs1464812223 | H173P | deleterious - low confidence | benign | neutral | Disease | neutral | Disease | Pathogenic | Benign | tolerated |
| ENST00000305208.11 | rs72551341 | L175Q | deleterious - low confidence | probably damaging | medium | neutral | Disease | Disease | Pathogenic | Benign | tolerated |
| ENST00000305208.11 | rs72551341 | L175R | deleterious - low confidence | possibly damaging | medium | Disease | Disease | Disease | Pathogenic | Benign | tolerated |
| ENST00000305208.11 | rs72551342 | C177R | deleterious - low confidence | probably damaging | medium | Disease | Disease | Disease | Pathogenic | damaging | tolerated |
| ENST00000305208.11 | rs1373930486 | C177Y | deleterious - low confidence | probably damaging | medium | neutral | Disease | Disease | Pathogenic | damaging | tolerated |
| ENST00000305208.11 | rs1210599713 | S178R | deleterious - low confidence | benign | medium | neutral | Disease | Disease | Pathogenic | damaging | tolerated |
| ENST00000305208.11 | rs1177412651 | S178N | tolerated - low confidence | benign | low | neutral | neutral | neutral | Benign | damaging | tolerated |
| ENST00000305208.11 | rs1390329998 | L179Q | deleterious - low confidence | probably damaging | medium | neutral | neutral | Disease | Benign | damaging | tolerated |
| ENST00000305208.11 | rs1657628450 | E180Q | deleterious - low confidence | benign | medium | neutral | neutral | Disease | Benign | Benign | tolerated |
| ENST00000305208.11 | rs1440575421 | T184S | tolerated - low confidence | benign | low | neutral | neutral | neutral | Benign | damaging | tolerated |
| ENST00000305208.11 | rs760019185 | P187A | deleterious - low confidence | probably damaging | high | neutral | neutral | Disease | Pathogenic | damaging | tolerated |
| ENST00000305208.11 | rs760019185 | P187S | deleterious - low confidence | probably damaging | medium | Disease | neutral | Disease | Pathogenic | damaging | tolerated |
| ENST00000305208.11 | rs1262253416 | N188S | tolerated - low confidence | benign | neutral | neutral | neutral | neutral | Benign | Benign | tolerated |
| ENST00000305208.11 | rs1262253416 | N188I | deleterious - low confidence | benign | medium | neutral | neutral | Disease | Benign | Benign | tolerated |
| ENST00000305208.11 | rs1697584249 | N188K | tolerated - low confidence | benign | medium | neutral | neutral | Disease | Pathogenic | Benign | tolerated |
| ENST00000305208.11 | rs770420506 | P189T | deleterious - low confidence | probably damaging | high | Disease | neutral | Disease | Pathogenic | damaging | tolerated |
| ENST00000305208.11 | rs1196570380 | S191P | deleterious - low confidence | probably damaging | high | Disease | neutral | Disease | Pathogenic | damaging | tolerated |
| ENST00000305208.11 | rs776126400 | Y192H | deleterious - low confidence | probably damaging | high | Disease | neutral | Disease | Pathogenic | damaging | tolerated |
| ENST00000305208.11 | rs201093245 | Y192C | deleterious - low confidence | probably damaging | high | Disease | Disease | Disease | Pathogenic | damaging | tolerated |
| ENST00000305208.11 | rs375974892 | V193M | deleterious - low confidence | possibly damaging | medium | neutral | neutral | Disease | Benign | Benign | tolerated |
| ENST00000305208.11 | rs2125988381 | V193E | deleterious - low confidence | probably damaging | high | Disease | Disease | Disease | Pathogenic | Benign | tolerated |
| ENST00000305208.11 | rs767983942 | P194A | deleterious - low confidence | probably damaging | high | neutral | neutral | Disease | Pathogenic | damaging | tolerated |
| ENST00000305208.11 | rs767983942 | P194S | deleterious - low confidence | probably damaging | high | Disease | neutral | Disease | Pathogenic | damaging | tolerated |
| ENST00000305208.11 | rs1697593103 | P194L | deleterious - low confidence | probably damaging | high | Disease | Disease | Disease | Pathogenic | damaging | tolerated |
| ENST00000305208.11 | rs761998591 | R195G | deleterious - low confidence | benign | medium | neutral | neutral | neutral | Pathogenic | damaging | tolerated |
| ENST00000305208.11 | rs767764203 | R195K | tolerated - low confidence | benign | low | neutral | neutral | neutral | Pathogenic | damaging | tolerated |
| ENST00000305208.11 | rs1553620840 | S198P | deleterious - low confidence | probably damaging | medium | Disease | neutral | Disease | Pathogenic | Benign | tolerated |
| ENST00000305208.11 | rs550460320 | S199C | tolerated - low confidence | benign | low | neutral | neutral | Disease | Pathogenic | Benign | tolerated |
| ENST00000305208.11 | rs550460320 | S199F | tolerated - low confidence | benign | neutral | neutral | neutral | Disease | Benign | Benign | tolerated |
| ENST00000305208.11 | rs2125988548 | H200Y | tolerated - low confidence | benign | neutral | neutral | neutral | neutral | Benign | Benign | tolerated |
| ENST00000305208.11 | rs1453639780 | H200P | tolerated - low confidence | benign | low | Disease | neutral | Disease | Pathogenic | Benign | tolerated |
| ENST00000305208.11 | rs1697601050 | D202H | deleterious - low confidence | possibly damaging | high | Disease | Disease | Disease | Pathogenic | damaging | tolerated |
| ENST00000305208.11 | rs1697601712 | H203Y | deleterious - low confidence | possibly damaging | medium | neutral | Disease | Disease | Benign | Benign | tolerated |
| ENST00000305208.11 | rs1171527500 | M204T | deleterious - low confidence | probably damaging | high | Disease | neutral | Disease | Pathogenic | damaging | tolerated |
| ENST00000305208.11 | rs1171527500 | M204R | deleterious - low confidence | probably damaging | high | Disease | Disease | Disease | Pathogenic | damaging | tolerated |
| ENST00000305208.11 | rs1697604278 | M204I | deleterious - low confidence | probably damaging | high | Disease | Disease | Disease | Pathogenic | damaging | tolerated |
| ENST00000305208.11 | rs766170365 | T205I | deleterious - low confidence | possibly damaging | high | Disease | neutral | Disease | Benign | damaging | tolerated |
| ENST00000305208.11 | rs1375729895 | Q208R | deleterious - low confidence | possibly damaging | high | Disease | neutral | Disease | Pathogenic | damaging | tolerated |
| ENST00000305208.11 | rs72551343 | R209G | deleterious - low confidence | probably damaging | high | Disease | Disease | Disease | Benign | damaging | tolerated |
| ENST00000305208.11 | rs72551343 | R209W | deleterious - low confidence | probably damaging | high | Disease | Disease | Disease | Pathogenic | damaging | tolerated |
| ENST00000305208.11 | rs747662045 | R209Q | deleterious - low confidence | probably damaging | high | Disease | Disease | Disease | Pathogenic | damaging | tolerated |
| ENST00000305208.11 | rs747662045 | R209P | deleterious - low confidence | probably damaging | high | neutral | Disease | Disease | Pathogenic | damaging | tolerated |
| ENST00000305208.11 | rs758093591 | V210M | deleterious - low confidence | possibly damaging | medium | Disease | neutral | Disease | Benign | damaging | tolerated |
| ENST00000305208.11 | rs758093591 | V210L | tolerated - low confidence | benign | medium | neutral | neutral | Disease | Benign | damaging | tolerated |
| ENST00000305208.11 | rs1345834782 | V210G | deleterious - low confidence | probably damaging | high | Disease | neutral | Disease | Benign | damaging | tolerated |
| ENST00000305208.11 | rs1225128510 | L214F | deleterious - low confidence | benign | medium | neutral | neutral | Disease | Benign | damaging | tolerated |
| ENST00000305208.11 | rs1276169722 | L214H | deleterious - low confidence | probably damaging | high | Disease | neutral | Disease | Pathogenic | damaging | tolerated |
| ENST00000305208.11 | rs1276169722 | L214P | deleterious - low confidence | probably damaging | high | Disease | Disease | Disease | Pathogenic | damaging | tolerated |
| ENST00000305208.11 | rs144398951 | I215V | tolerated - low confidence | benign | neutral | neutral | neutral | neutral | Benign | Benign | tolerated |
| ENST00000305208.11 | rs1360622092 | I215T | tolerated - low confidence | benign | low | neutral | neutral | Disease | Benign | Benign | tolerated |
| ENST00000305208.11 | rs1697618719 | A216T | tolerated - low confidence | benign | neutral | neutral | neutral | neutral | Benign | Benign | tolerated |
| ENST00000305208.11 | rs907848517 | A216D | tolerated - low confidence | benign | low | Disease | neutral | Disease | Pathogenic | Benign | tolerated |
| ENST00000305208.11 | rs907848517 | A216V | tolerated - low confidence | benign | low | neutral | neutral | neutral | Benign | Benign | tolerated |
| ENST00000305208.11 | rs1253140529 | Q219E | tolerated - low confidence | benign | neutral | neutral | neutral | neutral | Benign | Benign | tolerated |
| ENST00000305208.11 | rs1487587152 | Q219R | deleterious - low confidence | benign | low | neutral | neutral | Disease | Pathogenic | Benign | tolerated |
| ENST00000305208.11 | rs1697622087 | Q219H | tolerated - low confidence | benign | low | neutral | neutral | Disease | Pathogenic | Benign | tolerated |
| ENST00000305208.11 | rs746225571 | F221V | tolerated - low confidence | benign | low | neutral | neutral | neutral | Benign | Benign | tolerated |
| ENST00000305208.11 | rs2125989032 | C223Y | deleterious - low confidence | possibly damaging | medium | Disease | neutral | neutral | Pathogenic | damaging | tolerated |
| ENST00000305208.11 | rs775797489 | D224N | tolerated - low confidence | benign | neutral | neutral | neutral | Disease | Benign | Benign | tolerated |
| ENST00000305208.11 | rs775797489 | D224H | tolerated - low confidence | benign | neutral | neutral | neutral | Disease | Benign | Benign | tolerated |
| ENST00000305208.11 | rs976503144 | D224V | tolerated - low confidence | benign | low | neutral | neutral | Disease | Pathogenic | Benign | tolerated |
| ENST00000305208.11 | rs144721642 | V225M | tolerated - low confidence | benign | low | neutral | neutral | neutral | Benign | Benign | tolerated |
| ENST00000305208.11 | rs144721642 | V225L | tolerated - low confidence | benign | neutral | neutral | neutral | neutral | Benign | Benign | tolerated |
| ENST00000305208.11 | rs35003977 | V225G | tolerated - low confidence | benign | medium | neutral | neutral | Disease | Benign | Benign | tolerated |
| ENST00000305208.11 | rs761943511 | V226I | tolerated - low confidence | benign | low | neutral | neutral | neutral | Benign | Benign | tolerated |
| ENST00000305208.11 | rs1355767569 | V226A | tolerated - low confidence | benign | low | neutral | neutral | Disease | Benign | Benign | tolerated |
| ENST00000305208.11 | rs767709240 | S228F | deleterious - low confidence | possibly damaging | medium | neutral | neutral | Disease | Benign | damaging | tolerated |
| ENST00000305208.11 | rs35350960 | P229Q | deleterious - low confidence | benign | medium | Disease | neutral | Disease | Pathogenic | damaging | tolerated |
| ENST00000305208.11 | rs35350960 | P229L | deleterious - low confidence | benign | medium | Disease | Disease | Disease | Pathogenic | damaging | tolerated |
| ENST00000305208.11 | rs754922685 | Y230C | deleterious - low confidence | probably damaging | high | Disease | neutral | Disease | Pathogenic | damaging | tolerated |
| ENST00000305208.11 | rs147640261 | T232N | tolerated - low confidence | benign | neutral | neutral | neutral | neutral | Benign | Benign | tolerated |
| ENST00000305208.11 | rs147640261 | T232I | tolerated - low confidence | benign | neutral | neutral | neutral | neutral | Benign | Benign | tolerated |
| ENST00000305208.11 | rs2125989311 | L233I | tolerated - low confidence | benign | low | neutral | neutral | neutral | Benign | damaging | tolerated |
| ENST00000305208.11 | rs72551344 | L233R | deleterious - low confidence | probably damaging | high | Disease | neutral | Disease | Pathogenic | damaging | tolerated |
| ENST00000305208.11 | rs1247729976 | S235L | deleterious - low confidence | possibly damaging | medium | neutral | neutral | Disease | Pathogenic | damaging | tolerated |
| ENST00000305208.11 | rs367668492 | E236Q | deleterious - low confidence | benign | medium | neutral | neutral | Disease | Benign | damaging | tolerated |
| ENST00000305208.11 | rs1292729265 | R240G | deleterious - low confidence | possibly damaging | medium | neutral | Disease | Disease | Pathogenic | damaging | tolerated |
| ENST00000305208.11 | rs145912061 | R240K | tolerated - low confidence | benign | low | neutral | neutral | Disease | Benign | damaging | tolerated |
| ENST00000305208.11 | rs1395770157 | E241G | deleterious - low confidence | benign | low | neutral | neutral | neutral | Benign | Benign | tolerated |
| ENST00000305208.11 | rs756396505 | T243A | deleterious - low confidence | benign | medium | neutral | neutral | neutral | Benign | Benign | tolerated |
| ENST00000305208.11 | rs568151745 | T243N | deleterious - low confidence | benign | medium | neutral | neutral | neutral | Benign | Benign | tolerated |
| ENST00000305208.11 | rs568151745 | T243S | tolerated - low confidence | benign | neutral | neutral | neutral | neutral | Benign | Benign | tolerated |
| ENST00000305208.11 | rs1188733606 | V244I | tolerated - low confidence | benign | neutral | neutral | neutral | neutral | Benign | Benign | tolerated |
| ENST00000305208.11 | rs1188733606 | V244L | tolerated - low confidence | benign | neutral | neutral | neutral | neutral | Benign | Benign | tolerated |
| ENST00000305208.11 | rs2125989612 | D246N | deleterious - low confidence | possibly damaging | medium | neutral | neutral | neutral | Benign | damaging | tolerated |
| ENST00000305208.11 | rs2125989634 | D246E | tolerated - low confidence | benign | neutral | neutral | neutral | neutral | Benign | damaging | tolerated |
| ENST00000305208.11 | rs774774935 | S249N | deleterious - low confidence | benign | high | neutral | neutral | neutral | Benign | damaging | tolerated |
| ENST00000305208.11 | rs774774935 | S249I | deleterious - low confidence | probably damaging | high | neutral | neutral | Disease | Benign | damaging | tolerated |
| ENST00000305208.11 | rs57307513 | S250P | tolerated - low confidence | benign | low | Disease | neutral | neutral | Pathogenic | Benign | tolerated |
| ENST00000305208.11 | rs1697654904 | V253L | tolerated - low confidence | benign | low | neutral | neutral | neutral | Benign | Benign | tolerated |
| ENST00000305208.11 | rs1315871466 | V253D | deleterious - low confidence | possibly damaging | medium | Disease | Disease | Disease | Pathogenic | Benign | tolerated |
| ENST00000305208.11 | rs1559407924 | L255Q | deleterious - low confidence | probably damaging | high | Disease | Disease | Disease | Pathogenic | damaging | tolerated |
| ENST00000305208.11 | rs1559407933 | F256L | tolerated - low confidence | benign | neutral | neutral | neutral | neutral | Benign | Benign | tolerated |
| ENST00000305208.11 | rs1559407933 | F256L | tolerated - low confidence | benign | neutral | neutral | neutral | neutral | Benign | Benign | tolerated |
| ENST00000305208.11 | rs1697660244 | D259Y | deleterious - low confidence | probably damaging | medium | Disease | Disease | Disease | Pathogenic | damaging | tolerated |
| ENST00000305208.11 | rs772142239 | V261A | deleterious - low confidence | benign | low | neutral | neutral | neutral | Benign | damaging | tolerated |
| ENST00000305208.11 | rs1451879441 | K262M | tolerated - low confidence | benign | neutral | neutral | neutral | neutral | Benign | Benign | tolerated |
| ENST00000305208.11 | rs1315793944 | D263H | deleterious - low confidence | benign | medium | Disease | Disease | Disease | Pathogenic | Benign | tolerated |
| ENST00000305208.11 | rs760907397 | P265A | deleterious - low confidence | possibly damaging | medium | Disease | neutral | neutral | Pathogenic | damaging | tolerated |
| ENST00000305208.11 | rs760907397 | P265S | deleterious - low confidence | possibly damaging | medium | Disease | Disease | Disease | Pathogenic | damaging | tolerated |
| ENST00000305208.11 | rs141950052 | P267R | deleterious - low confidence | probably damaging | high | Disease | Disease | Disease | Pathogenic | damaging | tolerated |
| ENST00000305208.11 | rs1697668755 | I268V | tolerated - low confidence | benign | neutral | neutral | neutral | neutral | Benign | Benign | tolerated |
| ENST00000305208.11 | rs765208275 | P270S | deleterious - low confidence | probably damaging | high | Disease | Disease | Disease | Pathogenic | damaging | tolerated |
| ENST00000305208.11 | rs1487885628 | M272T | tolerated - low confidence | benign | medium | Disease | neutral | Disease | Benign | damaging | tolerated |
| ENST00000305208.11 | rs371726341 | M272I | tolerated - low confidence | benign | low | Disease | neutral | neutral | Benign | damaging | tolerated |
| ENST00000305208.11 | rs143072292 | V273F | deleterious - low confidence | benign | high | Disease | neutral | Disease | Pathogenic | damaging | tolerated |
| ENST00000305208.11 | rs1236186433 | V275F | deleterious - low confidence | possibly damaging | medium | Disease | Disease | Disease | Pathogenic | Benign | tolerated |
| ENST00000305208.11 | rs72551345 | G276R | deleterious - low confidence | probably damaging | high | Disease | Disease | Disease | Pathogenic | damaging | damaging |
| ENST00000305208.11 | rs72551345 | G276C | deleterious - low confidence | probably damaging | high | Disease | Disease | Disease | Pathogenic | damaging | damaging |
| ENST00000305208.11 | rs570314042 | G276D | deleterious - low confidence | probably damaging | high | Disease | Disease | Disease | Pathogenic | damaging | damaging |
| ENST00000305208.11 | rs570314042 | G276V | deleterious - low confidence | probably damaging | high | Disease | Disease | Disease | Pathogenic | damaging | damaging |
| ENST00000305208.11 | rs1445934796 | I278S | deleterious - low confidence | possibly damaging | medium | Disease | neutral | Disease | Pathogenic | damaging | tolerated |
| ENST00000305208.11 | rs397978903 | N279Y | deleterious - low confidence | probably damaging | medium | Disease | neutral | Disease | Pathogenic | damaging | tolerated |
| ENST00000305208.11 | rs1406825274 | N279K | deleterious - low confidence | possibly damaging | high | Disease | neutral | neutral | Pathogenic | damaging | tolerated |
| ENST00000305208.11 | rs754213125 | C280R | deleterious - low confidence | probably damaging | high | Disease | Disease | Disease | Pathogenic | damaging | tolerated |
| ENST00000305208.11 | rs754213125 | C280G | deleterious - low confidence | probably damaging | medium | Disease | Disease | Disease | Pathogenic | damaging | tolerated |
| ENST00000305208.11 | rs281865418 | C280W | deleterious - low confidence | probably damaging | high | Disease | Disease | Disease | Pathogenic | damaging | tolerated |
| ENST00000305208.11 | rs755308142 | Q283R | tolerated - low confidence | benign | neutral | neutral | neutral | neutral | Benign | Benign | tolerated |
| ENST00000305208.11 | rs1697686398 | N284S | deleterious - low confidence | benign | neutral | neutral | neutral | neutral | Benign | Benign | tolerated |
| ENST00000305208.11 | rs1337384419 | S287P | tolerated - low confidence | benign | low | neutral | neutral | neutral | Benign | Benign | tolerated |
| ENST00000305208.11 | rs1264463667 | S287F | deleterious - low confidence | probably damaging | high | Disease | Disease | Disease | Benign | damaging | tolerated |
| ENST00000305208.11 | rs1575779799 | Q288K | tolerated - low confidence | benign | low | neutral | neutral | neutral | Benign | damaging | tolerated |
| ENST00000305208.11 | rs1575779799 | Q288E | tolerated - low confidence | benign | low | neutral | neutral | neutral | Benign | damaging | tolerated |
| ENST00000305208.11 | rs1384998466 | Q288R | deleterious - low confidence | benign | medium | neutral | neutral | Disease | Benign | damaging | tolerated |
| ENST00000305208.11 | rs1699312336 | E289K | deleterious - low confidence | possibly damaging | medium | Disease | neutral | Disease | Benign | damaging | tolerated |
| ENST00000305208.11 | rs1316565909 | E289G | deleterious - low confidence | probably damaging | medium | Disease | Disease | Disease | Benign | damaging | tolerated |
| ENST00000305208.11 | rs1699312862 | E291K | deleterious - low confidence | possibly damaging | high | Disease | Disease | Disease | Pathogenic | damaging | tolerated |
| ENST00000305208.11 | rs758873309 | A292D | deleterious - low confidence | benign | low | neutral | neutral | Disease | Benign | damaging | tolerated |
| ENST00000305208.11 | rs758873309 | A292G | tolerated - low confidence | benign | medium | neutral | neutral | Disease | Benign | damaging | tolerated |
| ENST00000305208.11 | rs758873309 | A292V | deleterious - low confidence | possibly damaging | medium | neutral | neutral | Disease | Pathogenic | damaging | tolerated |
| ENST00000305208.11 | rs72551347 | I294T | deleterious - low confidence | benign | low | Disease | neutral | Disease | Pathogenic | Benign | tolerated |
| ENST00000305208.11 | rs928021157 | S297Y | deleterious - low confidence | probably damaging | high | Disease | Disease | Disease | Pathogenic | damaging | tolerated |
| ENST00000305208.11 | rs1699315314 | G298E | deleterious - low confidence | probably damaging | medium | Disease | neutral | Disease | Pathogenic | damaging | tolerated |
| ENST00000305208.11 | rs1055696021 | H300R | deleterious - low confidence | probably damaging | high | Disease | neutral | Disease | Benign | damaging | tolerated |
| ENST00000305208.11 | rs1451220464 | G301E | deleterious - low confidence | probably damaging | high | neutral | Disease | Disease | Pathogenic | damaging | tolerated |
| ENST00000305208.11 | rs1199920513 | I302V | tolerated - low confidence | benign | neutral | neutral | neutral | neutral | Benign | damaging | tolerated |
| ENST00000305208.11 | rs747099261 | I302T | deleterious - low confidence | possibly damaging | medium | neutral | neutral | Disease | Pathogenic | damaging | tolerated |
| ENST00000305208.11 | rs770930440 | V303M | deleterious - low confidence | probably damaging | high | Disease | Disease | Disease | Pathogenic | damaging | tolerated |
| ENST00000305208.11 | rs1699316631 | V304L | deleterious - low confidence | benign | medium | neutral | neutral | Disease | Benign | damaging | tolerated |
| ENST00000305208.11 | rs781412186 | V304A | deleterious - low confidence | probably damaging | medium | neutral | Disease | Disease | Pathogenic | damaging | tolerated |
| ENST00000305208.11 | rs1169717734 | S306Y | deleterious - low confidence | probably damaging | high | Disease | Disease | Disease | Pathogenic | damaging | tolerated |
| ENST00000305208.11 | rs1169717734 | S306F | deleterious - low confidence | probably damaging | high | Disease | Disease | Disease | Benign | damaging | tolerated |
| ENST00000305208.11 | rs746111352 | G308R | deleterious - low confidence | probably damaging | high | Disease | Disease | Disease | Pathogenic | damaging | damaging |
| ENST00000305208.11 | rs62625011 | G308E | deleterious - low confidence | probably damaging | high | Disease | Disease | Disease | Benign | damaging | damaging |
| ENST00000305208.11 | rs1301476253 | S309L | deleterious - low confidence | probably damaging | high | Disease | Disease | Disease | Pathogenic | damaging | tolerated |
| ENST00000305208.11 | rs1699317728 | M310V | deleterious - low confidence | possibly damaging | medium | neutral | neutral | Disease | Benign | damaging | tolerated |
| ENST00000305208.11 | rs1699317899 | M310R | deleterious - low confidence | probably damaging | high | Disease | Disease | Disease | Pathogenic | damaging | tolerated |
| ENST00000305208.11 | rs2126030402 | V311I | deleterious - low confidence | possibly damaging | low | neutral | neutral | Disease | Benign | damaging | tolerated |
| ENST00000305208.11 | rs1699318263 | S312L | deleterious - low confidence | possibly damaging | high | Disease | neutral | Disease | Benign | damaging | tolerated |
| ENST00000305208.11 | rs1575824807 | I314L | deleterious - low confidence | benign | low | neutral | neutral | Disease | Benign | damaging | tolerated |
| ENST00000305208.11 | rs1575824807 | I314V | deleterious - low confidence | possibly damaging | medium | neutral | neutral | Disease | Benign | damaging | tolerated |
| ENST00000305208.11 | rs769666128 | P315T | deleterious - low confidence | probably damaging | low | neutral | neutral | Disease | Benign | damaging | tolerated |
| ENST00000305208.11 | rs769666128 | P315S | deleterious - low confidence | probably damaging | medium | Disease | neutral | Disease | Benign | damaging | tolerated |
| ENST00000305208.11 | rs114000345 | K317E | deleterious - low confidence | benign | neutral | neutral | neutral | Disease | Benign | damaging | tolerated |
| ENST00000305208.11 | rs562389152 | A319P | deleterious - low confidence | probably damaging | high | Disease | neutral | Disease | Pathogenic | damaging | tolerated |
| ENST00000305208.11 | rs775405878 | A319V | deleterious - low confidence | probably damaging | medium | neutral | neutral | Disease | Benign | damaging | tolerated |
| ENST00000305208.11 | rs749159613 | M320V | deleterious - low confidence | benign | medium | neutral | neutral | Disease | Benign | damaging | tolerated |
| ENST00000305208.11 | rs2126030462 | M320T | tolerated - low confidence | benign | medium | neutral | neutral | Disease | Benign | damaging | tolerated |
| ENST00000305208.11 | rs200903749 | I322V | deleterious - low confidence | possibly damaging | medium | neutral | neutral | Disease | Benign | damaging | tolerated |
| ENST00000305208.11 | rs17851756 | I322T | deleterious - low confidence | probably damaging | high | Disease | neutral | Disease | Pathogenic | damaging | tolerated |
| ENST00000305208.11 | rs774438667 | A323S | deleterious - low confidence | probably damaging | medium | Disease | neutral | Disease | Benign | damaging | tolerated |
| ENST00000305208.11 | rs761316504 | A323V | deleterious - low confidence | possibly damaging | medium | Disease | neutral | Disease | Benign | damaging | tolerated |
| ENST00000305208.11 | rs1269029361 | A325T | deleterious - low confidence | probably damaging | medium | Disease | neutral | Disease | Pathogenic | damaging | tolerated |
| ENST00000305208.11 | rs372326047 | L326S | deleterious - low confidence | probably damaging | high | Disease | neutral | Disease | Pathogenic | damaging | tolerated |
| ENST00000305208.11 | rs1339405023 | I329L | deleterious - low confidence | probably damaging | low | neutral | neutral | neutral | Benign | damaging | tolerated |
| ENST00000305208.11 | rs202035422 | I329T | deleterious - low confidence | probably damaging | high | Disease | neutral | Disease | Benign | damaging | tolerated |
| ENST00000305208.11 | rs72551348 | Q331R | deleterious - low confidence | probably damaging | high | Disease | Disease | Disease | Pathogenic | damaging | tolerated |
| ENST00000305208.11 | rs1699322110 | T332A | deleterious - low confidence | possibly damaging | medium | neutral | neutral | Disease | Benign | damaging | tolerated |
| ENST00000305208.11 | rs1699322267 | T332R | deleterious - low confidence | benign | low | neutral | neutral | Disease | Benign | damaging | tolerated |
| ENST00000305208.11 | rs757687307 | L334Q | deleterious - low confidence | probably damaging | high | Disease | Disease | Disease | Pathogenic | damaging | tolerated |
| ENST00000305208.11 | rs1181740769 | W335S | deleterious - low confidence | probably damaging | high | Disease | Disease | Disease | Pathogenic | damaging | tolerated |
| ENST00000305208.11 | rs139607673 | R336W | deleterious - low confidence | probably damaging | high | Disease | Disease | Disease | Pathogenic | damaging | tolerated |
| ENST00000305208.11 | rs750453538 | R336Q | deleterious - low confidence | probably damaging | high | Disease | Disease | Disease | Pathogenic | damaging | tolerated |
| ENST00000305208.11 | rs750453538 | R336P | deleterious - low confidence | probably damaging | high | Disease | Disease | Disease | Pathogenic | damaging | tolerated |
| ENST00000305208.11 | rs1445321655 | Y337C | deleterious - low confidence | probably damaging | medium | Disease | neutral | Disease | Pathogenic | damaging | tolerated |
| ENST00000305208.11 | rs748591879 | T338A | deleterious - low confidence | probably damaging | medium | neutral | neutral | Disease | Benign | damaging | tolerated |
| ENST00000305208.11 | rs72551349 | R341G | tolerated - low confidence | benign | medium | neutral | neutral | Disease | Pathogenic | Benign | tolerated |
| ENST00000305208.11 | rs780354743 | R341Q | tolerated - low confidence | benign | medium | neutral | neutral | Disease | Benign | Benign | tolerated |
| ENST00000305208.11 | rs773195449 | P342T | deleterious - low confidence | possibly damaging | medium | Disease | neutral | Disease | Pathogenic | damaging | tolerated |
| ENST00000305208.11 | rs773195449 | P342S | deleterious - low confidence | probably damaging | high | Disease | neutral | Disease | Pathogenic | damaging | tolerated |
| ENST00000305208.11 | rs144978321 | S343W | deleterious - low confidence | probably damaging | medium | Disease | neutral | Disease | Benign | Benign | tolerated |
| ENST00000305208.11 | rs144978321 | S343L | deleterious - low confidence | possibly damaging | medium | neutral | neutral | neutral | Benign | Benign | tolerated |
| ENST00000305208.11 | rs1336725166 | N344S | deleterious - low confidence | probably damaging | medium | neutral | neutral | Disease | Benign | damaging | tolerated |
| ENST00000305208.11 | rs149750520 | N344K | deleterious - low confidence | probably damaging | medium | neutral | neutral | Disease | Benign | damaging | tolerated |
| ENST00000305208.11 | rs1699512749 | L345P | deleterious - low confidence | probably damaging | high | Disease | neutral | Disease | Pathogenic | damaging | tolerated |
| ENST00000305208.11 | rs771899094 | A346T | deleterious - low confidence | possibly damaging | medium | neutral | neutral | Disease | Benign | damaging | tolerated |
| ENST00000305208.11 | rs771899094 | A346S | tolerated - low confidence | benign | low | neutral | neutral | Disease | Benign | damaging | tolerated |
| ENST00000305208.11 | rs201372184 | A346V | deleterious - low confidence | possibly damaging | medium | neutral | neutral | Disease | Benign | damaging | tolerated |
| ENST00000305208.11 | rs1575830712 | N347K | tolerated - low confidence | benign | neutral | neutral | neutral | Disease | Benign | Benign | tolerated |
| ENST00000305208.11 | rs776227074 | T349K | deleterious - low confidence | probably damaging | high | Disease | Disease | Disease | Pathogenic | damaging | tolerated |
| ENST00000305208.11 | rs776227074 | T349M | deleterious - low confidence | probably damaging | high | neutral | neutral | Disease | Pathogenic | damaging | tolerated |
| ENST00000305208.11 | rs1699514998 | I350M | tolerated - low confidence | probably damaging | low | neutral | neutral | neutral | Benign | Benign | tolerated |
| ENST00000305208.11 | rs549391527 | L351V | deleterious - low confidence | possibly damaging | medium | neutral | neutral | Disease | Benign | damaging | tolerated |
| ENST00000305208.11 | rs549391527 | L351F | deleterious - low confidence | possibly damaging | medium | Disease | neutral | Disease | Benign | damaging | tolerated |
| ENST00000305208.11 | rs1699515433 | V352A | deleterious - low confidence | probably damaging | medium | neutral | neutral | Disease | Benign | damaging | tolerated |
| ENST00000305208.11 | rs1559414817 | W354R | deleterious - low confidence | probably damaging | high | Disease | Disease | Disease | Pathogenic | damaging | tolerated |
| ENST00000305208.11 | rs1699515943 | L355Q | deleterious - low confidence | probably damaging | high | Disease | Disease | Disease | Pathogenic | damaging | tolerated |
| ENST00000305208.11 | rs767850186 | P356T | deleterious - low confidence | probably damaging | high | Disease | Disease | Disease | Pathogenic | damaging | damaging |
| ENST00000305208.11 | rs72551351 | Q357P | deleterious - low confidence | probably damaging | high | Disease | Disease | Disease | Pathogenic | damaging | tolerated |
| ENST00000305208.11 | rs72551351 | Q357R | deleterious - low confidence | possibly damaging | high | Disease | Disease | Disease | Pathogenic | damaging | tolerated |
| ENST00000305208.11 | rs886044684 | N358T | deleterious - low confidence | probably damaging | medium | Disease | Disease | Disease | Pathogenic | damaging | tolerated |
| ENST00000305208.11 | rs886044684 | N358S | deleterious - low confidence | probably damaging | medium | neutral | neutral | Disease | Pathogenic | damaging | tolerated |
| ENST00000305208.11 | rs1699517542 | N358K | deleterious - low confidence | probably damaging | medium | Disease | Disease | Disease | Pathogenic | damaging | tolerated |
| ENST00000305208.11 | rs267599273 | D359N | deleterious - low confidence | probably damaging | high | Disease | Disease | Disease | Pathogenic | damaging | tolerated |
| ENST00000305208.11 | rs755218546 | G362S | deleterious - low confidence | probably damaging | high | Disease | Disease | Disease | Pathogenic | Benign | tolerated |
| ENST00000305208.11 | rs755218546 | G362C | deleterious - low confidence | probably damaging | medium | Disease | neutral | Disease | Pathogenic | Benign | tolerated |
| ENST00000305208.11 | rs752968297 | G362D | deleterious - low confidence | probably damaging | high | Disease | Disease | Disease | Pathogenic | Benign | tolerated |
| ENST00000305208.11 | rs752968297 | G362V | deleterious - low confidence | probably damaging | high | Disease | Disease | Disease | Pathogenic | Benign | tolerated |
| ENST00000305208.11 | rs34946978 | P364R | deleterious - low confidence | probably damaging | high | Disease | Disease | Disease | Benign | damaging | tolerated |
| ENST00000305208.11 | rs34946978 | P364L | deleterious - low confidence | probably damaging | high | neutral | Disease | Disease | Pathogenic | damaging | tolerated |
| ENST00000305208.11 | rs367784507 | M365V | deleterious - low confidence | benign | neutral | neutral | neutral | Disease | Benign | Benign | tolerated |
| ENST00000305208.11 | rs371224646 | M365T | deleterious - low confidence | benign | neutral | neutral | neutral | Disease | Benign | Benign | tolerated |
| ENST00000305208.11 | rs1699591522 | T366N | deleterious - low confidence | probably damaging | high | Disease | Disease | Disease | Pathogenic | damaging | tolerated |
| ENST00000305208.11 | rs55750087 | R367G | deleterious - low confidence | probably damaging | high | Disease | Disease | Disease | Pathogenic | damaging | tolerated |
| ENST00000305208.11 | rs55750087 | R367C | deleterious - low confidence | probably damaging | high | Disease | Disease | Disease | Pathogenic | damaging | tolerated |
| ENST00000305208.11 | rs374047963 | R367H | deleterious - low confidence | probably damaging | high | Disease | Disease | Disease | Pathogenic | damaging | tolerated |
| ENST00000305208.11 | rs374047963 | R367P | deleterious - low confidence | probably damaging | high | Disease | Disease | Disease | Pathogenic | damaging | tolerated |
| ENST00000305208.11 | rs374047963 | R367L | deleterious - low confidence | probably damaging | medium | Disease | Disease | Disease | Pathogenic | damaging | tolerated |
| ENST00000305208.11 | rs72551352 | A368T | deleterious - low confidence | probably damaging | high | Disease | Disease | Disease | Pathogenic | damaging | tolerated |
| ENST00000305208.11 | rs1218307967 | A368G | deleterious - low confidence | probably damaging | high | Disease | Disease | Disease | Pathogenic | damaging | tolerated |
| ENST00000305208.11 | rs748989741 | I370V | deleterious - low confidence | possibly damaging | low | neutral | Disease | Disease | Pathogenic | damaging | tolerated |
| ENST00000305208.11 | rs1285354199 | T371I | deleterious - low confidence | probably damaging | high | Disease | Disease | Disease | Pathogenic | damaging | tolerated |
| ENST00000305208.11 | rs1699594140 | H372N | deleterious - low confidence | probably damaging | high | Disease | Disease | Disease | Pathogenic | damaging | tolerated |
| ENST00000305208.11 | rs1699594140 | H372Y | deleterious - low confidence | probably damaging | high | Disease | Disease | Disease | Pathogenic | damaging | tolerated |
| ENST00000305208.11 | rs1276913504 | G374S | deleterious - low confidence | probably damaging | high | Disease | Disease | Disease | Pathogenic | damaging | damaging |
| ENST00000305208.11 | rs72551353 | S375F | deleterious - low confidence | probably damaging | medium | Disease | Disease | Disease | Pathogenic | damaging | tolerated |
| ENST00000305208.11 | rs1349037761 | H376R | deleterious - low confidence | possibly damaging | medium | Disease | Disease | Disease | Pathogenic | damaging | tolerated |
| ENST00000305208.11 | rs773679964 | G377S | deleterious - low confidence | probably damaging | medium | neutral | Disease | Disease | Pathogenic | damaging | tolerated |
| ENST00000305208.11 | rs1283652721 | G377V | deleterious - low confidence | probably damaging | high | Disease | Disease | Disease | Pathogenic | damaging | tolerated |
| ENST00000305208.11 | rs1699596168 | E380G | deleterious - low confidence | probably damaging | high | Disease | Disease | Disease | Pathogenic | damaging | tolerated |
| ENST00000305208.11 | rs771550944 | S381C | deleterious - low confidence | possibly damaging | low | Disease | Disease | Disease | Pathogenic | Benign | tolerated |
| ENST00000305208.11 | rs72551354 | S381R | deleterious - low confidence | possibly damaging | low | Disease | Disease | Disease | Pathogenic | Benign | tolerated |
| ENST00000305208.11 | rs777289979 | N384D | deleterious - low confidence | probably damaging | medium | Disease | Disease | Disease | Pathogenic | damaging | tolerated |
| ENST00000305208.11 | rs759467827 | N384S | deleterious - low confidence | probably damaging | medium | neutral | Disease | Disease | Benign | damaging | tolerated |
| ENST00000305208.11 | rs1699597581 | G385S | deleterious - low confidence | probably damaging | high | Disease | Disease | Disease | Pathogenic | damaging | damaging |
| ENST00000305208.11 | rs143573365 | V386I | deleterious - low confidence | possibly damaging | low | neutral | neutral | Disease | Benign | damaging | tolerated |
| ENST00000305208.11 | rs901936528 | P387S | deleterious - low confidence | probably damaging | high | Disease | Disease | Disease | Pathogenic | damaging | damaging |
| ENST00000305208.11 | rs1559415403 | P387R | deleterious - low confidence | probably damaging | high | Disease | Disease | Disease | Pathogenic | damaging | damaging |
| ENST00000305208.11 | rs1699598751 | M388V | deleterious - low confidence | probably damaging | low | Disease | neutral | Disease | Benign | damaging | tolerated |
| ENST00000305208.11 | rs527483899 | M388T | deleterious - low confidence | probably damaging | high | Disease | Disease | Disease | Pathogenic | damaging | tolerated |
| ENST00000305208.11 | rs1162609742 | M388I | deleterious - low confidence | probably damaging | medium | Disease | neutral | Disease | Pathogenic | damaging | tolerated |
| ENST00000305208.11 | rs1365887380 | V389L | deleterious - low confidence | benign | low | neutral | neutral | Disease | Pathogenic | damaging | tolerated |
| ENST00000305208.11 | rs1559415443 | M390I | deleterious - low confidence | possibly damaging | medium | Disease | neutral | Disease | Benign | Benign | tolerated |
| ENST00000305208.11 | rs1183097052 | M391V | deleterious - low confidence | benign | low | Disease | neutral | Disease | Benign | damaging | tolerated |
| ENST00000305208.11 | rs751355128 | M391I | deleterious - low confidence | benign | low | neutral | neutral | Disease | Benign | damaging | tolerated |
| ENST00000305208.11 | rs1286993592 | P392S | deleterious - low confidence | probably damaging | high | Disease | Disease | Disease | Pathogenic | damaging | damaging |
| ENST00000305208.11 | rs886043066 | P392L | deleterious - low confidence | probably damaging | high | Disease | Disease | Disease | Benign | damaging | damaging |
| ENST00000305208.11 | rs1430980091 | L393F | deleterious - low confidence | probably damaging | medium | Disease | neutral | Disease | Benign | damaging | tolerated |
| ENST00000305208.11 | rs1699600557 | F394L | deleterious - low confidence | possibly damaging | medium | Disease | neutral | Disease | Benign | damaging | tolerated |
| ENST00000305208.11 | rs367897068 | G395D | deleterious - low confidence | probably damaging | high | Disease | Disease | Disease | Pathogenic | damaging | tolerated |
| ENST00000305208.11 | rs367897068 | G395A | deleterious - low confidence | probably damaging | low | neutral | Disease | Disease | Pathogenic | damaging | tolerated |
| ENST00000305208.11 | rs367897068 | G395V | deleterious - low confidence | probably damaging | medium | Disease | Disease | Disease | Pathogenic | damaging | tolerated |
| ENST00000305208.11 | rs755527328 | M398T | deleterious - low confidence | probably damaging | medium | Disease | neutral | Disease | Benign | damaging | tolerated |
| ENST00000305208.11 | rs779591634 | D399G | deleterious - low confidence | probably damaging | medium | Disease | neutral | Disease | Pathogenic | damaging | tolerated |
| ENST00000305208.11 | rs28934877 | N400H | deleterious - low confidence | probably damaging | high | Disease | Disease | Disease | Pathogenic | damaging | tolerated |
| ENST00000305208.11 | rs28934877 | N400D | deleterious - low confidence | probably damaging | high | Disease | Disease | Disease | Pathogenic | damaging | tolerated |
| ENST00000305208.11 | rs754652167 | N400K | deleterious - low confidence | probably damaging | medium | Disease | Disease | Disease | Pathogenic | damaging | tolerated |
| ENST00000305208.11 | rs72551355 | A401P | deleterious - low confidence | probably damaging | high | Disease | Disease | Disease | Pathogenic | damaging | tolerated |
| ENST00000305208.11 | rs778766461 | R403S | deleterious - low confidence | probably damaging | medium | Disease | Disease | Disease | Pathogenic | damaging | tolerated |
| ENST00000305208.11 | rs778766461 | R403C | deleterious - low confidence | probably damaging | high | Disease | Disease | Disease | Pathogenic | damaging | tolerated |
| ENST00000305208.11 | rs140613392 | R403H | deleterious - low confidence | possibly damaging | low | neutral | Disease | Disease | Pathogenic | damaging | tolerated |
| ENST00000305208.11 | rs140613392 | R403L | deleterious - low confidence | probably damaging | medium | Disease | Disease | Disease | Pathogenic | damaging | tolerated |
| ENST00000305208.11 | rs777238544 | M404L | deleterious - low confidence | benign | low | neutral | neutral | Disease | Benign | Benign | tolerated |
| ENST00000305208.11 | rs777238544 | M404V | tolerated - low confidence | possibly damaging | low | neutral | neutral | Disease | Benign | Benign | tolerated |
| ENST00000305208.11 | rs549328655 | M404T | deleterious - low confidence | probably damaging | high | Disease | Disease | Disease | Pathogenic | Benign | tolerated |
| ENST00000305208.11 | rs2126038022 | M404I | tolerated - low confidence | benign | low | neutral | neutral | Disease | Benign | Benign | tolerated |
| ENST00000305208.11 | rs1699604029 | E405V | deleterious - low confidence | possibly damaging | low | neutral | neutral | Disease | Benign | damaging | tolerated |
| ENST00000305208.11 | rs1559415650 | K407R | tolerated - low confidence | benign | neutral | neutral | neutral | neutral | Benign | Benign | tolerated |
| ENST00000305208.11 | rs770254031 | G408E | deleterious - low confidence | probably damaging | high | Disease | Disease | Disease | Benign | damaging | tolerated |
| ENST00000305208.11 | rs1272833298 | A409V | deleterious - low confidence | possibly damaging | medium | neutral | neutral | Disease | Benign | damaging | tolerated |
| ENST00000305208.11 | rs1444362528 | G410E | deleterious - low confidence | probably damaging | medium | Disease | Disease | Disease | Pathogenic | damaging | tolerated |
| ENST00000305208.11 | rs36076514 | V411M | deleterious - low confidence | possibly damaging | high | Disease | neutral | Disease | Benign | damaging | tolerated |
| ENST00000305208.11 | rs36076514 | V411L | tolerated - low confidence | benign | low | neutral | neutral | Disease | Benign | damaging | tolerated |
| ENST00000305208.11 | rs1156645272 | T412S | tolerated - low confidence | benign | neutral | neutral | neutral | neutral | Benign | Benign | tolerated |
| ENST00000305208.11 | rs1699606066 | T412I | tolerated - low confidence | benign | low | neutral | neutral | Disease | Benign | Benign | tolerated |
| ENST00000305208.11 | rs763012065 | N414K | deleterious - low confidence | probably damaging | medium | neutral | neutral | neutral | Benign | damaging | tolerated |
| ENST00000305208.11 | rs774573761 | T419A | deleterious - low confidence | probably damaging | medium | neutral | Disease | neutral | Benign | damaging | tolerated |
| ENST00000305208.11 | rs866185120 | S420F | deleterious - low confidence | probably damaging | medium | Disease | neutral | Disease | Benign | damaging | tolerated |
| ENST00000305208.11 | rs750232648 | A426S | deleterious - low confidence | probably damaging | medium | Disease | neutral | neutral | Benign | damaging | tolerated |
| ENST00000305208.11 | rs756044146 | A426V | deleterious - low confidence | probably damaging | medium | Disease | Disease | Disease | Pathogenic | damaging | tolerated |
| ENST00000305208.11 | rs72551356 | K428E | deleterious - low confidence | probably damaging | medium | neutral | neutral | neutral | Pathogenic | damaging | tolerated |
| ENST00000305208.11 | rs1699608953 | K428R | deleterious - low confidence | benign | medium | neutral | neutral | neutral | Benign | damaging | tolerated |
| ENST00000305208.11 | rs1699609546 | I431V | deleterious - low confidence | possibly damaging | medium | neutral | neutral | neutral | Benign | damaging | tolerated |
| ENST00000305208.11 | rs1413528689 | N432D | deleterious - low confidence | benign | medium | Disease | neutral | neutral | Benign | damaging | tolerated |
| ENST00000305208.11 | rs754549295 | D433E | tolerated - low confidence | benign | low | Disease | neutral | neutral | Benign | Benign | tolerated |
| ENST00000305208.11 | rs1699610425 | K434E | deleterious - low confidence | probably damaging | medium | Disease | neutral | Disease | Benign | damaging | tolerated |
| ENST00000305208.11 | rs1699610736 | K434N | deleterious - low confidence | probably damaging | medium | Disease | neutral | neutral | Benign | damaging | tolerated |
| ENST00000305208.11 | rs1306719122 | S435N | deleterious - low confidence | possibly damaging | medium | Disease | neutral | Disease | Benign | damaging | tolerated |
| ENST00000305208.11 | rs1306719122 | S435T | deleterious - low confidence | benign | medium | neutral | neutral | neutral | Benign | damaging | tolerated |
| ENST00000305208.11 | rs1306719122 | S435I | deleterious - low confidence | probably damaging | medium | Disease | neutral | Disease | Benign | damaging | tolerated |
| ENST00000305208.11 | rs766626435 | Y436C | deleterious - low confidence | probably damaging | high | Disease | Disease | Disease | Pathogenic | damaging | tolerated |
| ENST00000305208.11 | rs766626435 | Y436F | deleterious - low confidence | probably damaging | medium | Disease | neutral | neutral | Pathogenic | damaging | tolerated |
| ENST00000305208.11 | rs1700497336 | K437N | deleterious - low confidence | probably damaging | high | Disease | neutral | Disease | Pathogenic | damaging | tolerated |
| ENST00000305208.11 | rs868814380 | E438K | deleterious - low confidence | probably damaging | medium | Disease | neutral | neutral | Benign | damaging | tolerated |
| ENST00000305208.11 | rs868814380 | E438Q | deleterious - low confidence | benign | medium | neutral | neutral | neutral | Benign | damaging | tolerated |
| ENST00000305208.11 | rs1365374902 | E438G | deleterious - low confidence | probably damaging | medium | Disease | neutral | neutral | Benign | damaging | tolerated |
| ENST00000305208.11 | rs1700498214 | I440V | deleterious - low confidence | benign | low | neutral | neutral | neutral | Benign | damaging | tolerated |
| ENST00000305208.11 | rs1575869883 | I440N | deleterious - low confidence | probably damaging | medium | Disease | neutral | neutral | Pathogenic | damaging | tolerated |
| ENST00000305208.11 | rs1289153891 | M441V | deleterious - low confidence | possibly damaging | medium | neutral | neutral | neutral | Benign | damaging | tolerated |
| ENST00000305208.11 | rs202172337 | M441K | tolerated - low confidence | benign | low | neutral | neutral | neutral | Benign | damaging | tolerated |
| ENST00000305208.11 | rs202172337 | M441T | deleterious - low confidence | benign | medium | neutral | neutral | neutral | Benign | damaging | tolerated |
| ENST00000305208.11 | rs202172337 | M441R | deleterious - low confidence | benign | medium | neutral | neutral | neutral | Benign | damaging | tolerated |
| ENST00000305208.11 | rs143033456 | R442C | deleterious - low confidence | probably damaging | high | Disease | Disease | Disease | Pathogenic | damaging | tolerated |
| ENST00000305208.11 | rs748166510 | R442H | tolerated - low confidence | benign | medium | neutral | neutral | neutral | Benign | damaging | tolerated |
| ENST00000305208.11 | rs748166510 | R442L | deleterious - low confidence | possibly damaging | medium | Disease | neutral | neutral | Benign | damaging | tolerated |
| ENST00000305208.11 | rs1445186018 | L443F | deleterious - low confidence | probably damaging | medium | neutral | neutral | neutral | Pathogenic | damaging | tolerated |
| ENST00000305208.11 | rs758411577 | L443P | deleterious - low confidence | probably damaging | high | Disease | Disease | Disease | Pathogenic | damaging | tolerated |
| ENST00000305208.11 | rs1575870013 | S444P | deleterious - low confidence | probably damaging | high | Disease | Disease | Disease | Pathogenic | damaging | tolerated |
| ENST00000305208.11 | rs1248654212 | S445N | deleterious - low confidence | possibly damaging | medium | neutral | neutral | neutral | Benign | Benign | tolerated |
| ENST00000305208.11 | rs778015980 | S445R | tolerated - low confidence | benign | neutral | neutral | neutral | neutral | Benign | Benign | tolerated |
| ENST00000305208.11 | rs568508589 | L446F | deleterious - low confidence | probably damaging | high | neutral | neutral | neutral | Benign | damaging | tolerated |
| ENST00000305208.11 | rs1700500987 | K448R | tolerated - low confidence | benign | low | neutral | neutral | neutral | Benign | damaging | tolerated |
| ENST00000305208.11 | rs1458644938 | D449N | deleterious - low confidence | probably damaging | high | Disease | Disease | neutral | Pathogenic | damaging | tolerated |
| ENST00000305208.11 | rs1482422312 | D449G | deleterious - low confidence | probably damaging | high | Disease | Disease | Disease | Pathogenic | damaging | tolerated |
| ENST00000305208.11 | rs1165346261 | D449E | deleterious - low confidence | probably damaging | high | Disease | neutral | neutral | Pathogenic | damaging | tolerated |
| ENST00000305208.11 | rs201427749 | R450C | deleterious - low confidence | probably damaging | high | Disease | Disease | Disease | Pathogenic | damaging | tolerated |
| ENST00000305208.11 | rs200370335 | R450H | deleterious - low confidence | probably damaging | medium | Disease | neutral | neutral | Benign | damaging | tolerated |
| ENST00000305208.11 | rs200370335 | R450L | deleterious - low confidence | probably damaging | high | Disease | Disease | Disease | Pathogenic | damaging | tolerated |
| ENST00000305208.11 | rs114982090 | P451L | deleterious - low confidence | probably damaging | medium | Disease | Disease | Disease | Pathogenic | damaging | tolerated |
| ENST00000305208.11 | rs587784536 | V452M | tolerated - low confidence | benign | neutral | neutral | neutral | neutral | Benign | Benign | tolerated |
| ENST00000305208.11 | rs587784536 | V452L | deleterious - low confidence | benign | low | neutral | neutral | neutral | Benign | Benign | tolerated |
| ENST00000305208.11 | rs1312404188 | E453Q | deleterious - low confidence | benign | medium | neutral | neutral | neutral | Benign | Benign | tolerated |
| ENST00000305208.11 | rs762488947 | P454L | deleterious - low confidence | probably damaging | high | Disease | Disease | Disease | Pathogenic | damaging | tolerated |
| ENST00000305208.11 | rs1700504653 | L455R | deleterious - low confidence | probably damaging | medium | Disease | neutral | Disease | Pathogenic | damaging | tolerated |
| ENST00000305208.11 | rs919221213 | D456Y | deleterious - low confidence | probably damaging | high | Disease | Disease | Disease | Pathogenic | damaging | tolerated |
| ENST00000305208.11 | rs1700505189 | D456V | deleterious - low confidence | probably damaging | high | Disease | Disease | Disease | Pathogenic | damaging | tolerated |
| ENST00000305208.11 | rs773957041 | D456E | deleterious - low confidence | possibly damaging | low | neutral | neutral | neutral | Benign | damaging | tolerated |
| ENST00000305208.11 | rs767264478 | L457M | deleterious | probably damaging | medium | neutral | neutral | neutral | Benign | damaging | tolerated |
| ENST00000305208.11 | rs527798161 | L457Q | deleterious | probably damaging | medium | Disease | neutral | neutral | Benign | damaging | tolerated |
| ENST00000305208.11 | rs1207971053 | A458T | deleterious | probably damaging | medium | Disease | Disease | Disease | Pathogenic | damaging | tolerated |
| ENST00000305208.11 | rs373052727 | A458V | deleterious | probably damaging | medium | neutral | neutral | neutral | Pathogenic | damaging | tolerated |
| ENST00000305208.11 | rs1201825340 | V459M | deleterious | probably damaging | medium | neutral | neutral | neutral | Benign | damaging | tolerated |
| ENST00000305208.11 | rs1201825340 | V459L | deleterious | probably damaging | medium | neutral | neutral | neutral | Benign | damaging | tolerated |
| ENST00000305208.11 | rs115410088 | F460L | deleterious | probably damaging | high | Disease | neutral | Disease | Pathogenic | Benign | tolerated |
| ENST00000305208.11 | rs1476500325 | W461R | deleterious | probably damaging | high | Disease | Disease | Disease | Pathogenic | damaging | tolerated |
| ENST00000305208.11 | rs1424603943 | W461S | deleterious | probably damaging | high | Disease | Disease | Disease | Pathogenic | damaging | tolerated |
| ENST00000305208.11 | rs753234232 | V462M | deleterious | probably damaging | medium | Disease | neutral | neutral | Benign | damaging | tolerated |
| ENST00000305208.11 | rs758454924 | E463K | deleterious | probably damaging | high | Disease | Disease | Disease | Pathogenic | damaging | tolerated |
| ENST00000305208.11 | rs72551358 | E463A | deleterious | probably damaging | high | Disease | Disease | Disease | Pathogenic | damaging | tolerated |
| ENST00000305208.11 | rs72551358 | E463G | deleterious | probably damaging | high | Disease | Disease | Disease | Pathogenic | damaging | tolerated |
| ENST00000305208.11 | rs72551358 | E463V | deleterious | probably damaging | high | Disease | Disease | Disease | Pathogenic | damaging | tolerated |
| ENST00000305208.11 | rs115944950 | E463D | deleterious | probably damaging | medium | Disease | neutral | neutral | Pathogenic | damaging | tolerated |
| ENST00000305208.11 | rs1700509286 | F464I | deleterious | probably damaging | high | Disease | neutral | Disease | Pathogenic | damaging | tolerated |
| ENST00000305208.11 | rs1700509872 | F464Y | tolerated | benign | neutral | neutral | neutral | neutral | Benign | damaging | tolerated |
| ENST00000305208.11 | rs781623948 | M466I | deleterious | probably damaging | low | neutral | neutral | neutral | Benign | damaging | tolerated |
| ENST00000305208.11 | rs745603293 | R467K | tolerated | possibly damaging | medium | Disease | neutral | neutral | Benign | damaging | tolerated |
| ENST00000305208.11 | rs1559420158 | H468D | deleterious | probably damaging | high | Disease | Disease | Disease | Pathogenic | damaging | tolerated |
| ENST00000305208.11 | rs1559420158 | H468Y | deleterious | probably damaging | medium | Disease | Disease | Disease | Pathogenic | damaging | tolerated |
| ENST00000305208.11 | rs1400939614 | K469Q | deleterious | probably damaging | medium | Disease | neutral | neutral | Benign | damaging | tolerated |
| ENST00000305208.11 | rs2126066747 | G470S | deleterious | probably damaging | high | Disease | Disease | Disease | Pathogenic | damaging | damaging |
| ENST00000305208.11 | rs1187321852 | G470D | deleterious | probably damaging | high | Disease | Disease | Disease | Pathogenic | damaging | damaging |
| ENST00000305208.11 | rs775532505 | A471T | deleterious | probably damaging | medium | Disease | Disease | Disease | Pathogenic | damaging | tolerated |
| ENST00000305208.11 | rs1333719360 | A471V | deleterious | probably damaging | high | Disease | neutral | Disease | Pathogenic | damaging | tolerated |
| ENST00000305208.11 | rs377565834 | P472S | deleterious | benign | low | neutral | neutral | neutral | Benign | damaging | tolerated |
| ENST00000305208.11 | rs773725816 | H473Y | deleterious | probably damaging | medium | Disease | Disease | Disease | Pathogenic | damaging | tolerated |
| ENST00000305208.11 | rs72551359 | L474M | deleterious - low confidence | probably damaging | high | neutral | neutral | neutral | Pathogenic | damaging | tolerated |
| ENST00000305208.11 | rs1700514037 | L474R | deleterious - low confidence | probably damaging | high | Disease | Disease | Disease | Pathogenic | damaging | tolerated |
| ENST00000305208.11 | rs566674185 | R475S | deleterious | probably damaging | high | Disease | Disease | Disease | Pathogenic | damaging | tolerated |
| ENST00000305208.11 | rs566674185 | R475C | deleterious | probably damaging | high | Disease | Disease | Disease | Pathogenic | damaging | tolerated |
| ENST00000305208.11 | rs150687296 | R475H | deleterious | probably damaging | medium | Disease | Disease | Disease | Pathogenic | damaging | tolerated |
| ENST00000305208.11 | rs765612353 | A477T | deleterious | probably damaging | medium | neutral | neutral | neutral | Pathogenic | damaging | tolerated |
| ENST00000305208.11 | rs765612353 | A477S | deleterious | probably damaging | medium | neutral | neutral | neutral | Pathogenic | damaging | tolerated |
| ENST00000305208.11 | rs1700515616 | A478D | deleterious | probably damaging | high | Disease | Disease | Disease | Pathogenic | damaging | tolerated |
| ENST00000305208.11 | rs753109787 | H479Y | deleterious | probably damaging | low | neutral | neutral | neutral | Pathogenic | damaging | tolerated |
| ENST00000305208.11 | rs369610863 | H479Q | deleterious | probably damaging | medium | neutral | neutral | neutral | Pathogenic | damaging | tolerated |
| ENST00000305208.11 | rs751579554 | D480N | deleterious | probably damaging | low | neutral | neutral | neutral | Pathogenic | damaging | tolerated |
| ENST00000305208.11 | rs1433976375 | L481F | deleterious | probably damaging | high | Disease | Disease | Disease | Pathogenic | damaging | tolerated |
| ENST00000305208.11 | rs1700516890 | T482S | deleterious | possibly damaging | low | neutral | neutral | neutral | Benign | Benign | tolerated |
| ENST00000305208.11 | rs1279219087 | T482I | deleterious | probably damaging | medium | neutral | neutral | neutral | Pathogenic | Benign | tolerated |
| ENST00000305208.11 | rs1176419046 | W483L | deleterious | probably damaging | medium | Disease | Disease | Disease | Pathogenic | damaging | tolerated |
| ENST00000305208.11 | rs770903084 | Y484H | deleterious | probably damaging | medium | Disease | Disease | neutral | Pathogenic | Benign | tolerated |
| ENST00000305208.11 | rs1700517999 | Y484S | deleterious | possibly damaging | high | Disease | Disease | Disease | Pathogenic | Benign | tolerated |
| ENST00000305208.11 | rs34993780 | Y486N | deleterious | probably damaging | high | Disease | Disease | Disease | Pathogenic | damaging | tolerated |
| ENST00000305208.11 | rs34993780 | Y486H | deleterious | probably damaging | medium | Disease | Disease | Disease | Pathogenic | damaging | tolerated |
| ENST00000305208.11 | rs34993780 | Y486D | deleterious | probably damaging | high | Disease | Disease | Disease | Pathogenic | damaging | tolerated |
| ENST00000305208.11 | rs371183955 | H487Y | deleterious | possibly damaging | medium | neutral | neutral | neutral | Pathogenic | damaging | tolerated |
| ENST00000305208.11 | rs1700519300 | H487R | deleterious | probably damaging | high | Disease | Disease | Disease | Pathogenic | damaging | tolerated |
| ENST00000305208.11 | rs72551360 | S488C | deleterious - low confidence | possibly damaging | medium | Disease | neutral | Disease | Pathogenic | damaging | tolerated |
| ENST00000305208.11 | rs72551360 | S488F | deleterious - low confidence | probably damaging | high | Disease | neutral | Disease | Pathogenic | damaging | tolerated |
| ENST00000305208.11 | rs1300515739 | D490A | deleterious - low confidence | probably damaging | high | Disease | Disease | Disease | Pathogenic | damaging | tolerated |
| ENST00000305208.11 | rs747543462 | V491M | deleterious - low confidence | probably damaging | high | Disease | Disease | Disease | Benign | damaging | tolerated |
| ENST00000305208.11 | rs2126066962 | I492V | deleterious - low confidence | possibly damaging | medium | neutral | neutral | neutral | Benign | damaging | tolerated |
| ENST00000305208.11 | rs771600393 | I492T | deleterious - low confidence | probably damaging | medium | Disease | neutral | neutral | Benign | damaging | tolerated |
| ENST00000305208.11 | rs1700521067 | G493D | deleterious - low confidence | probably damaging | high | Disease | Disease | Disease | Pathogenic | Benign | tolerated |
| ENST00000305208.11 | rs760289864 | V498I | tolerated - low confidence | benign | neutral | neutral | neutral | neutral | Benign | Benign | tolerated |
| ENST00000305208.11 | rs199723856 | V499M | deleterious - low confidence | probably damaging | medium | Disease | neutral | neutral | Benign | damaging | tolerated |
| ENST00000305208.11 | rs199723856 | V499L | deleterious - low confidence | benign | low | neutral | neutral | neutral | Benign | damaging | tolerated |
| ENST00000305208.11 | rs763440969 | L500P | deleterious - low confidence | probably damaging | high | Disease | Disease | Disease | Pathogenic | damaging | tolerated |
| ENST00000305208.11 | rs1268705566 | T501R | tolerated - low confidence | probably damaging | medium | Disease | Disease | Disease | Pathogenic | damaging | tolerated |
| ENST00000305208.11 | rs764439291 | V502M | deleterious - low confidence | probably damaging | medium | Disease | neutral | neutral | Benign | damaging | tolerated |
| ENST00000305208.11 | rs752117935 | V502A | tolerated - low confidence | benign | low | neutral | neutral | neutral | Benign | damaging | tolerated |
| ENST00000305208.11 | rs1575871358 | A503V | tolerated - low confidence | benign | neutral | neutral | neutral | neutral | Benign | Benign | tolerated |
| ENST00000305208.11 | rs761746377 | F504L | tolerated - low confidence | benign | low | neutral | neutral | neutral | Benign | damaging | tolerated |
| ENST00000305208.11 | rs767732319 | F504Y | deleterious - low confidence | probably damaging | medium | neutral | neutral | neutral | Benign | damaging | tolerated |
| ENST00000305208.11 | rs1372118451 | I505V | tolerated - low confidence | benign | low | neutral | neutral | neutral | Benign | damaging | tolerated |
| ENST00000305208.11 | rs1341191921 | I505T | deleterious - low confidence | possibly damaging | low | Disease | neutral | neutral | Benign | damaging | tolerated |
| ENST00000305208.11 | rs1575871447 | T506P | tolerated - low confidence | possibly damaging | medium | Disease | Disease | Disease | Benign | Benign | tolerated |
| ENST00000305208.11 | rs1575871447 | T506A | tolerated - low confidence | benign | neutral | neutral | neutral | neutral | Benign | Benign | tolerated |
| ENST00000305208.11 | rs1575871453 | T506I | tolerated - low confidence | benign | neutral | neutral | neutral | neutral | Benign | Benign | tolerated |
| ENST00000305208.11 | rs780493798 | C509G | deleterious - low confidence | benign | medium | Disease | Disease | Disease | Benign | Benign | tolerated |
| ENST00000305208.11 | rs1700526717 | C509Y | deleterious - low confidence | probably damaging | medium | Disease | Disease | Disease | Benign | Benign | tolerated |
| ENST00000305208.11 | rs1042709 | A511P | tolerated - low confidence | possibly damaging | medium | Disease | Disease | Disease | Benign | Benign | tolerated |
| ENST00000305208.11 | rs1700527426 | G513S | tolerated - low confidence | benign | low | neutral | neutral | neutral | Benign | Benign | tolerated |
| ENST00000305208.11 | rs1575871592 | Y514C | tolerated - low confidence | benign | neutral | neutral | neutral | neutral | Benign | Benign | tolerated |
| ENST00000305208.11 | rs867393133 | R515W | deleterious - low confidence | possibly damaging | medium | Disease | neutral | Disease | Benign | damaging | tolerated |
| ENST00000305208.11 | rs778667717 | R515Q | tolerated - low confidence | possibly damaging | low | Disease | neutral | Disease | Benign | damaging | tolerated |
| ENST00000305208.11 | rs778667717 | R515L | deleterious - low confidence | probably damaging | medium | Disease | Disease | Disease | Benign | damaging | tolerated |
| ENST00000305208.11 | rs1559420791 | K516T | deleterious - low confidence | probably damaging | high | Disease | Disease | Disease | Benign | damaging | tolerated |
| ENST00000305208.11 | rs772037816 | K516N | deleterious - low confidence | probably damaging | medium | Disease | Disease | neutral | Benign | damaging | tolerated |
| ENST00000305208.11 | rs1700529136 | C517Y | deleterious - low confidence | probably damaging | high | Disease | Disease | Disease | Benign | damaging | tolerated |
| ENST00000305208.11 | rs2126067193 | L518F | tolerated - low confidence | benign | neutral | neutral | neutral | neutral | Benign | Benign | tolerated |
| ENST00000305208.11 | rs1553624227 | G519R | tolerated - low confidence | benign | low | neutral | neutral | neutral | Benign | damaging | tolerated |
| ENST00000305208.11 | rs867885761 | G519E | deleterious - low confidence | probably damaging | medium | Disease | neutral | Disease | Benign | damaging | tolerated |
| ENST00000305208.11 | rs867885761 | G519A | deleterious - low confidence | possibly damaging | medium | neutral | neutral | neutral | Benign | damaging | tolerated |
| ENST00000305208.11 | rs553499095 | K520Q | deleterious - low confidence | probably damaging | medium | neutral | Disease | neutral | Benign | damaging | tolerated |
| ENST00000305208.11 | rs553499095 | K520E | deleterious - low confidence | possibly damaging | medium | Disease | Disease | Disease | Benign | damaging | tolerated |
| ENST00000305208.11 | rs1559420819 | K520R | deleterious - low confidence | benign | low | neutral | neutral | neutral | Benign | damaging | tolerated |
| ENST00000305208.11 | rs987494598 | K521N | deleterious - low confidence | benign | medium | neutral | Disease | neutral | Benign | damaging | tolerated |
| ENST00000305208.11 | rs746578451 | G522R | deleterious - low confidence | benign | low | neutral | neutral | neutral | Benign | damaging | tolerated |
| ENST00000305208.11 | rs1169660767 | G522A | deleterious - low confidence | benign | medium | neutral | neutral | neutral | Benign | damaging | tolerated |
| ENST00000305208.11 | rs577014868 | R523Q | tolerated - low confidence | benign | medium | neutral | neutral | neutral | Benign | Benign | tolerated |
| ENST00000305208.11 | rs769084242 | V524L | tolerated - low confidence | benign | low | neutral | neutral | neutral | Benign | Benign | tolerated |
| ENST00000305208.11 | rs1700533882 | K525R | tolerated - low confidence | benign | medium | neutral | neutral | neutral | Benign | damaging | tolerated |
| ENST00000305208.11 | rs1575871841 | A527P | tolerated - low confidence | benign | low | neutral | neutral | neutral | Benign | Benign | tolerated |
| ENST00000305208.11 | rs1575871841 | A527S | tolerated - low confidence | benign | neutral | neutral | neutral | neutral | Benign | Benign | tolerated |
| ENST00000305208.11 | rs1196632178 | K531R | deleterious - low confidence | possibly damaging | medium | neutral | Disease | neutral | Benign | damaging | tolerated |
| ENST00000305208.11 | rs768200668 | H533Y | deleterious - low confidence | possibly damaging | medium | neutral | Disease | neutral | Benign | damaging | tolerated |
| ENST00000305208.11 | rs1350310639 | H533P | deleterious - low confidence | possibly damaging | medium | neutral | Disease | neutral | Benign | damaging | tolerated |
| ENST00000305208.11 | rs1350310639 | H533R | deleterious - low confidence | possibly damaging | medium | neutral | Disease | neutral | Benign | damaging | tolerated |

| **Table S14. Raw predictions for all retrieved UGT1A4 SNPS from all utilized in-silico tools** | | | | | | | | | | | |
| --- | --- | --- | --- | --- | --- | --- | --- | --- | --- | --- | --- |
| **Transcript** | **Variant ID** | **Mutations** | **Sift class** | **Polyphen2 class** | **Mutation assessor class** | **PhD-SNP** | **SNAP** | **Meta-SNP** | **E-SNPs&GO** | **Panther** | **FATHMM** |
| ENST00000373409.8 | rs771343471 | A2V | tolerated - low confidence | benign | medium | neutral | neutral | neutral | benign | benign | tolerated |
| ENST00000373409.8 | rs137930413 | R3K | tolerated - low confidence | benign | neutral | neutral | neutral | neutral | benign | benign | tolerated |
| ENST00000373409.8 | rs137930413 | R3T | tolerated - low confidence | benign | neutral | neutral | neutral | neutral | benign | benign | tolerated |
| ENST00000373409.8 | rs1317647482 | G4E | deleterious - low confidence | probably damaging | medium | neutral | Disease | neutral | benign | benign | tolerated |
| ENST00000373409.8 | rs759514860 | L5I | tolerated - low confidence | benign | medium | neutral | neutral | neutral | benign | benign | tolerated |
| ENST00000373409.8 | rs770037031 | L5P | tolerated - low confidence | benign | medium | neutral | neutral | neutral | benign | benign | tolerated |
| ENST00000373409.8 | rs72551336 | Q6R | tolerated - low confidence | benign | low | neutral | neutral | neutral | benign | benign | tolerated |
| ENST00000373409.8 | rs529035115 | Q6H | tolerated - low confidence | benign | medium | neutral | neutral | neutral | benign | benign | tolerated |
| ENST00000373409.8 | rs1575522445 | V7G | tolerated - low confidence | benign | low | neutral | neutral | neutral | benign | benign | tolerated |
| ENST00000373409.8 | rs199761544 | P10L | tolerated - low confidence | benign | neutral | neutral | neutral | neutral | benign | benign | tolerated |
| ENST00000373409.8 | rs3892221 | R11W | tolerated - low confidence | benign | neutral | neutral | neutral | neutral | benign | benign | tolerated |
| ENST00000373409.8 | rs149314940 | R11Q | tolerated - low confidence | benign | neutral | neutral | neutral | neutral | benign | benign | tolerated |
| ENST00000373409.8 | rs1447098381 | L12V | tolerated - low confidence | possibly damaging | medium | neutral | neutral | neutral | benign | benign | tolerated |
| ENST00000373409.8 | rs1210101221 | A13T | tolerated - low confidence | benign | low | neutral | neutral | neutral | benign | benign | tolerated |
| ENST00000373409.8 | rs755547805 | T14I | tolerated - low confidence | benign | low | neutral | neutral | neutral | benign | benign | tolerated |
| ENST00000373409.8 | rs754634228 | G15V | tolerated - low confidence | possibly damaging | low | neutral | neutral | neutral | benign | benign | tolerated |
| ENST00000373409.8 | rs2125663221 | L16V | tolerated - low confidence | benign | medium | neutral | neutral | neutral | benign | damaging | tolerated |
| ENST00000373409.8 | rs2076692973 | L17P | tolerated - low confidence | probably damaging | medium | Disease | Disease | Disease | Pathogenic | damaging | tolerated |
| ENST00000373409.8 | rs139927449 | L18H | deleterious - low confidence | probably damaging | high | Disease | neutral | Disease | Pathogenic | damaging | tolerated |
| ENST00000373409.8 | rs139927449 | L18P | deleterious - low confidence | possibly damaging | medium | Disease | Disease | Disease | Pathogenic | damaging | tolerated |
| ENST00000373409.8 | rs1278012950 | S21T | tolerated - low confidence | benign | low | neutral | neutral | neutral | benign | benign | tolerated |
| ENST00000373409.8 | rs755101119 | V22F | tolerated - low confidence | benign | low | Disease | neutral | neutral | benign | benign | tolerated |
| ENST00000373409.8 | rs774756189 | V22A | tolerated - low confidence | benign | low | neutral | neutral | neutral | benign | benign | tolerated |
| ENST00000373409.8 | rs1440717664 | Q23R | tolerated - low confidence | benign | low | neutral | Disease | neutral | benign | benign | tolerated |
| ENST00000373409.8 | rs6755571 | P24T | tolerated - low confidence | benign | low | neutral | neutral | neutral | benign | damaging | tolerated |
| ENST00000373409.8 | rs6755571 | P24S | tolerated - low confidence | benign | neutral | neutral | neutral | neutral | benign | damaging | tolerated |
| ENST00000373409.8 | rs1334385335 | P24H | tolerated - low confidence | benign | medium | neutral | neutral | neutral | benign | damaging | tolerated |
| ENST00000373409.8 | rs1334385335 | P24R | tolerated - low confidence | benign | low | neutral | neutral | neutral | benign | damaging | tolerated |
| ENST00000373409.8 | rs1280624543 | W25R | tolerated - low confidence | benign | low | neutral | Disease | neutral | benign | benign | tolerated |
| ENST00000373409.8 | rs763201473 | A26S | tolerated - low confidence | possibly damaging | low | neutral | neutral | neutral | benign | benign | tolerated |
| ENST00000373409.8 | rs1667541971 | A26D | deleterious - low confidence | possibly damaging | medium | Disease | Disease | Disease | Pathogenic | benign | tolerated |
| ENST00000373409.8 | rs1667541971 | A26G | tolerated - low confidence | possibly damaging | low | neutral | neutral | neutral | benign | benign | tolerated |
| ENST00000373409.8 | rs2076697390 | E27A | tolerated - low confidence | possibly damaging | medium | neutral | neutral | neutral | benign | damaging | tolerated |
| ENST00000373409.8 | rs377204506 | E27D | tolerated - low confidence | possibly damaging | medium | neutral | neutral | neutral | benign | damaging | tolerated |
| ENST00000373409.8 | rs1018341076 | G29A | deleterious - low confidence | benign | low | neutral | neutral | neutral | benign | damaging | tolerated |
| ENST00000373409.8 | rs760663679 | K30N | deleterious - low confidence | benign | medium | neutral | neutral | Disease | benign | damaging | tolerated |
| ENST00000373409.8 | rs748436250 | V31L | tolerated - low confidence | probably damaging | neutral | neutral | neutral | neutral | benign | benign | tolerated |
| ENST00000373409.8 | rs373069908 | L32V | deleterious - low confidence | probably damaging | medium | neutral | neutral | Disease | Pathogenic | damaging | tolerated |
| ENST00000373409.8 | rs979373640 | L32S | deleterious - low confidence | probably damaging | high | Disease | Disease | Disease | Pathogenic | damaging | tolerated |
| ENST00000373409.8 | rs754582990 | V33M | deleterious - low confidence | probably damaging | high | neutral | Disease | Disease | benign | damaging | tolerated |
| ENST00000373409.8 | rs778450561 | V33A | deleterious - low confidence | probably damaging | medium | neutral | neutral | Disease | benign | damaging | tolerated |
| ENST00000373409.8 | rs778450561 | V33G | deleterious - low confidence | probably damaging | high | neutral | Disease | Disease | Pathogenic | damaging | tolerated |
| ENST00000373409.8 | rs2076700879 | P35T | deleterious - low confidence | probably damaging | high | Disease | Disease | Disease | Pathogenic | damaging | tolerated |
| ENST00000373409.8 | rs2076700879 | P35S | deleterious - low confidence | probably damaging | medium | Disease | neutral | neutral | benign | damaging | tolerated |
| ENST00000373409.8 | rs1174289899 | P35L | deleterious - low confidence | probably damaging | high | Disease | Disease | Disease | Pathogenic | damaging | tolerated |
| ENST00000373409.8 | rs1264796990 | T36A | tolerated - low confidence | benign | neutral | neutral | neutral | neutral | benign | benign | tolerated |
| ENST00000373409.8 | rs149779946 | T36I | tolerated - low confidence | benign | neutral | neutral | neutral | Disease | benign | benign | tolerated |
| ENST00000373409.8 | rs781665803 | G38S | deleterious - low confidence | probably damaging | medium | Disease | Disease | Disease | benign | damaging | tolerated |
| ENST00000373409.8 | rs377569610 | S39N | deleterious - low confidence | probably damaging | high | Disease | Disease | Disease | Pathogenic | damaging | tolerated |
| ENST00000373409.8 | rs780242275 | P40A | deleterious - low confidence | benign | low | neutral | Disease | Disease | Pathogenic | benign | tolerated |
| ENST00000373409.8 | rs149017068 | P40H | tolerated - low confidence | benign | neutral | neutral | neutral | neutral | benign | benign | tolerated |
| ENST00000373409.8 | rs149017068 | P40L | deleterious - low confidence | benign | neutral | neutral | Disease | Disease | Pathogenic | benign | tolerated |
| ENST00000373409.8 | rs553189135 | L42I | deleterious - low confidence | probably damaging | low | neutral | neutral | neutral | benign | damaging | tolerated |
| ENST00000373409.8 | rs553189135 | L42F | tolerated - low confidence | probably damaging | medium | neutral | neutral | neutral | benign | damaging | tolerated |
| ENST00000373409.8 | rs771749966 | S43R | deleterious - low confidence | probably damaging | high | neutral | Disease | Disease | Pathogenic | damaging | tolerated |
| ENST00000373409.8 | rs773069421 | M44I | deleterious - low confidence | benign | medium | neutral | neutral | Disease | benign | damaging | tolerated |
| ENST00000373409.8 | rs369434904 | R45W | deleterious - low confidence | benign | medium | Disease | neutral | Disease | benign | damaging | tolerated |
| ENST00000373409.8 | rs766332804 | R45Q | tolerated - low confidence | benign | medium | neutral | neutral | Disease | benign | damaging | tolerated |
| ENST00000373409.8 | rs2076705502 | A47S | deleterious - low confidence | benign | low | neutral | neutral | neutral | benign | benign | tolerated |
| ENST00000373409.8 | rs140860588 | A47V | tolerated - low confidence | benign | neutral | neutral | neutral | neutral | benign | benign | tolerated |
| ENST00000373409.8 | rs2011425 | L48M | deleterious - low confidence | possibly damaging | medium | neutral | neutral | neutral | benign | benign | tolerated |
| ENST00000373409.8 | rs2011425 | L48V | tolerated - low confidence | benign | neutral | neutral | neutral | neutral | benign | benign | tolerated |
| ENST00000373409.8 | rs138086321 | L48S | deleterious - low confidence | benign | low | neutral | neutral | Disease | benign | benign | tolerated |
| ENST00000373409.8 | rs182680267 | R49G | deleterious - low confidence | possibly damaging | medium | neutral | neutral | Disease | benign | benign | tolerated |
| ENST00000373409.8 | rs182680267 | R49W | deleterious - low confidence | probably damaging | medium | Disease | neutral | Disease | benign | benign | tolerated |
| ENST00000373409.8 | rs201968211 | R49Q | tolerated - low confidence | benign | neutral | neutral | neutral | neutral | benign | benign | tolerated |
| ENST00000373409.8 | rs201968211 | R49P | deleterious - low confidence | possibly damaging | medium | Disease | Disease | Disease | Pathogenic | benign | tolerated |
| ENST00000373409.8 | rs1423240253 | E50K | tolerated - low confidence | benign | medium | neutral | neutral | Disease | Pathogenic | benign | tolerated |
| ENST00000373409.8 | rs2076707871 | E50G | tolerated - low confidence | possibly damaging | medium | neutral | neutral | Disease | benign | benign | tolerated |
| ENST00000373409.8 | rs45510694 | E50D | tolerated - low confidence | benign | high | neutral | neutral | Disease | Pathogenic | benign | tolerated |
| ENST00000373409.8 | rs1342997030 | L51F | deleterious - low confidence | probably damaging | high | Disease | Disease | Disease | benign | damaging | damaging |
| ENST00000373409.8 | rs1346744859 | H52Y | tolerated - low confidence | benign | medium | neutral | neutral | Disease | benign | benign | tolerated |
| ENST00000373409.8 | rs930216391 | A53S | tolerated - low confidence | possibly damaging | low | neutral | neutral | neutral | benign | benign | tolerated |
| ENST00000373409.8 | rs200639166 | A53D | tolerated - low confidence | probably damaging | low | neutral | neutral | Disease | benign | benign | tolerated |
| ENST00000373409.8 | rs200639166 | A53G | tolerated - low confidence | probably damaging | medium | neutral | neutral | Disease | benign | benign | tolerated |
| ENST00000373409.8 | rs200639166 | A53V | tolerated - low confidence | probably damaging | medium | neutral | neutral | Disease | benign | benign | tolerated |
| ENST00000373409.8 | rs1226240136 | H56R | deleterious - low confidence | probably damaging | high | Disease | Disease | Disease | Pathogenic | damaging | tolerated |
| ENST00000373409.8 | rs144275831 | H56Q | deleterious - low confidence | probably damaging | high | Disease | Disease | Disease | Pathogenic | damaging | tolerated |
| ENST00000373409.8 | rs772339197 | Q57R | deleterious - low confidence | possibly damaging | medium | neutral | neutral | Disease | benign | benign | tolerated |
| ENST00000373409.8 | rs772339197 | Q57L | deleterious - low confidence | possibly damaging | medium | neutral | neutral | Disease | benign | benign | tolerated |
| ENST00000373409.8 | rs746825567 | A58S | tolerated - low confidence | benign | neutral | neutral | neutral | Disease | benign | benign | tolerated |
| ENST00000373409.8 | rs141408391 | A58E | deleterious - low confidence | benign | medium | Disease | neutral | Disease | Pathogenic | benign | tolerated |
| ENST00000373409.8 | rs141408391 | A58V | tolerated - low confidence | benign | neutral | neutral | neutral | neutral | benign | benign | tolerated |
| ENST00000373409.8 | rs759529745 | V60L | deleterious - low confidence | possibly damaging | medium | Disease | neutral | Disease | benign | damaging | tolerated |
| ENST00000373409.8 | rs759529745 | V60F | deleterious - low confidence | probably damaging | medium | Disease | neutral | Disease | benign | damaging | tolerated |
| ENST00000373409.8 | rs2125665099 | V60A | tolerated - low confidence | benign | high | Disease | Disease | Disease | benign | damaging | tolerated |
| ENST00000373409.8 | rs199607987 | L61F | deleterious - low confidence | probably damaging | medium | Disease | Disease | Disease | benign | benign | tolerated |
| ENST00000373409.8 | rs775074896 | P63L | deleterious - low confidence | benign | medium | Disease | Disease | Disease | Pathogenic | damaging | tolerated |
| ENST00000373409.8 | rs763830494 | E64D | tolerated - low confidence | possibly damaging | low | neutral | neutral | Disease | benign | damaging | tolerated |
| ENST00000373409.8 | rs1443134850 | V65M | deleterious - low confidence | possibly damaging | medium | neutral | neutral | Disease | benign | damaging | tolerated |
| ENST00000373409.8 | rs1575524133 | V65G | deleterious - low confidence | possibly damaging | low | neutral | neutral | Disease | benign | damaging | tolerated |
| ENST00000373409.8 | rs1575524150 | M67V | tolerated - low confidence | benign | neutral | neutral | neutral | neutral | benign | benign | tolerated |
| ENST00000373409.8 | rs751307422 | M67I | tolerated - low confidence | benign | neutral | neutral | neutral | neutral | benign | benign | tolerated |
| ENST00000373409.8 | rs45621441 | H68Y | tolerated - low confidence | benign | neutral | neutral | neutral | neutral | benign | benign | tolerated |
| ENST00000373409.8 | rs1032757626 | K70E | tolerated - low confidence | probably damaging | medium | neutral | neutral | Disease | benign | damaging | tolerated |
| ENST00000373409.8 | rs140140394 | E71Q | tolerated - low confidence | benign | medium | neutral | neutral | Disease | benign | damaging | tolerated |
| ENST00000373409.8 | rs754383567 | E71D | tolerated - low confidence | benign | medium | neutral | neutral | Disease | benign | damaging | tolerated |
| ENST00000373409.8 | rs148862762 | E72D | tolerated - low confidence | benign | low | neutral | neutral | Disease | benign | benign | tolerated |
| ENST00000373409.8 | rs1297848565 | K73E | tolerated - low confidence | benign | neutral | Disease | neutral | Disease | benign | benign | tolerated |
| ENST00000373409.8 | rs201935850 | K73N | tolerated - low confidence | benign | neutral | neutral | neutral | Disease | benign | benign | tolerated |
| ENST00000373409.8 | rs1243491139 | F74L | tolerated - low confidence | possibly damaging | low | neutral | neutral | Disease | benign | damaging | tolerated |
| ENST00000373409.8 | rs2076716677 | F75S | deleterious - low confidence | probably damaging | medium | Disease | neutral | neutral | Pathogenic | benign | tolerated |
| ENST00000373409.8 | rs2076716677 | F75C | tolerated - low confidence | probably damaging | medium | Disease | neutral | Disease | Pathogenic | benign | tolerated |
| ENST00000373409.8 | rs1314129216 | T76P | deleterious - low confidence | probably damaging | medium | Disease | neutral | Disease | benign | damaging | tolerated |
| ENST00000373409.8 | rs1314129216 | T76S | deleterious - low confidence | probably damaging | medium | neutral | neutral | Disease | benign | damaging | tolerated |
| ENST00000373409.8 | rs970652650 | T76I | tolerated - low confidence | probably damaging | medium | Disease | neutral | Disease | benign | damaging | tolerated |
| ENST00000373409.8 | rs544702876 | L77V | tolerated - low confidence | benign | low | neutral | neutral | neutral | benign | benign | tolerated |
| ENST00000373409.8 | rs1244840042 | L77P | deleterious - low confidence | probably damaging | medium | neutral | neutral | neutral | Pathogenic | benign | tolerated |
| ENST00000373409.8 | rs200903552 | T78P | deleterious - low confidence | possibly damaging | neutral | Disease | neutral | Disease | benign | benign | tolerated |
| ENST00000373409.8 | rs200903552 | T78A | deleterious - low confidence | benign | low | neutral | neutral | neutral | benign | benign | tolerated |
| ENST00000373409.8 | rs200903552 | T78S | deleterious - low confidence | benign | neutral | neutral | neutral | Disease | benign | benign | tolerated |
| ENST00000373409.8 | rs1338312506 | T78R | tolerated - low confidence | benign | neutral | Disease | neutral | neutral | benign | benign | tolerated |
| ENST00000373409.8 | rs150934066 | A79T | tolerated - low confidence | benign | neutral | neutral | neutral | neutral | benign | benign | tolerated |
| ENST00000373409.8 | rs772884705 | A79D | deleterious - low confidence | benign | neutral | neutral | neutral | neutral | benign | benign | tolerated |
| ENST00000373409.8 | rs747301276 | Y80C | deleterious - low confidence | probably damaging | high | Disease | neutral | Disease | Pathogenic | benign | tolerated |
| ENST00000373409.8 | rs193183920 | A81T | deleterious - low confidence | benign | neutral | neutral | neutral | neutral | benign | benign | tolerated |
| ENST00000373409.8 | rs1575524665 | A81V | deleterious - low confidence | benign | low | neutral | neutral | neutral | benign | benign | tolerated |
| ENST00000373409.8 | rs745742855 | V82I | tolerated - low confidence | benign | low | neutral | neutral | neutral | benign | benign | tolerated |
| ENST00000373409.8 | rs745742855 | V82L | deleterious - low confidence | benign | medium | neutral | neutral | Disease | benign | benign | tolerated |
| ENST00000373409.8 | rs769802516 | P83S | tolerated - low confidence | benign | low | neutral | neutral | Disease | benign | benign | tolerated |
| ENST00000373409.8 | rs1363775237 | P83R | deleterious - low confidence | possibly damaging | medium | Disease | neutral | Disease | benign | benign | tolerated |
| ENST00000373409.8 | rs144655870 | W84R | deleterious - low confidence | benign | neutral | neutral | neutral | Disease | Pathogenic | benign | tolerated |
| ENST00000373409.8 | rs144655870 | W84G | deleterious - low confidence | benign | neutral | neutral | neutral | Disease | Pathogenic | benign | tolerated |
| ENST00000373409.8 | rs148007151 | W84C | deleterious - low confidence | benign | neutral | neutral | neutral | Disease | benign | benign | tolerated |
| ENST00000373409.8 | rs1195063088 | T85P | deleterious - low confidence | probably damaging | medium | Disease | neutral | Disease | benign | benign | tolerated |
| ENST00000373409.8 | rs2076722760 | Q86K | tolerated - low confidence | benign | neutral | neutral | neutral | neutral | benign | benign | tolerated |
| ENST00000373409.8 | rs1450020479 | Q86R | tolerated - low confidence | benign | neutral | neutral | neutral | Disease | benign | benign | tolerated |
| ENST00000373409.8 | rs143031439 | K87E | tolerated - low confidence | benign | neutral | neutral | neutral | neutral | benign | benign | tolerated |
| ENST00000373409.8 | rs1329829423 | K87M | deleterious - low confidence | benign | low | neutral | neutral | Disease | benign | benign | tolerated |
| ENST00000373409.8 | rs146146688 | K87N | deleterious - low confidence | benign | low | neutral | neutral | neutral | benign | benign | tolerated |
| ENST00000373409.8 | rs374586565 | F89S | deleterious - low confidence | benign | medium | Disease | neutral | neutral | Pathogenic | benign | tolerated |
| ENST00000373409.8 | rs2076724106 | D90N | tolerated - low confidence | benign | neutral | neutral | neutral | Disease | benign | benign | tolerated |
| ENST00000373409.8 | rs2076724278 | D90G | deleterious - low confidence | benign | low | neutral | neutral | Disease | benign | benign | tolerated |
| ENST00000373409.8 | rs183802414 | R91C | deleterious - low confidence | possibly damaging | low | Disease | neutral | Disease | benign | benign | tolerated |
| ENST00000373409.8 | rs570129053 | R91H | tolerated - low confidence | benign | neutral | neutral | neutral | Disease | benign | benign | tolerated |
| ENST00000373409.8 | rs376370143 | V92I | tolerated - low confidence | benign | neutral | neutral | neutral | neutral | benign | benign | tolerated |
| ENST00000373409.8 | rs376370143 | V92L | tolerated - low confidence | benign | neutral | neutral | neutral | neutral | benign | benign | tolerated |
| ENST00000373409.8 | rs780928839 | T93A | deleterious - low confidence | benign | neutral | neutral | neutral | neutral | benign | benign | tolerated |
| ENST00000373409.8 | rs780928839 | T93S | deleterious - low confidence | benign | neutral | neutral | neutral | neutral | benign | benign | tolerated |
| ENST00000373409.8 | rs188914242 | T93K | tolerated - low confidence | benign | neutral | neutral | neutral | Disease | benign | benign | tolerated |
| ENST00000373409.8 | rs188914242 | T93M | tolerated - low confidence | benign | neutral | neutral | neutral | neutral | benign | benign | tolerated |
| ENST00000373409.8 | rs780011480 | L94M | tolerated - low confidence | benign | neutral | neutral | neutral | neutral | benign | benign | tolerated |
| ENST00000373409.8 | rs780011480 | L94V | tolerated - low confidence | benign | neutral | neutral | neutral | neutral | benign | benign | tolerated |
| ENST00000373409.8 | rs766709379 | Y96H | tolerated - low confidence | benign | neutral | neutral | neutral | Disease | benign | benign | tolerated |
| ENST00000373409.8 | rs1473237971 | T97A | tolerated - low confidence | benign | neutral | neutral | neutral | neutral | benign | benign | tolerated |
| ENST00000373409.8 | rs2076727288 | T97I | tolerated - low confidence | benign | neutral | neutral | neutral | neutral | benign | benign | tolerated |
| ENST00000373409.8 | rs774010631 | G99R | tolerated - low confidence | benign | low | Disease | neutral | neutral | benign | benign | tolerated |
| ENST00000373409.8 | rs774010631 | G99W | tolerated - low confidence | benign | medium | Disease | Disease | Disease | benign | benign | tolerated |
| ENST00000373409.8 | rs2076727897 | G99A | tolerated - low confidence | benign | neutral | neutral | neutral | neutral | benign | benign | tolerated |
| ENST00000373409.8 | rs1175236836 | F100L | tolerated - low confidence | benign | neutral | neutral | neutral | Disease | benign | benign | tolerated |
| ENST00000373409.8 | rs1175236836 | F100V | tolerated - low confidence | benign | neutral | neutral | neutral | Disease | benign | benign | tolerated |
| ENST00000373409.8 | rs2076728564 | E102G | deleterious - low confidence | possibly damaging | low | neutral | neutral | Disease | benign | damaging | tolerated |
| ENST00000373409.8 | rs771877893 | E102D | tolerated - low confidence | benign | low | neutral | neutral | Disease | benign | damaging | tolerated |
| ENST00000373409.8 | rs2076729174 | E104K | tolerated - low confidence | benign | neutral | neutral | neutral | Disease | benign | benign | tolerated |
| ENST00000373409.8 | rs373602050 | E104D | tolerated - low confidence | benign | neutral | neutral | neutral | neutral | benign | benign | tolerated |
| ENST00000373409.8 | rs566867363 | H105R | tolerated - low confidence | benign | low | neutral | neutral | neutral | benign | benign | tolerated |
| ENST00000373409.8 | rs1671719594 | H105Q | tolerated - low confidence | benign | low | neutral | neutral | neutral | benign | benign | tolerated |
| ENST00000373409.8 | rs2076730014 | L106F | tolerated - low confidence | benign | neutral | neutral | neutral | neutral | benign | benign | tolerated |
| ENST00000373409.8 | rs1197566998 | L106R | deleterious - low confidence | benign | low | Disease | neutral | Disease | benign | benign | tolerated |
| ENST00000373409.8 | rs200858079 | L107Q | deleterious - low confidence | probably damaging | medium | Disease | neutral | Disease | benign | damaging | tolerated |
| ENST00000373409.8 | rs200858079 | L107P | deleterious - low confidence | probably damaging | medium | Disease | neutral | Disease | Pathogenic | damaging | tolerated |
| ENST00000373409.8 | rs539093785 | R109G | tolerated - low confidence | benign | low | Disease | neutral | Disease | benign | benign | tolerated |
| ENST00000373409.8 | rs763386476 | Y110H | tolerated - low confidence | benign | medium | neutral | neutral | Disease | benign | benign | tolerated |
| ENST00000373409.8 | rs764097812 | R112G | tolerated - low confidence | benign | neutral | neutral | neutral | Disease | benign | benign | tolerated |
| ENST00000373409.8 | rs751674220 | R112S | tolerated - low confidence | benign | neutral | neutral | neutral | Disease | benign | benign | tolerated |
| ENST00000373409.8 | rs757422133 | S113R | tolerated - low confidence | benign | low | neutral | neutral | Disease | benign | benign | tolerated |
| ENST00000373409.8 | rs757422133 | S113G | tolerated - low confidence | benign | medium | neutral | neutral | neutral | benign | benign | tolerated |
| ENST00000373409.8 | rs2125666938 | M114V | tolerated - low confidence | benign | low | neutral | neutral | Disease | benign | benign | tolerated |
| ENST00000373409.8 | rs2076731921 | M114K | tolerated - low confidence | benign | low | Disease | Disease | Disease | benign | benign | tolerated |
| ENST00000373409.8 | rs1279561037 | M114I | tolerated - low confidence | benign | neutral | neutral | neutral | Disease | benign | benign | tolerated |
| ENST00000373409.8 | rs2125666999 | A115P | tolerated - low confidence | benign | medium | Disease | neutral | Disease | benign | benign | tolerated |
| ENST00000373409.8 | rs569258363 | I116V | tolerated - low confidence | benign | neutral | neutral | neutral | neutral | benign | benign | tolerated |
| ENST00000373409.8 | rs1202848834 | I116M | tolerated - low confidence | benign | neutral | neutral | neutral | neutral | benign | benign | tolerated |
| ENST00000373409.8 | rs1251118091 | M117L | tolerated - low confidence | benign | neutral | neutral | neutral | Disease | benign | benign | tolerated |
| ENST00000373409.8 | rs2076733024 | M117T | tolerated - low confidence | benign | neutral | neutral | neutral | Disease | benign | benign | tolerated |
| ENST00000373409.8 | rs867842846 | M117I | tolerated - low confidence | benign | neutral | neutral | neutral | Disease | benign | benign | tolerated |
| ENST00000373409.8 | rs1217144051 | N118K | tolerated - low confidence | benign | neutral | neutral | neutral | Disease | benign | benign | tolerated |
| ENST00000373409.8 | rs538162022 | N119S | tolerated - low confidence | benign | low | neutral | neutral | Disease | benign | benign | tolerated |
| ENST00000373409.8 | rs749190317 | V120I | tolerated - low confidence | benign | neutral | neutral | neutral | neutral | benign | benign | tolerated |
| ENST00000373409.8 | rs749190317 | V120L | tolerated - low confidence | benign | neutral | neutral | neutral | neutral | benign | benign | tolerated |
| ENST00000373409.8 | rs2076734204 | V120A | tolerated - low confidence | benign | neutral | neutral | neutral | Disease | benign | benign | tolerated |
| ENST00000373409.8 | rs2076734729 | S121P | deleterious - low confidence | possibly damaging | medium | Disease | neutral | Disease | Pathogenic | benign | tolerated |
| ENST00000373409.8 | rs747678615 | A123T | tolerated - low confidence | benign | neutral | neutral | neutral | neutral | benign | benign | tolerated |
| ENST00000373409.8 | rs771753301 | A123V | tolerated - low confidence | benign | neutral | neutral | neutral | neutral | benign | benign | tolerated |
| ENST00000373409.8 | rs772886660 | L124I | tolerated - low confidence | benign | low | neutral | neutral | neutral | benign | benign | tolerated |
| ENST00000373409.8 | rs772886660 | L124F | tolerated - low confidence | benign | neutral | neutral | neutral | neutral | benign | benign | tolerated |
| ENST00000373409.8 | rs1353737147 | L124H | tolerated - low confidence | benign | low | Disease | neutral | Disease | Pathogenic | benign | tolerated |
| ENST00000373409.8 | rs1343607687 | R126W | deleterious - low confidence | probably damaging | medium | Disease | Disease | Disease | benign | benign | tolerated |
| ENST00000373409.8 | rs147555582 | C127S | tolerated - low confidence | benign | neutral | neutral | neutral | neutral | benign | benign | tolerated |
| ENST00000373409.8 | rs764611570 | C128G | deleterious - low confidence | probably damaging | medium | Disease | Disease | Disease | Pathogenic | damaging | tolerated |
| ENST00000373409.8 | rs145806554 | C128Y | deleterious - low confidence | probably damaging | high | Disease | Disease | Disease | Pathogenic | damaging | tolerated |
| ENST00000373409.8 | rs145806554 | C128F | deleterious - low confidence | probably damaging | high | Disease | Disease | Disease | benign | damaging | tolerated |
| ENST00000373409.8 | rs2076737116 | V129L | tolerated - low confidence | benign | low | neutral | neutral | neutral | benign | benign | tolerated |
| ENST00000373409.8 | rs2076737405 | E130K | tolerated - low confidence | benign | medium | neutral | neutral | Disease | benign | benign | tolerated |
| ENST00000373409.8 | rs1327237440 | L131P | deleterious - low confidence | probably damaging | high | Disease | Disease | Disease | Pathogenic | benign | tolerated |
| ENST00000373409.8 | rs72551337 | L132P | deleterious - low confidence | probably damaging | high | Disease | Disease | Disease | Pathogenic | damaging | tolerated |
| ENST00000373409.8 | rs1235910435 | H133Y | tolerated - low confidence | benign | neutral | neutral | neutral | Disease | benign | benign | tolerated |
| ENST00000373409.8 | rs1197156994 | H133P | deleterious - low confidence | possibly damaging | medium | Disease | neutral | Disease | Pathogenic | benign | tolerated |
| ENST00000373409.8 | rs2076738456 | E135G | deleterious - low confidence | benign | low | neutral | neutral | Disease | benign | benign | tolerated |
| ENST00000373409.8 | rs2076738991 | A136T | tolerated - low confidence | benign | neutral | neutral | neutral | neutral | benign | benign | tolerated |
| ENST00000373409.8 | rs2076738991 | A136S | tolerated - low confidence | benign | neutral | neutral | neutral | neutral | benign | benign | tolerated |
| ENST00000373409.8 | rs1490347566 | I138V | tolerated - low confidence | benign | low | neutral | neutral | neutral | benign | benign | tolerated |
| ENST00000373409.8 | rs766129715 | R139T | tolerated - low confidence | benign | neutral | neutral | neutral | Disease | benign | benign | tolerated |
| ENST00000373409.8 | rs1184427217 | L141R | deleterious - low confidence | probably damaging | high | Disease | Disease | Disease | Pathogenic | damaging | tolerated |
| ENST00000373409.8 | rs375836466 | N142H | deleterious - low confidence | benign | low | neutral | neutral | Disease | benign | benign | tolerated |
| ENST00000373409.8 | rs748132816 | A143V | deleterious - low confidence | possibly damaging | medium | neutral | neutral | Disease | benign | benign | tolerated |
| ENST00000373409.8 | rs757974973 | S145T | tolerated - low confidence | possibly damaging | medium | neutral | neutral | neutral | benign | benign | tolerated |
| ENST00000373409.8 | rs199688431 | S145Y | deleterious - low confidence | possibly damaging | medium | Disease | neutral | Disease | benign | benign | tolerated |
| ENST00000373409.8 | rs199688431 | S145C | deleterious - low confidence | probably damaging | medium | Disease | neutral | Disease | benign | benign | tolerated |
| ENST00000373409.8 | rs199688431 | S145F | deleterious - low confidence | benign | medium | Disease | neutral | Disease | benign | benign | tolerated |
| ENST00000373409.8 | rs746608425 | D147N | deleterious - low confidence | probably damaging | medium | Disease | neutral | Disease | benign | damaging | tolerated |
| ENST00000373409.8 | rs775879041 | L150F | tolerated - low confidence | benign | low | neutral | neutral | Disease | benign | benign | tolerated |
| ENST00000373409.8 | rs1269795663 | T151I | deleterious - low confidence | benign | medium | neutral | neutral | Disease | benign | damaging | tolerated |
| ENST00000373409.8 | rs749630489 | D152E | deleterious - low confidence | probably damaging | medium | neutral | Disease | Disease | Pathogenic | damaging | tolerated |
| ENST00000373409.8 | rs1231059652 | P153S | deleterious - low confidence | probably damaging | medium | Disease | neutral | Disease | Pathogenic | damaging | tolerated |
| ENST00000373409.8 | rs769049134 | P153L | deleterious - low confidence | probably damaging | high | Disease | neutral | Disease | Pathogenic | damaging | tolerated |
| ENST00000373409.8 | rs762367100 | V154I | tolerated - low confidence | benign | low | neutral | neutral | neutral | benign | benign | tolerated |
| ENST00000373409.8 | rs762367100 | V154F | tolerated - low confidence | benign | neutral | neutral | neutral | Disease | benign | benign | tolerated |
| ENST00000373409.8 | rs773168968 | V154D | deleterious - low confidence | possibly damaging | medium | Disease | neutral | Disease | benign | benign | tolerated |
| ENST00000373409.8 | rs760908491 | N155H | tolerated - low confidence | benign | low | neutral | neutral | Disease | benign | benign | tolerated |
| ENST00000373409.8 | rs1158648938 | N155S | tolerated - low confidence | benign | neutral | neutral | neutral | neutral | benign | benign | tolerated |
| ENST00000373409.8 | rs766528194 | N155K | tolerated - low confidence | benign | low | neutral | neutral | Disease | Pathogenic | benign | tolerated |
| ENST00000373409.8 | rs1465114425 | L156I | deleterious - low confidence | benign | neutral | neutral | neutral | neutral | benign | benign | tolerated |
| ENST00000373409.8 | rs1465114425 | L156V | tolerated - low confidence | benign | neutral | neutral | neutral | neutral | benign | benign | tolerated |
| ENST00000373409.8 | rs2011404 | C157W | deleterious - low confidence | probably damaging | high | Disease | Disease | Disease | Pathogenic | damaging | tolerated |
| ENST00000373409.8 | rs146073833 | G158R | deleterious - low confidence | probably damaging | high | Disease | Disease | Disease | Pathogenic | benign | tolerated |
| ENST00000373409.8 | rs149433426 | G158E | deleterious - low confidence | possibly damaging | high | Disease | Disease | Disease | Pathogenic | benign | tolerated |
| ENST00000373409.8 | rs149433426 | G158A | deleterious - low confidence | benign | medium | Disease | neutral | Disease | benign | benign | tolerated |
| ENST00000373409.8 | rs149433426 | G158V | deleterious - low confidence | benign | high | Disease | neutral | Disease | Pathogenic | benign | tolerated |
| ENST00000373409.8 | rs148565852 | A159V | tolerated - low confidence | benign | low | neutral | neutral | neutral | benign | benign | tolerated |
| ENST00000373409.8 | rs1399021133 | V160M | deleterious - low confidence | possibly damaging | low | neutral | neutral | Disease | benign | benign | tolerated |
| ENST00000373409.8 | rs2076749752 | L161V | tolerated - low confidence | benign | neutral | neutral | neutral | neutral | benign | benign | tolerated |
| ENST00000373409.8 | rs2076749909 | L161P | deleterious - low confidence | probably damaging | medium | Disease | Disease | Disease | Pathogenic | benign | tolerated |
| ENST00000373409.8 | rs1283933646 | A162T | deleterious - low confidence | probably damaging | high | Disease | neutral | Disease | Pathogenic | damaging | tolerated |
| ENST00000373409.8 | rs149960993 | A162V | deleterious - low confidence | probably damaging | high | Disease | neutral | Disease | Pathogenic | damaging | tolerated |
| ENST00000373409.8 | rs780682162 | K163N | deleterious - low confidence | possibly damaging | low | neutral | neutral | Disease | benign | benign | tolerated |
| ENST00000373409.8 | rs2076750903 | Y164D | deleterious - low confidence | probably damaging | high | Disease | neutral | Disease | Pathogenic | damaging | tolerated |
| ENST00000373409.8 | rs149079663 | S166L | deleterious - low confidence | possibly damaging | medium | Disease | neutral | Disease | benign | damaging | tolerated |
| ENST00000373409.8 | rs1246045139 | I167T | deleterious - low confidence | possibly damaging | medium | Disease | neutral | Disease | benign | benign | tolerated |
| ENST00000373409.8 | rs773289420 | P168S | deleterious - low confidence | probably damaging | high | Disease | Disease | Disease | Pathogenic | damaging | tolerated |
| ENST00000373409.8 | rs760645007 | P168R | deleterious - low confidence | probably damaging | high | Disease | Disease | Disease | Pathogenic | damaging | tolerated |
| ENST00000373409.8 | rs771082212 | A169T | tolerated - low confidence | benign | low | neutral | neutral | neutral | benign | damaging | tolerated |
| ENST00000373409.8 | rs771082212 | A169P | deleterious - low confidence | possibly damaging | medium | Disease | neutral | Disease | Pathogenic | damaging | tolerated |
| ENST00000373409.8 | rs776987422 | V170M | deleterious - low confidence | probably damaging | medium | Disease | neutral | Disease | Pathogenic | damaging | tolerated |
| ENST00000373409.8 | rs776987422 | V170L | deleterious - low confidence | probably damaging | medium | Disease | neutral | Disease | Pathogenic | damaging | tolerated |
| ENST00000373409.8 | rs1210243448 | F172L | tolerated - low confidence | benign | medium | neutral | neutral | neutral | Pathogenic | damaging | tolerated |
| ENST00000373409.8 | rs759894236 | F172S | deleterious - low confidence | probably damaging | low | neutral | neutral | Disease | benign | damaging | tolerated |
| ENST00000373409.8 | rs759894236 | F172C | deleterious - low confidence | probably damaging | high | Disease | neutral | Disease | Pathogenic | damaging | tolerated |
| ENST00000373409.8 | rs546499527 | W173L | tolerated - low confidence | benign | neutral | neutral | neutral | neutral | benign | benign | tolerated |
| ENST00000373409.8 | rs188312736 | R174K | deleterious - low confidence | possibly damaging | medium | Disease | Disease | Disease | Pathogenic | damaging | tolerated |
| ENST00000373409.8 | rs1239122112 | R174S | deleterious - low confidence | possibly damaging | medium | neutral | Disease | Disease | Pathogenic | damaging | tolerated |
| ENST00000373409.8 | rs532530633 | Y175N | deleterious - low confidence | benign | low | Disease | neutral | neutral | benign | benign | tolerated |
| ENST00000373409.8 | rs45540231 | I176L | tolerated - low confidence | benign | neutral | neutral | Disease | Disease | benign | benign | tolerated |
| ENST00000373409.8 | rs45540231 | I176F | tolerated - low confidence | possibly damaging | low | neutral | neutral | neutral | benign | benign | tolerated |
| ENST00000373409.8 | rs2076756865 | P177S | deleterious - low confidence | benign | medium | Disease | neutral | Disease | benign | damaging | tolerated |
| ENST00000373409.8 | rs2076757024 | C178S | deleterious - low confidence | possibly damaging | medium | neutral | neutral | Disease | Pathogenic | damaging | tolerated |
| ENST00000373409.8 | rs751560022 | D179V | deleterious - low confidence | probably damaging | low | neutral | neutral | Disease | benign | benign | tolerated |
| ENST00000373409.8 | rs1401813450 | D181N | deleterious - low confidence | probably damaging | medium | neutral | neutral | neutral | benign | damaging | tolerated |
| ENST00000373409.8 | rs780815803 | D181G | deleterious - low confidence | possibly damaging | medium | Disease | Disease | Disease | Pathogenic | damaging | tolerated |
| ENST00000373409.8 | rs569317540 | D181E | tolerated - low confidence | benign | neutral | neutral | neutral | neutral | benign | damaging | tolerated |
| ENST00000373409.8 | rs779205273 | K183N | deleterious - low confidence | possibly damaging | low | Disease | neutral | Disease | benign | benign | tolerated |
| ENST00000373409.8 | rs1322563155 | G184S | tolerated - low confidence | benign | neutral | neutral | neutral | neutral | benign | benign | tolerated |
| ENST00000373409.8 | rs772532709 | T185A | tolerated - low confidence | benign | low | neutral | neutral | neutral | benign | damaging | tolerated |
| ENST00000373409.8 | rs2076759455 | T185I | deleterious - low confidence | possibly damaging | medium | neutral | neutral | neutral | Pathogenic | damaging | tolerated |
| ENST00000373409.8 | rs2076759627 | C187Y | deleterious - low confidence | probably damaging | high | neutral | neutral | Disease | Pathogenic | damaging | tolerated |
| ENST00000373409.8 | rs778111484 | P188R | deleterious - low confidence | probably damaging | high | Disease | Disease | Disease | Pathogenic | damaging | tolerated |
| ENST00000373409.8 | rs2076760173 | N189D | tolerated - low confidence | possibly damaging | medium | neutral | neutral | Disease | Pathogenic | benign | tolerated |
| ENST00000373409.8 | rs141300284 | N189S | tolerated - low confidence | benign | neutral | neutral | neutral | neutral | benign | damaging | tolerated |
| ENST00000373409.8 | rs1343929267 | P190H | deleterious - low confidence | probably damaging | high | Disease | Disease | Disease | Pathogenic | damaging | tolerated |
| ENST00000373409.8 | rs776862495 | S191F | tolerated - low confidence | benign | low | neutral | neutral | Disease | benign | benign | tolerated |
| ENST00000373409.8 | rs770164749 | S192Y | deleterious - low confidence | probably damaging | high | Disease | neutral | Disease | Pathogenic | damaging | tolerated |
| ENST00000373409.8 | rs2076761888 | Y193C | deleterious - low confidence | probably damaging | high | Disease | Disease | Disease | Pathogenic | damaging | tolerated |
| ENST00000373409.8 | rs2076762269 | P195L | deleterious - low confidence | probably damaging | high | Disease | neutral | Disease | Pathogenic | damaging | tolerated |
| ENST00000373409.8 | rs775904501 | K196R | tolerated - low confidence | benign | neutral | neutral | neutral | neutral | benign | benign | tolerated |
| ENST00000373409.8 | rs762624701 | K196N | deleterious - low confidence | benign | neutral | neutral | neutral | neutral | benign | benign | tolerated |
| ENST00000373409.8 | rs538242607 | T199M | deleterious - low confidence | probably damaging | medium | neutral | neutral | Disease | benign | benign | tolerated |
| ENST00000373409.8 | rs761827245 | T200N | tolerated - low confidence | benign | low | neutral | neutral | Disease | benign | benign | tolerated |
| ENST00000373409.8 | rs761827245 | T200I | tolerated - low confidence | benign | low | neutral | neutral | Disease | benign | damaging | tolerated |
| ENST00000373409.8 | rs1171776223 | N201H | tolerated - low confidence | benign | low | neutral | neutral | Disease | benign | damaging | tolerated |
| ENST00000373409.8 | rs1377267977 | N201S | tolerated - low confidence | possibly damaging | medium | neutral | neutral | neutral | benign | damaging | tolerated |
| ENST00000373409.8 | rs1477800386 | S202T | tolerated - low confidence | possibly damaging | low | neutral | neutral | neutral | benign | benign | tolerated |
| ENST00000373409.8 | rs766948934 | S202L | deleterious - low confidence | probably damaging | high | Disease | neutral | Disease | Pathogenic | benign | tolerated |
| ENST00000373409.8 | rs1422028440 | D203N | deleterious - low confidence | probably damaging | high | Disease | neutral | Disease | benign | damaging | tolerated |
| ENST00000373409.8 | rs2076765581 | M205I | deleterious - low confidence | probably damaging | high | Disease | Disease | Disease | Pathogenic | damaging | tolerated |
| ENST00000373409.8 | rs749964914 | T206A | deleterious - low confidence | possibly damaging | medium | neutral | neutral | Disease | benign | damaging | tolerated |
| ENST00000373409.8 | rs1189079913 | T206I | deleterious - low confidence | probably damaging | high | Disease | Disease | Disease | benign | damaging | tolerated |
| ENST00000373409.8 | rs2076766360 | F207L | deleterious - low confidence | probably damaging | medium | Disease | neutral | Disease | benign | damaging | tolerated |
| ENST00000373409.8 | rs2076766565 | F207C | deleterious - low confidence | probably damaging | high | Disease | Disease | Disease | Pathogenic | damaging | tolerated |
| ENST00000373409.8 | rs1309137091 | L208M | tolerated - low confidence | benign | low | neutral | neutral | neutral | benign | damaging | tolerated |
| ENST00000373409.8 | rs765990866 | Q209R | deleterious - low confidence | possibly damaging | high | Disease | Disease | Disease | benign | damaging | tolerated |
| ENST00000373409.8 | rs753525211 | R210K | deleterious - low confidence | probably damaging | high | Disease | Disease | Disease | Pathogenic | damaging | tolerated |
| ENST00000373409.8 | rs2076767946 | V211I | deleterious - low confidence | probably damaging | medium | neutral | neutral | Disease | benign | damaging | tolerated |
| ENST00000373409.8 | rs758684998 | N213K | deleterious - low confidence | probably damaging | high | Disease | Disease | Disease | Pathogenic | damaging | tolerated |
| ENST00000373409.8 | rs1246194509 | M214V | tolerated - low confidence | benign | low | neutral | neutral | neutral | benign | damaging | tolerated |
| ENST00000373409.8 | rs1246194509 | M214L | tolerated - low confidence | benign | medium | neutral | neutral | neutral | benign | damaging | tolerated |
| ENST00000373409.8 | rs1219690957 | M214T | tolerated - low confidence | possibly damaging | medium | neutral | neutral | Disease | benign | damaging | tolerated |
| ENST00000373409.8 | rs778257140 | L215V | deleterious - low confidence | probably damaging | medium | neutral | neutral | Disease | benign | damaging | tolerated |
| ENST00000373409.8 | rs778257140 | L215F | deleterious - low confidence | probably damaging | medium | neutral | neutral | Disease | benign | damaging | tolerated |
| ENST00000373409.8 | rs2076769813 | Y216H | tolerated - low confidence | probably damaging | medium | neutral | neutral | Disease | benign | benign | tolerated |
| ENST00000373409.8 | rs1275256399 | Y216S | tolerated - low confidence | probably damaging | low | neutral | neutral | neutral | benign | benign | tolerated |
| ENST00000373409.8 | rs1275256399 | Y216C | deleterious - low confidence | probably damaging | medium | Disease | neutral | Disease | benign | benign | tolerated |
| ENST00000373409.8 | rs568794982 | P217L | tolerated - low confidence | benign | low | neutral | neutral | neutral | benign | benign | tolerated |
| ENST00000373409.8 | rs2076770732 | A219T | tolerated - low confidence | benign | low | neutral | neutral | neutral | benign | benign | tolerated |
| ENST00000373409.8 | rs1220273239 | A219V | tolerated - low confidence | benign | neutral | neutral | neutral | neutral | benign | benign | tolerated |
| ENST00000373409.8 | rs2076771249 | L220P | deleterious - low confidence | benign | medium | Disease | neutral | Disease | Pathogenic | benign | tolerated |
| ENST00000373409.8 | rs138822211 | I223L | tolerated - low confidence | benign | neutral | neutral | neutral | neutral | benign | benign | tolerated |
| ENST00000373409.8 | rs138822211 | I223V | tolerated - low confidence | benign | neutral | neutral | neutral | neutral | benign | benign | tolerated |
| ENST00000373409.8 | rs775855260 | C224Y | deleterious - low confidence | probably damaging | medium | Disease | neutral | neutral | Pathogenic | damaging | tolerated |
| ENST00000373409.8 | rs554107751 | T226A | tolerated - low confidence | benign | neutral | neutral | neutral | neutral | benign | benign | tolerated |
| ENST00000373409.8 | rs774203287 | T226I | tolerated - low confidence | benign | neutral | neutral | neutral | neutral | benign | benign | tolerated |
| ENST00000373409.8 | rs761613256 | F227L | tolerated - low confidence | benign | neutral | neutral | neutral | neutral | benign | benign | tolerated |
| ENST00000373409.8 | rs761613256 | F227V | tolerated - low confidence | benign | neutral | neutral | neutral | neutral | benign | benign | tolerated |
| ENST00000373409.8 | rs1559364966 | A229S | tolerated - low confidence | benign | neutral | neutral | neutral | neutral | benign | benign | tolerated |
| ENST00000373409.8 | rs760142681 | P230S | tolerated - low confidence | benign | low | neutral | neutral | Disease | benign | damaging | tolerated |
| ENST00000373409.8 | rs372293131 | P230L | deleterious - low confidence | possibly damaging | medium | Disease | neutral | Disease | Pathogenic | damaging | tolerated |
| ENST00000373409.8 | rs753419237 | Y231H | deleterious - low confidence | probably damaging | medium | neutral | neutral | Disease | Pathogenic | damaging | tolerated |
| ENST00000373409.8 | rs754670633 | Y231F | tolerated - low confidence | benign | low | neutral | neutral | Disease | Pathogenic | damaging | tolerated |
| ENST00000373409.8 | rs1307171724 | A232P | tolerated - low confidence | benign | medium | neutral | neutral | Disease | Pathogenic | damaging | tolerated |
| ENST00000373409.8 | rs764297894 | A232E | tolerated - low confidence | benign | neutral | neutral | neutral | Disease | benign | damaging | tolerated |
| ENST00000373409.8 | rs764297894 | A232G | tolerated - low confidence | possibly damaging | low | neutral | neutral | Disease | benign | damaging | tolerated |
| ENST00000373409.8 | rs751977605 | S233N | tolerated - low confidence | benign | low | neutral | neutral | Disease | benign | benign | tolerated |
| ENST00000373409.8 | rs757746269 | S233R | tolerated - low confidence | benign | neutral | neutral | neutral | neutral | benign | benign | tolerated |
| ENST00000373409.8 | rs1307558571 | S236P | deleterious - low confidence | probably damaging | high | Disease | neutral | Disease | Pathogenic | damaging | tolerated |
| ENST00000373409.8 | rs2076777765 | E237K | deleterious - low confidence | benign | low | neutral | neutral | neutral | benign | damaging | tolerated |
| ENST00000373409.8 | rs377261801 | E237D | tolerated - low confidence | benign | medium | neutral | neutral | Disease | benign | damaging | tolerated |
| ENST00000373409.8 | rs2076778233 | L238P | deleterious - low confidence | probably damaging | medium | Disease | neutral | Disease | Pathogenic | benign | tolerated |
| ENST00000373409.8 | rs2076778402 | F239Y | deleterious - low confidence | possibly damaging | medium | neutral | neutral | Disease | Pathogenic | benign | tolerated |
| ENST00000373409.8 | rs746444370 | F239L | tolerated - low confidence | benign | neutral | neutral | neutral | neutral | benign | benign | tolerated |
| ENST00000373409.8 | rs1488835292 | Q240H | tolerated - low confidence | possibly damaging | medium | neutral | Disease | Disease | benign | damaging | tolerated |
| ENST00000373409.8 | rs1282092196 | E242Q | deleterious - low confidence | benign | low | neutral | neutral | neutral | benign | benign | tolerated |
| ENST00000373409.8 | rs558234193 | V245L | tolerated - low confidence | benign | neutral | neutral | neutral | neutral | benign | benign | tolerated |
| ENST00000373409.8 | rs1189539975 | V246M | tolerated - low confidence | possibly damaging | low | neutral | neutral | neutral | benign | benign | tolerated |
| ENST00000373409.8 | rs1392455763 | V246A | tolerated - low confidence | possibly damaging | low | neutral | neutral | neutral | benign | benign | tolerated |
| ENST00000373409.8 | rs2076780639 | D247N | deleterious - low confidence | probably damaging | medium | Disease | neutral | neutral | benign | damaging | tolerated |
| ENST00000373409.8 | rs1443276179 | D247A | tolerated - low confidence | possibly damaging | medium | Disease | neutral | neutral | benign | damaging | tolerated |
| ENST00000373409.8 | rs1164668665 | L248I | tolerated - low confidence | benign | low | neutral | neutral | neutral | benign | benign | tolerated |
| ENST00000373409.8 | rs1164668665 | L248F | deleterious - low confidence | possibly damaging | medium | Disease | neutral | neutral | benign | benign | tolerated |
| ENST00000373409.8 | rs1369586319 | V249L | tolerated - low confidence | benign | neutral | neutral | neutral | neutral | benign | benign | tolerated |
| ENST00000373409.8 | rs1457962219 | V249A | deleterious - low confidence | benign | low | neutral | neutral | neutral | benign | benign | tolerated |
| ENST00000373409.8 | rs1164551922 | S250R | tolerated - low confidence | benign | medium | neutral | neutral | Disease | benign | damaging | tolerated |
| ENST00000373409.8 | rs749406769 | Y251H | tolerated - low confidence | benign | neutral | neutral | neutral | neutral | benign | benign | tolerated |
| ENST00000373409.8 | rs2076782519 | Y251F | tolerated - low confidence | benign | low | neutral | neutral | neutral | benign | benign | tolerated |
| ENST00000373409.8 | rs2076782937 | A252T | tolerated - low confidence | probably damaging | medium | neutral | neutral | neutral | benign | benign | tolerated |
| ENST00000373409.8 | rs369777528 | V254M | deleterious - low confidence | possibly damaging | low | neutral | neutral | neutral | benign | benign | tolerated |
| ENST00000373409.8 | rs369777528 | V254L | tolerated - low confidence | benign | low | neutral | neutral | neutral | benign | benign | tolerated |
| ENST00000373409.8 | rs147273707 | F257L | tolerated - low confidence | benign | neutral | neutral | neutral | neutral | benign | benign | tolerated |
| ENST00000373409.8 | rs760663829 | R258Q | deleterious - low confidence | probably damaging | medium | Disease | neutral | Disease | Pathogenic | damaging | tolerated |
| ENST00000373409.8 | rs200363835 | G259R | tolerated - low confidence | benign | medium | Disease | neutral | Disease | benign | benign | tolerated |
| ENST00000373409.8 | rs759062689 | D260N | deleterious - low confidence | probably damaging | medium | Disease | neutral | Disease | Pathogenic | damaging | tolerated |
| ENST00000373409.8 | rs1575528520 | D260G | deleterious - low confidence | probably damaging | high | Disease | Disease | Disease | Pathogenic | damaging | tolerated |
| ENST00000373409.8 | rs1250192329 | D260E | deleterious - low confidence | probably damaging | medium | Disease | Disease | Disease | Pathogenic | damaging | tolerated |
| ENST00000373409.8 | rs764921647 | F261S | deleterious - low confidence | probably damaging | high | Disease | neutral | Disease | Pathogenic | damaging | tolerated |
| ENST00000373409.8 | rs138347224 | F261L | deleterious - low confidence | probably damaging | high | Disease | neutral | Disease | Pathogenic | damaging | tolerated |
| ENST00000373409.8 | rs74521813 | V262L | deleterious - low confidence | possibly damaging | medium | neutral | neutral | neutral | benign | damaging | tolerated |
| ENST00000373409.8 | rs758434623 | M263I | deleterious - low confidence | benign | low | Disease | neutral | neutral | benign | benign | tolerated |
| ENST00000373409.8 | rs2076787897 | P266A | deleterious - low confidence | probably damaging | medium | Disease | neutral | neutral | Pathogenic | damaging | tolerated |
| ENST00000373409.8 | rs756696112 | P266L | deleterious - low confidence | probably damaging | high | Disease | Disease | Disease | Pathogenic | damaging | tolerated |
| ENST00000373409.8 | rs574418679 | P268L | deleterious - low confidence | probably damaging | high | Disease | Disease | Disease | Pathogenic | damaging | tolerated |
| ENST00000373409.8 | rs1302062405 | P271A | deleterious - low confidence | probably damaging | high | Disease | Disease | Disease | Pathogenic | damaging | tolerated |
| ENST00000373409.8 | rs779161392 | N272S | deleterious - low confidence | probably damaging | high | Disease | neutral | Disease | benign | damaging | tolerated |
| ENST00000373409.8 | rs779161392 | N272I | deleterious - low confidence | probably damaging | high | Disease | Disease | Disease | Pathogenic | damaging | tolerated |
| ENST00000373409.8 | rs1397748790 | N272K | deleterious - low confidence | probably damaging | high | Disease | Disease | Disease | Pathogenic | damaging | tolerated |
| ENST00000373409.8 | rs748381682 | M273T | deleterious - low confidence | probably damaging | medium | Disease | neutral | Disease | benign | damaging | tolerated |
| ENST00000373409.8 | rs771839689 | M273I | tolerated - low confidence | probably damaging | low | Disease | neutral | neutral | benign | damaging | tolerated |
| ENST00000373409.8 | rs560127147 | V274F | deleterious - low confidence | benign | high | Disease | Disease | Disease | Pathogenic | damaging | tolerated |
| ENST00000373409.8 | rs1270242967 | F275Y | tolerated - low confidence | probably damaging | low | neutral | neutral | neutral | Pathogenic | damaging | tolerated |
| ENST00000373409.8 | rs147342917 | I276L | deleterious - low confidence | possibly damaging | medium | Disease | neutral | neutral | benign | damaging | tolerated |
| ENST00000373409.8 | rs147342917 | I276V | tolerated - low confidence | possibly damaging | neutral | neutral | neutral | neutral | benign | damaging | tolerated |
| ENST00000373409.8 | rs201461954 | I276T | deleterious - low confidence | probably damaging | medium | Disease | Disease | Disease | benign | damaging | tolerated |
| ENST00000373409.8 | rs1575528864 | I276M | deleterious - low confidence | probably damaging | medium | Disease | neutral | Disease | benign | damaging | tolerated |
| ENST00000373409.8 | rs759057764 | G278D | deleterious - low confidence | probably damaging | high | Disease | Disease | Disease | Pathogenic | damaging | tolerated |
| ENST00000373409.8 | rs759057764 | G278A | deleterious - low confidence | probably damaging | high | Disease | Disease | Disease | Pathogenic | damaging | tolerated |
| ENST00000373409.8 | rs769254543 | C281S | deleterious - low confidence | probably damaging | high | Disease | neutral | Disease | Pathogenic | damaging | tolerated |
| ENST00000373409.8 | rs775195752 | C281W | deleterious - low confidence | probably damaging | high | Disease | Disease | Disease | Pathogenic | damaging | tolerated |
| ENST00000373409.8 | rs767930830 | A282T | tolerated - low confidence | possibly damaging | medium | neutral | neutral | Disease | benign | damaging | tolerated |
| ENST00000373409.8 | rs767930830 | A282P | tolerated - low confidence | probably damaging | medium | Disease | neutral | Disease | benign | damaging | tolerated |
| ENST00000373409.8 | rs761217423 | A282D | tolerated - low confidence | probably damaging | medium | Disease | neutral | neutral | benign | damaging | tolerated |
| ENST00000373409.8 | rs754340171 | N283S | tolerated - low confidence | benign | neutral | neutral | neutral | neutral | benign | benign | tolerated |
| ENST00000373409.8 | rs375077876 | N283K | tolerated - low confidence | benign | neutral | neutral | neutral | neutral | benign | benign | tolerated |
| ENST00000373409.8 | rs758623915 | G284R | tolerated - low confidence | benign | neutral | Disease | neutral | neutral | benign | benign | tolerated |
| ENST00000373409.8 | rs781186031 | L287V | deleterious - low confidence | probably damaging | medium | Disease | neutral | neutral | benign | damaging | tolerated |
| ENST00000373409.8 | rs2076795017 | L287Q | deleterious - low confidence | probably damaging | high | Disease | Disease | Disease | Pathogenic | damaging | tolerated |
| ENST00000373409.8 | rs2076795429 | S288P | tolerated - low confidence | probably damaging | neutral | neutral | neutral | neutral | benign | damaging | tolerated |
| ENST00000373409.8 | rs769294499 | Q289H | deleterious - low confidence | possibly damaging | medium | neutral | neutral | Disease | benign | damaging | tolerated |
| ENST00000373409.8 | rs1699312336 | E290K | deleterious - low confidence | benign | medium | Disease | Disease | Disease | benign | damaging | tolerated |
| ENST00000373409.8 | rs1316565909 | E290G | deleterious - low confidence | probably damaging | medium | Disease | Disease | Disease | benign | damaging | tolerated |
| ENST00000373409.8 | rs1699312862 | E292K | deleterious - low confidence | possibly damaging | high | Disease | Disease | Disease | Pathogenic | damaging | tolerated |
| ENST00000373409.8 | rs758873309 | A293D | deleterious - low confidence | probably damaging | low | neutral | neutral | Disease | benign | damaging | tolerated |
| ENST00000373409.8 | rs758873309 | A293G | tolerated - low confidence | probably damaging | medium | neutral | neutral | Disease | benign | damaging | tolerated |
| ENST00000373409.8 | rs758873309 | A293V | deleterious - low confidence | possibly damaging | medium | neutral | neutral | Disease | Pathogenic | damaging | tolerated |
| ENST00000373409.8 | rs72551347 | I295T | deleterious - low confidence | benign | low | Disease | Disease | Disease | Pathogenic | benign | tolerated |
| ENST00000373409.8 | rs928021157 | S298Y | deleterious - low confidence | probably damaging | high | Disease | Disease | Disease | benign | damaging | tolerated |
| ENST00000373409.8 | rs1699315314 | G299E | deleterious - low confidence | probably damaging | medium | Disease | Disease | Disease | benign | damaging | tolerated |
| ENST00000373409.8 | rs1055696021 | H301R | deleterious - low confidence | probably damaging | high | Disease | neutral | Disease | benign | damaging | tolerated |
| ENST00000373409.8 | rs1451220464 | G302E | deleterious - low confidence | probably damaging | high | Disease | Disease | Disease | Pathogenic | damaging | tolerated |
| ENST00000373409.8 | rs1199920513 | I303V | tolerated - low confidence | benign | neutral | neutral | neutral | neutral | benign | damaging | tolerated |
| ENST00000373409.8 | rs747099261 | I303T | deleterious - low confidence | benign | medium | neutral | neutral | Disease | benign | damaging | tolerated |
| ENST00000373409.8 | rs770930440 | V304M | deleterious - low confidence | probably damaging | high | Disease | Disease | Disease | Pathogenic | damaging | tolerated |
| ENST00000373409.8 | rs1699316631 | V305L | deleterious - low confidence | benign | medium | neutral | neutral | Disease | benign | damaging | tolerated |
| ENST00000373409.8 | rs781412186 | V305A | deleterious - low confidence | probably damaging | medium | neutral | Disease | Disease | Pathogenic | damaging | tolerated |
| ENST00000373409.8 | rs1169717734 | S307Y | deleterious - low confidence | probably damaging | high | Disease | Disease | Disease | Pathogenic | damaging | tolerated |
| ENST00000373409.8 | rs1169717734 | S307F | deleterious - low confidence | probably damaging | high | Disease | Disease | Disease | benign | damaging | tolerated |
| ENST00000373409.8 | rs746111352 | G309R | deleterious - low confidence | probably damaging | high | Disease | Disease | Disease | Pathogenic | damaging | damaging |
| ENST00000373409.8 | rs62625011 | G309E | deleterious - low confidence | probably damaging | high | Disease | Disease | Disease | Pathogenic | damaging | damaging |
| ENST00000373409.8 | rs1301476253 | S310L | deleterious - low confidence | probably damaging | high | Disease | neutral | Disease | Pathogenic | damaging | tolerated |
| ENST00000373409.8 | rs1699317728 | M311V | deleterious - low confidence | possibly damaging | medium | neutral | Disease | Disease | benign | damaging | tolerated |
| ENST00000373409.8 | rs1699317899 | M311R | deleterious - low confidence | possibly damaging | high | Disease | Disease | Disease | Pathogenic | damaging | tolerated |
| ENST00000373409.8 | rs2126030402 | V312I | deleterious - low confidence | probably damaging | low | neutral | neutral | Disease | benign | damaging | tolerated |
| ENST00000373409.8 | rs1699318263 | S313L | deleterious - low confidence | benign | high | Disease | neutral | neutral | benign | benign | tolerated |
| ENST00000373409.8 | rs1575824807 | I315L | deleterious - low confidence | probably damaging | low | neutral | neutral | neutral | benign | damaging | tolerated |
| ENST00000373409.8 | rs1575824807 | I315V | deleterious - low confidence | probably damaging | medium | neutral | neutral | Disease | benign | damaging | tolerated |
| ENST00000373409.8 | rs769666128 | P316T | deleterious - low confidence | probably damaging | low | neutral | neutral | Disease | benign | damaging | tolerated |
| ENST00000373409.8 | rs769666128 | P316S | deleterious - low confidence | probably damaging | medium | Disease | neutral | Disease | benign | damaging | tolerated |
| ENST00000373409.8 | rs114000345 | K318E | deleterious - low confidence | benign | neutral | neutral | neutral | Disease | benign | damaging | tolerated |
| ENST00000373409.8 | rs562389152 | A320P | deleterious - low confidence | probably damaging | high | Disease | neutral | Disease | Pathogenic | damaging | tolerated |
| ENST00000373409.8 | rs775405878 | A320V | deleterious - low confidence | probably damaging | medium | neutral | neutral | Disease | benign | damaging | tolerated |
| ENST00000373409.8 | rs749159613 | M321V | deleterious - low confidence | probably damaging | medium | neutral | neutral | Disease | benign | damaging | tolerated |
| ENST00000373409.8 | rs2126030462 | M321T | tolerated - low confidence | probably damaging | medium | neutral | neutral | Disease | benign | damaging | tolerated |
| ENST00000373409.8 | rs200903749 | I323V | deleterious - low confidence | probably damaging | medium | neutral | neutral | Disease | benign | damaging | tolerated |
| ENST00000373409.8 | rs17851756 | I323T | deleterious - low confidence | probably damaging | high | Disease | neutral | Disease | Pathogenic | damaging | tolerated |
| ENST00000373409.8 | rs774438667 | A324S | deleterious - low confidence | probably damaging | medium | Disease | neutral | Disease | benign | damaging | tolerated |
| ENST00000373409.8 | rs761316504 | A324V | deleterious - low confidence | probably damaging | medium | Disease | neutral | Disease | benign | damaging | tolerated |
| ENST00000373409.8 | rs1269029361 | A326T | deleterious - low confidence | probably damaging | medium | Disease | neutral | Disease | Pathogenic | damaging | tolerated |
| ENST00000373409.8 | rs372326047 | L327S | deleterious - low confidence | probably damaging | high | Disease | neutral | Disease | Pathogenic | damaging | tolerated |
| ENST00000373409.8 | rs1339405023 | I330L | deleterious - low confidence | probably damaging | low | neutral | neutral | neutral | benign | damaging | tolerated |
| ENST00000373409.8 | rs202035422 | I330T | deleterious - low confidence | probably damaging | high | Disease | Disease | Disease | benign | damaging | tolerated |
| ENST00000373409.8 | rs72551348 | Q332R | deleterious - low confidence | probably damaging | high | Disease | Disease | Disease | Pathogenic | damaging | tolerated |
| ENST00000373409.8 | rs1699322110 | T333A | deleterious - low confidence | probably damaging | medium | neutral | neutral | Disease | benign | damaging | tolerated |
| ENST00000373409.8 | rs1699322267 | T333R | deleterious - low confidence | probably damaging | low | neutral | neutral | Disease | benign | damaging | tolerated |
| ENST00000373409.8 | rs757687307 | L335Q | deleterious - low confidence | probably damaging | high | Disease | Disease | Disease | Pathogenic | damaging | tolerated |
| ENST00000373409.8 | rs1181740769 | W336S | deleterious - low confidence | probably damaging | high | Disease | Disease | Disease | Pathogenic | damaging | tolerated |
| ENST00000373409.8 | rs139607673 | R337W | deleterious - low confidence | probably damaging | high | Disease | Disease | Disease | Pathogenic | damaging | tolerated |
| ENST00000373409.8 | rs750453538 | R337Q | deleterious - low confidence | probably damaging | high | Disease | Disease | Disease | Pathogenic | damaging | tolerated |
| ENST00000373409.8 | rs750453538 | R337P | deleterious - low confidence | probably damaging | high | Disease | Disease | Disease | Pathogenic | damaging | tolerated |
| ENST00000373409.8 | rs1445321655 | Y338C | deleterious - low confidence | probably damaging | medium | Disease | neutral | Disease | Pathogenic | damaging | tolerated |
| ENST00000373409.8 | rs748591879 | T339A | deleterious - low confidence | probably damaging | medium | neutral | neutral | Disease | benign | damaging | tolerated |
| ENST00000373409.8 | rs72551349 | R342G | tolerated - low confidence | benign | medium | neutral | neutral | Disease | benign | benign | tolerated |
| ENST00000373409.8 | rs780354743 | R342Q | tolerated - low confidence | benign | medium | neutral | neutral | Disease | benign | benign | tolerated |
| ENST00000373409.8 | rs773195449 | P343T | deleterious - low confidence | probably damaging | medium | Disease | neutral | Disease | Pathogenic | damaging | tolerated |
| ENST00000373409.8 | rs773195449 | P343S | deleterious - low confidence | probably damaging | high | Disease | neutral | Disease | Pathogenic | damaging | tolerated |
| ENST00000373409.8 | rs144978321 | S344W | deleterious - low confidence | probably damaging | medium | Disease | neutral | Disease | benign | benign | tolerated |
| ENST00000373409.8 | rs144978321 | S344L | deleterious - low confidence | possibly damaging | medium | neutral | neutral | neutral | benign | benign | tolerated |
| ENST00000373409.8 | rs1336725166 | N345S | deleterious - low confidence | probably damaging | medium | neutral | neutral | Disease | benign | damaging | tolerated |
| ENST00000373409.8 | rs149750520 | N345K | deleterious - low confidence | probably damaging | medium | neutral | neutral | Disease | benign | damaging | tolerated |
| ENST00000373409.8 | rs1699512749 | L346P | deleterious - low confidence | probably damaging | high | Disease | neutral | Disease | Pathogenic | damaging | tolerated |
| ENST00000373409.8 | rs771899094 | A347T | deleterious - low confidence | possibly damaging | medium | neutral | neutral | Disease | benign | damaging | tolerated |
| ENST00000373409.8 | rs771899094 | A347S | tolerated - low confidence | benign | low | neutral | neutral | Disease | benign | damaging | tolerated |
| ENST00000373409.8 | rs201372184 | A347V | deleterious - low confidence | possibly damaging | medium | neutral | neutral | Disease | benign | damaging | tolerated |
| ENST00000373409.8 | rs1575830712 | N348K | tolerated - low confidence | benign | neutral | neutral | neutral | Disease | benign | benign | tolerated |
| ENST00000373409.8 | rs776227074 | T350K | deleterious - low confidence | probably damaging | high | Disease | neutral | Disease | Pathogenic | damaging | tolerated |
| ENST00000373409.8 | rs776227074 | T350M | deleterious - low confidence | probably damaging | high | neutral | neutral | Disease | Pathogenic | damaging | tolerated |
| ENST00000373409.8 | rs1699514998 | I351M | tolerated - low confidence | possibly damaging | low | neutral | neutral | neutral | benign | benign | tolerated |
| ENST00000373409.8 | rs549391527 | L352V | deleterious - low confidence | probably damaging | medium | neutral | neutral | Disease | benign | damaging | tolerated |
| ENST00000373409.8 | rs549391527 | L352F | deleterious - low confidence | probably damaging | medium | Disease | neutral | Disease | benign | damaging | tolerated |
| ENST00000373409.8 | rs1699515433 | V353A | deleterious - low confidence | probably damaging | medium | neutral | neutral | Disease | benign | damaging | tolerated |
| ENST00000373409.8 | rs1559414817 | W355R | deleterious - low confidence | probably damaging | high | Disease | Disease | Disease | Pathogenic | damaging | tolerated |
| ENST00000373409.8 | rs1699515943 | L356Q | deleterious - low confidence | probably damaging | high | Disease | Disease | Disease | Pathogenic | damaging | tolerated |
| ENST00000373409.8 | rs767850186 | P357T | deleterious - low confidence | probably damaging | high | Disease | Disease | Disease | Pathogenic | damaging | damaging |
| ENST00000373409.8 | rs72551351 | Q358P | deleterious - low confidence | probably damaging | high | Disease | Disease | Disease | benign | damaging | tolerated |
| ENST00000373409.8 | rs72551351 | Q358R | deleterious - low confidence | probably damaging | high | Disease | Disease | Disease | Pathogenic | damaging | tolerated |
| ENST00000373409.8 | rs886044684 | N359T | deleterious - low confidence | probably damaging | medium | Disease | Disease | Disease | Pathogenic | damaging | tolerated |
| ENST00000373409.8 | rs886044684 | N359S | deleterious - low confidence | probably damaging | medium | neutral | neutral | Disease | Pathogenic | damaging | tolerated |
| ENST00000373409.8 | rs1699517542 | N359K | deleterious - low confidence | probably damaging | medium | Disease | Disease | Disease | Pathogenic | damaging | tolerated |
| ENST00000373409.8 | rs267599273 | D360N | deleterious - low confidence | possibly damaging | high | Disease | Disease | Disease | Pathogenic | damaging | tolerated |
| ENST00000373409.8 | rs755218546 | G363S | deleterious - low confidence | probably damaging | high | Disease | Disease | Disease | Pathogenic | benign | tolerated |
| ENST00000373409.8 | rs755218546 | G363C | deleterious - low confidence | probably damaging | medium | Disease | neutral | Disease | Pathogenic | benign | tolerated |
| ENST00000373409.8 | rs752968297 | G363D | deleterious - low confidence | probably damaging | medium | Disease | Disease | Disease | Pathogenic | benign | tolerated |
| ENST00000373409.8 | rs752968297 | G363V | deleterious - low confidence | probably damaging | medium | Disease | Disease | Disease | benign | benign | tolerated |
| ENST00000373409.8 | rs34946978 | P365R | deleterious - low confidence | probably damaging | high | Disease | Disease | Disease | benign | damaging | tolerated |
| ENST00000373409.8 | rs34946978 | P365L | deleterious - low confidence | probably damaging | high | Disease | Disease | Disease | Pathogenic | damaging | tolerated |
| ENST00000373409.8 | rs367784507 | M366V | deleterious - low confidence | benign | neutral | neutral | neutral | Disease | benign | benign | tolerated |
| ENST00000373409.8 | rs371224646 | M366T | deleterious - low confidence | benign | neutral | neutral | neutral | Disease | benign | benign | tolerated |
| ENST00000373409.8 | rs1699591522 | T367N | deleterious - low confidence | probably damaging | high | Disease | Disease | Disease | Pathogenic | damaging | tolerated |
| ENST00000373409.8 | rs55750087 | R368G | deleterious - low confidence | probably damaging | high | Disease | Disease | Disease | Pathogenic | damaging | tolerated |
| ENST00000373409.8 | rs55750087 | R368C | deleterious - low confidence | probably damaging | high | Disease | Disease | Disease | benign | damaging | tolerated |
| ENST00000373409.8 | rs374047963 | R368H | deleterious - low confidence | probably damaging | high | Disease | Disease | Disease | Pathogenic | damaging | tolerated |
| ENST00000373409.8 | rs374047963 | R368P | deleterious - low confidence | probably damaging | high | Disease | Disease | Disease | Pathogenic | damaging | tolerated |
| ENST00000373409.8 | rs374047963 | R368L | deleterious - low confidence | probably damaging | medium | Disease | Disease | Disease | benign | damaging | tolerated |
| ENST00000373409.8 | rs72551352 | A369T | deleterious - low confidence | probably damaging | high | Disease | neutral | Disease | Pathogenic | damaging | tolerated |
| ENST00000373409.8 | rs1218307967 | A369G | deleterious - low confidence | probably damaging | high | Disease | neutral | neutral | Pathogenic | damaging | tolerated |
| ENST00000373409.8 | rs748989741 | I371V | deleterious - low confidence | probably damaging | low | neutral | neutral | neutral | benign | damaging | tolerated |
| ENST00000373409.8 | rs1285354199 | T372I | deleterious - low confidence | probably damaging | high | Disease | Disease | Disease | Pathogenic | damaging | tolerated |
| ENST00000373409.8 | rs1699594140 | H373N | deleterious - low confidence | probably damaging | high | Disease | Disease | Disease | Pathogenic | damaging | tolerated |
| ENST00000373409.8 | rs1699594140 | H373Y | deleterious - low confidence | probably damaging | high | Disease | Disease | Disease | benign | damaging | tolerated |
| ENST00000373409.8 | rs1276913504 | G375S | deleterious - low confidence | probably damaging | high | Disease | Disease | Disease | Pathogenic | damaging | damaging |
| ENST00000373409.8 | rs72551353 | S376F | deleterious - low confidence | probably damaging | medium | Disease | Disease | Disease | Pathogenic | damaging | tolerated |
| ENST00000373409.8 | rs1349037761 | H377R | deleterious - low confidence | probably damaging | medium | Disease | Disease | Disease | Pathogenic | damaging | tolerated |
| ENST00000373409.8 | rs773679964 | G378S | deleterious - low confidence | probably damaging | medium | neutral | neutral | Disease | Pathogenic | damaging | tolerated |
| ENST00000373409.8 | rs1283652721 | G378V | deleterious - low confidence | probably damaging | high | Disease | Disease | Disease | Pathogenic | damaging | tolerated |
| ENST00000373409.8 | rs1699596168 | E381G | deleterious - low confidence | probably damaging | high | Disease | Disease | Disease | Pathogenic | damaging | tolerated |
| ENST00000373409.8 | rs771550944 | S382C | deleterious - low confidence | possibly damaging | low | Disease | neutral | Disease | Pathogenic | benign | tolerated |
| ENST00000373409.8 | rs72551354 | S382R | deleterious - low confidence | benign | low | Disease | Disease | Disease | Pathogenic | benign | tolerated |
| ENST00000373409.8 | rs777289979 | N385D | deleterious - low confidence | probably damaging | medium | Disease | Disease | Disease | Pathogenic | damaging | tolerated |
| ENST00000373409.8 | rs759467827 | N385S | deleterious - low confidence | probably damaging | medium | neutral | neutral | Disease | benign | damaging | tolerated |
| ENST00000373409.8 | rs1699597581 | G386S | deleterious - low confidence | possibly damaging | high | Disease | Disease | Disease | Pathogenic | damaging | damaging |
| ENST00000373409.8 | rs143573365 | V387I | deleterious - low confidence | probably damaging | low | neutral | neutral | Disease | benign | damaging | tolerated |
| ENST00000373409.8 | rs901936528 | P388S | deleterious - low confidence | probably damaging | high | Disease | Disease | Disease | Pathogenic | damaging | damaging |
| ENST00000373409.8 | rs1559415403 | P388R | deleterious - low confidence | probably damaging | high | Disease | Disease | Disease | Pathogenic | damaging | damaging |
| ENST00000373409.8 | rs1699598751 | M389V | deleterious - low confidence | probably damaging | low | Disease | neutral | Disease | Pathogenic | damaging | tolerated |
| ENST00000373409.8 | rs527483899 | M389T | deleterious - low confidence | probably damaging | high | Disease | Disease | Disease | Pathogenic | damaging | tolerated |
| ENST00000373409.8 | rs1162609742 | M389I | deleterious - low confidence | probably damaging | medium | Disease | neutral | Disease | Pathogenic | damaging | tolerated |
| ENST00000373409.8 | rs1365887380 | V390L | deleterious - low confidence | probably damaging | low | neutral | neutral | Disease | Pathogenic | damaging | tolerated |
| ENST00000373409.8 | rs1559415443 | M391I | deleterious - low confidence | probably damaging | medium | Disease | Disease | Disease | benign | benign | tolerated |
| ENST00000373409.8 | rs1183097052 | M392V | deleterious - low confidence | benign | low | Disease | neutral | Disease | benign | damaging | tolerated |
| ENST00000373409.8 | rs751355128 | M392I | deleterious - low confidence | benign | low | neutral | neutral | Disease | benign | damaging | tolerated |
| ENST00000373409.8 | rs1286993592 | P393S | deleterious - low confidence | probably damaging | high | Disease | Disease | Disease | benign | damaging | damaging |
| ENST00000373409.8 | rs886043066 | P393L | deleterious - low confidence | probably damaging | high | Disease | Disease | Disease | Pathogenic | damaging | damaging |
| ENST00000373409.8 | rs1430980091 | L394F | deleterious - low confidence | probably damaging | medium | neutral | neutral | Disease | benign | damaging | tolerated |
| ENST00000373409.8 | rs1699600557 | F395L | deleterious - low confidence | probably damaging | medium | Disease | neutral | Disease | benign | damaging | tolerated |
| ENST00000373409.8 | rs367897068 | G396D | deleterious - low confidence | probably damaging | high | Disease | Disease | Disease | Pathogenic | damaging | tolerated |
| ENST00000373409.8 | rs367897068 | G396A | deleterious - low confidence | probably damaging | low | neutral | neutral | Disease | benign | damaging | tolerated |
| ENST00000373409.8 | rs367897068 | G396V | deleterious - low confidence | probably damaging | medium | Disease | Disease | Disease | Pathogenic | damaging | tolerated |
| ENST00000373409.8 | rs755527328 | M399T | deleterious - low confidence | probably damaging | medium | Disease | neutral | Disease | benign | damaging | tolerated |
| ENST00000373409.8 | rs779591634 | D400G | deleterious - low confidence | probably damaging | medium | Disease | neutral | Disease | Pathogenic | damaging | tolerated |
| ENST00000373409.8 | rs28934877 | N401H | deleterious - low confidence | probably damaging | high | Disease | Disease | Disease | Pathogenic | damaging | tolerated |
| ENST00000373409.8 | rs28934877 | N401D | deleterious - low confidence | probably damaging | high | Disease | Disease | Disease | benign | damaging | tolerated |
| ENST00000373409.8 | rs754652167 | N401K | deleterious - low confidence | probably damaging | medium | Disease | Disease | Disease | Pathogenic | damaging | tolerated |
| ENST00000373409.8 | rs72551355 | A402P | deleterious - low confidence | probably damaging | high | Disease | Disease | Disease | Pathogenic | damaging | tolerated |
| ENST00000373409.8 | rs778766461 | R404S | deleterious - low confidence | probably damaging | medium | Disease | Disease | Disease | Pathogenic | damaging | tolerated |
| ENST00000373409.8 | rs778766461 | R404C | deleterious - low confidence | probably damaging | high | Disease | Disease | Disease | Pathogenic | damaging | tolerated |
| ENST00000373409.8 | rs140613392 | R404H | deleterious - low confidence | probably damaging | low | neutral | neutral | Disease | Pathogenic | damaging | tolerated |
| ENST00000373409.8 | rs140613392 | R404L | deleterious - low confidence | probably damaging | medium | Disease | Disease | Disease | Pathogenic | damaging | tolerated |
| ENST00000373409.8 | rs777238544 | M405L | deleterious - low confidence | benign | low | neutral | neutral | Disease | benign | benign | tolerated |
| ENST00000373409.8 | rs777238544 | M405V | tolerated - low confidence | possibly damaging | low | neutral | neutral | Disease | benign | benign | tolerated |
| ENST00000373409.8 | rs549328655 | M405T | deleterious - low confidence | possibly damaging | high | Disease | Disease | Disease | benign | benign | tolerated |
| ENST00000373409.8 | rs2126038022 | M405I | tolerated - low confidence | benign | low | neutral | neutral | Disease | benign | benign | tolerated |
| ENST00000373409.8 | rs1699604029 | E406V | deleterious - low confidence | probably damaging | low | neutral | neutral | Disease | benign | damaging | tolerated |
| ENST00000373409.8 | rs1559415650 | K408R | tolerated - low confidence | benign | neutral | neutral | neutral | neutral | benign | benign | tolerated |
| ENST00000373409.8 | rs770254031 | G409E | deleterious - low confidence | probably damaging | high | Disease | Disease | Disease | benign | damaging | tolerated |
| ENST00000373409.8 | rs1272833298 | A410V | deleterious - low confidence | probably damaging | medium | neutral | neutral | Disease | benign | damaging | tolerated |
| ENST00000373409.8 | rs1444362528 | G411E | deleterious - low confidence | probably damaging | medium | Disease | Disease | Disease | Pathogenic | damaging | tolerated |
| ENST00000373409.8 | rs36076514 | V412M | deleterious - low confidence | possibly damaging | high | Disease | neutral | Disease | benign | damaging | tolerated |
| ENST00000373409.8 | rs36076514 | V412L | tolerated - low confidence | benign | low | neutral | neutral | Disease | benign | damaging | tolerated |
| ENST00000373409.8 | rs1156645272 | T413S | tolerated - low confidence | benign | neutral | neutral | neutral | neutral | benign | benign | tolerated |
| ENST00000373409.8 | rs1699606066 | T413I | tolerated - low confidence | possibly damaging | low | neutral | neutral | Disease | benign | benign | tolerated |
| ENST00000373409.8 | rs763012065 | N415K | deleterious - low confidence | probably damaging | medium | neutral | neutral | neutral | benign | damaging | tolerated |
| ENST00000373409.8 | rs774573761 | T420A | deleterious - low confidence | possibly damaging | medium | neutral | Disease | neutral | benign | damaging | tolerated |
| ENST00000373409.8 | rs866185120 | S421F | deleterious - low confidence | probably damaging | medium | Disease | neutral | Disease | benign | damaging | tolerated |
| ENST00000373409.8 | rs750232648 | A427S | deleterious - low confidence | possibly damaging | medium | Disease | neutral | neutral | benign | damaging | tolerated |
| ENST00000373409.8 | rs756044146 | A427V | deleterious - low confidence | possibly damaging | medium | Disease | neutral | Disease | Pathogenic | damaging | tolerated |
| ENST00000373409.8 | rs72551356 | K429E | deleterious - low confidence | possibly damaging | medium | neutral | neutral | neutral | benign | damaging | tolerated |
| ENST00000373409.8 | rs1699608953 | K429R | deleterious - low confidence | possibly damaging | medium | neutral | neutral | neutral | benign | damaging | tolerated |
| ENST00000373409.8 | rs1699609546 | I432V | deleterious - low confidence | probably damaging | medium | neutral | neutral | neutral | benign | damaging | tolerated |
| ENST00000373409.8 | rs1413528689 | N433D | deleterious - low confidence | possibly damaging | medium | Disease | neutral | neutral | benign | damaging | tolerated |
| ENST00000373409.8 | rs754549295 | D434E | tolerated - low confidence | benign | low | Disease | neutral | neutral | benign | damaging | tolerated |
| ENST00000373409.8 | rs1699610425 | K435E | deleterious - low confidence | probably damaging | medium | Disease | neutral | neutral | benign | damaging | tolerated |
| ENST00000373409.8 | rs1699610736 | K435N | deleterious - low confidence | probably damaging | medium | Disease | neutral | neutral | benign | damaging | tolerated |
| ENST00000373409.8 | rs1306719122 | S436N | deleterious - low confidence | possibly damaging | medium | Disease | neutral | Disease | benign | damaging | tolerated |
| ENST00000373409.8 | rs1306719122 | S436T | deleterious - low confidence | benign | medium | neutral | neutral | neutral | benign | damaging | tolerated |
| ENST00000373409.8 | rs1306719122 | S436I | deleterious - low confidence | probably damaging | medium | Disease | neutral | Disease | benign | damaging | tolerated |
| ENST00000373409.8 | rs766626435 | Y437C | deleterious - low confidence | probably damaging | high | Disease | neutral | Disease | Pathogenic | damaging | tolerated |
| ENST00000373409.8 | rs766626435 | Y437F | deleterious - low confidence | probably damaging | medium | Disease | neutral | neutral | Pathogenic | damaging | tolerated |
| ENST00000373409.8 | rs1700497336 | K438N | deleterious - low confidence | probably damaging | high | Disease | neutral | Disease | Pathogenic | damaging | tolerated |
| ENST00000373409.8 | rs868814380 | E439K | deleterious - low confidence | possibly damaging | medium | Disease | neutral | neutral | benign | damaging | tolerated |
| ENST00000373409.8 | rs868814380 | E439Q | deleterious - low confidence | benign | medium | neutral | neutral | neutral | benign | damaging | tolerated |
| ENST00000373409.8 | rs1365374902 | E439G | deleterious - low confidence | probably damaging | medium | Disease | neutral | neutral | benign | damaging | tolerated |
| ENST00000373409.8 | rs1700498214 | I441V | deleterious - low confidence | benign | low | neutral | neutral | neutral | benign | damaging | tolerated |
| ENST00000373409.8 | rs1575869883 | I441N | deleterious - low confidence | possibly damaging | medium | Disease | neutral | Disease | Pathogenic | damaging | tolerated |
| ENST00000373409.8 | rs1289153891 | M442V | deleterious - low confidence | benign | medium | neutral | neutral | neutral | benign | damaging | tolerated |
| ENST00000373409.8 | rs202172337 | M442K | tolerated - low confidence | benign | low | neutral | neutral | neutral | benign | damaging | tolerated |
| ENST00000373409.8 | rs202172337 | M442T | deleterious - low confidence | benign | medium | neutral | neutral | neutral | benign | damaging | tolerated |
| ENST00000373409.8 | rs202172337 | M442R | deleterious - low confidence | benign | medium | neutral | neutral | neutral | benign | damaging | tolerated |
| ENST00000373409.8 | rs143033456 | R443C | deleterious - low confidence | possibly damaging | high | Disease | neutral | Disease | benign | damaging | tolerated |
| ENST00000373409.8 | rs748166510 | R443H | tolerated - low confidence | benign | medium | neutral | neutral | neutral | benign | damaging | tolerated |
| ENST00000373409.8 | rs748166510 | R443L | deleterious - low confidence | benign | medium | Disease | neutral | neutral | benign | damaging | tolerated |
| ENST00000373409.8 | rs1445186018 | L444F | deleterious - low confidence | probably damaging | medium | neutral | neutral | neutral | benign | damaging | tolerated |
| ENST00000373409.8 | rs758411577 | L444P | deleterious - low confidence | probably damaging | high | Disease | neutral | Disease | Pathogenic | damaging | tolerated |
| ENST00000373409.8 | rs1575870013 | S445P | deleterious - low confidence | probably damaging | high | Disease | neutral | Disease | Pathogenic | damaging | tolerated |
| ENST00000373409.8 | rs1248654212 | S446N | deleterious - low confidence | possibly damaging | medium | neutral | neutral | neutral | benign | benign | tolerated |
| ENST00000373409.8 | rs778015980 | S446R | tolerated - low confidence | benign | neutral | neutral | neutral | neutral | benign | benign | tolerated |
| ENST00000373409.8 | rs568508589 | L447F | deleterious - low confidence | probably damaging | high | neutral | neutral | neutral | benign | damaging | tolerated |
| ENST00000373409.8 | rs1700500987 | K449R | tolerated - low confidence | probably damaging | low | neutral | neutral | neutral | benign | damaging | tolerated |
| ENST00000373409.8 | rs1458644938 | D450N | deleterious - low confidence | probably damaging | high | Disease | Disease | neutral | Pathogenic | damaging | tolerated |
| ENST00000373409.8 | rs1482422312 | D450G | deleterious - low confidence | probably damaging | high | Disease | Disease | Disease | Pathogenic | damaging | tolerated |
| ENST00000373409.8 | rs1165346261 | D450E | deleterious - low confidence | probably damaging | high | Disease | neutral | neutral | Pathogenic | damaging | tolerated |
| ENST00000373409.8 | rs201427749 | R451C | deleterious - low confidence | probably damaging | high | Disease | Disease | Disease | Pathogenic | damaging | tolerated |
| ENST00000373409.8 | rs200370335 | R451H | deleterious - low confidence | probably damaging | medium | Disease | neutral | neutral | benign | damaging | tolerated |
| ENST00000373409.8 | rs200370335 | R451L | deleterious - low confidence | probably damaging | high | Disease | Disease | Disease | Pathogenic | damaging | tolerated |
| ENST00000373409.8 | rs114982090 | P452L | deleterious - low confidence | probably damaging | medium | Disease | Disease | Disease | Pathogenic | damaging | tolerated |
| ENST00000373409.8 | rs587784536 | V453M | tolerated - low confidence | benign | neutral | neutral | neutral | neutral | benign | benign | tolerated |
| ENST00000373409.8 | rs587784536 | V453L | deleterious - low confidence | benign | low | neutral | neutral | neutral | benign | benign | tolerated |
| ENST00000373409.8 | rs1312404188 | E454Q | deleterious - low confidence | probably damaging | medium | neutral | neutral | neutral | benign | benign | tolerated |
| ENST00000373409.8 | rs762488947 | P455L | deleterious - low confidence | probably damaging | high | Disease | Disease | Disease | Pathogenic | damaging | tolerated |
| ENST00000373409.8 | rs1700504653 | L456R | deleterious - low confidence | probably damaging | medium | Disease | neutral | Disease | Pathogenic | damaging | tolerated |
| ENST00000373409.8 | rs919221213 | D457Y | deleterious - low confidence | probably damaging | high | Disease | Disease | Disease | Pathogenic | damaging | tolerated |
| ENST00000373409.8 | rs1700505189 | D457V | deleterious - low confidence | probably damaging | high | Disease | Disease | Disease | Pathogenic | damaging | tolerated |
| ENST00000373409.8 | rs773957041 | D457E | deleterious - low confidence | probably damaging | low | neutral | neutral | neutral | benign | damaging | tolerated |
| ENST00000373409.8 | rs767264478 | L458M | deleterious - low confidence | probably damaging | medium | neutral | neutral | neutral | benign | damaging | tolerated |
| ENST00000373409.8 | rs527798161 | L458Q | deleterious - low confidence | probably damaging | medium | Disease | neutral | neutral | benign | damaging | tolerated |
| ENST00000373409.8 | rs1207971053 | A459T | deleterious - low confidence | probably damaging | medium | Disease | neutral | neutral | Pathogenic | damaging | tolerated |
| ENST00000373409.8 | rs373052727 | A459V | deleterious - low confidence | probably damaging | medium | neutral | neutral | neutral | Pathogenic | damaging | tolerated |
| ENST00000373409.8 | rs1201825340 | V460M | deleterious - low confidence | probably damaging | medium | neutral | neutral | neutral | benign | damaging | tolerated |
| ENST00000373409.8 | rs1201825340 | V460L | deleterious - low confidence | probably damaging | medium | neutral | neutral | neutral | benign | damaging | tolerated |
| ENST00000373409.8 | rs115410088 | F461L | deleterious - low confidence | probably damaging | high | Disease | neutral | Disease | Pathogenic | benign | tolerated |
| ENST00000373409.8 | rs1476500325 | W462R | deleterious - low confidence | probably damaging | high | Disease | Disease | Disease | Pathogenic | damaging | tolerated |
| ENST00000373409.8 | rs1424603943 | W462S | deleterious - low confidence | probably damaging | high | Disease | Disease | Disease | Pathogenic | damaging | tolerated |
| ENST00000373409.8 | rs753234232 | V463M | deleterious - low confidence | probably damaging | medium | Disease | neutral | neutral | benign | damaging | tolerated |
| ENST00000373409.8 | rs758454924 | E464K | deleterious - low confidence | probably damaging | high | Disease | Disease | Disease | Pathogenic | damaging | tolerated |
| ENST00000373409.8 | rs72551358 | E464A | deleterious - low confidence | probably damaging | high | Disease | Disease | Disease | Pathogenic | damaging | tolerated |
| ENST00000373409.8 | rs72551358 | E464G | deleterious - low confidence | probably damaging | high | Disease | Disease | Disease | Pathogenic | damaging | tolerated |
| ENST00000373409.8 | rs72551358 | E464V | deleterious - low confidence | probably damaging | high | Disease | Disease | Disease | Pathogenic | damaging | tolerated |
| ENST00000373409.8 | rs115944950 | E464D | deleterious - low confidence | probably damaging | medium | Disease | neutral | neutral | Pathogenic | damaging | tolerated |
| ENST00000373409.8 | rs1700509286 | F465I | deleterious - low confidence | possibly damaging | high | Disease | neutral | Disease | Pathogenic | damaging | tolerated |
| ENST00000373409.8 | rs1700509872 | F465Y | tolerated - low confidence | benign | neutral | neutral | neutral | neutral | benign | damaging | tolerated |
| ENST00000373409.8 | rs781623948 | M467I | deleterious - low confidence | probably damaging | low | neutral | neutral | neutral | benign | damaging | tolerated |
| ENST00000373409.8 | rs745603293 | R468K | tolerated - low confidence | probably damaging | medium | Disease | neutral | neutral | benign | damaging | tolerated |
| ENST00000373409.8 | rs1559420158 | H469D | deleterious - low confidence | probably damaging | high | Disease | Disease | Disease | Pathogenic | damaging | tolerated |
| ENST00000373409.8 | rs1559420158 | H469Y | deleterious - low confidence | probably damaging | medium | Disease | neutral | Disease | Pathogenic | damaging | tolerated |
| ENST00000373409.8 | rs1400939614 | K470Q | deleterious - low confidence | probably damaging | medium | Disease | neutral | neutral | benign | damaging | tolerated |
| ENST00000373409.8 | rs2126066747 | G471S | deleterious - low confidence | probably damaging | high | Disease | Disease | Disease | Pathogenic | damaging | damaging |
| ENST00000373409.8 | rs1187321852 | G471D | deleterious - low confidence | probably damaging | high | Disease | Disease | Disease | Pathogenic | damaging | damaging |
| ENST00000373409.8 | rs775532505 | A472T | deleterious - low confidence | probably damaging | medium | Disease | Disease | Disease | Pathogenic | damaging | tolerated |
| ENST00000373409.8 | rs1333719360 | A472V | deleterious - low confidence | probably damaging | high | Disease | Disease | Disease | Pathogenic | damaging | tolerated |
| ENST00000373409.8 | rs377565834 | P473S | deleterious - low confidence | benign | low | neutral | neutral | neutral | benign | damaging | tolerated |
| ENST00000373409.8 | rs773725816 | H474Y | deleterious - low confidence | possibly damaging | medium | Disease | neutral | Disease | Pathogenic | damaging | tolerated |
| ENST00000373409.8 | rs72551359 | L475M | deleterious - low confidence | probably damaging | high | neutral | neutral | neutral | Pathogenic | damaging | tolerated |
| ENST00000373409.8 | rs1700514037 | L475R | deleterious - low confidence | probably damaging | high | Disease | Disease | Disease | Pathogenic | damaging | tolerated |
| ENST00000373409.8 | rs566674185 | R476S | deleterious - low confidence | probably damaging | high | Disease | Disease | Disease | Pathogenic | damaging | tolerated |
| ENST00000373409.8 | rs566674185 | R476C | deleterious - low confidence | probably damaging | high | Disease | Disease | Disease | Pathogenic | damaging | tolerated |
| ENST00000373409.8 | rs150687296 | R476H | deleterious - low confidence | probably damaging | medium | Disease | Disease | neutral | Pathogenic | damaging | tolerated |
| ENST00000373409.8 | rs765612353 | A478T | deleterious - low confidence | probably damaging | medium | neutral | neutral | neutral | Pathogenic | damaging | tolerated |
| ENST00000373409.8 | rs765612353 | A478S | deleterious - low confidence | probably damaging | medium | neutral | neutral | neutral | benign | damaging | tolerated |
| ENST00000373409.8 | rs1700515616 | A479D | deleterious - low confidence | probably damaging | high | Disease | Disease | Disease | Pathogenic | damaging | tolerated |
| ENST00000373409.8 | rs753109787 | H480Y | deleterious - low confidence | probably damaging | low | neutral | neutral | neutral | Pathogenic | damaging | tolerated |
| ENST00000373409.8 | rs369610863 | H480Q | deleterious - low confidence | probably damaging | medium | neutral | neutral | neutral | Pathogenic | damaging | tolerated |
| ENST00000373409.8 | rs751579554 | D481N | deleterious - low confidence | probably damaging | low | neutral | neutral | neutral | Pathogenic | damaging | tolerated |
| ENST00000373409.8 | rs1433976375 | L482F | deleterious - low confidence | probably damaging | high | Disease | Disease | Disease | Pathogenic | damaging | tolerated |
| ENST00000373409.8 | rs1700516890 | T483S | deleterious - low confidence | probably damaging | low | neutral | neutral | neutral | benign | benign | tolerated |
| ENST00000373409.8 | rs1279219087 | T483I | deleterious - low confidence | probably damaging | medium | neutral | Disease | Disease | benign | benign | tolerated |
| ENST00000373409.8 | rs1176419046 | W484L | deleterious - low confidence | probably damaging | medium | Disease | Disease | Disease | Pathogenic | damaging | tolerated |
| ENST00000373409.8 | rs770903084 | Y485H | deleterious - low confidence | probably damaging | medium | Disease | Disease | neutral | Pathogenic | benign | tolerated |
| ENST00000373409.8 | rs1700517999 | Y485S | deleterious - low confidence | probably damaging | high | Disease | Disease | Disease | Pathogenic | benign | tolerated |
| ENST00000373409.8 | rs34993780 | Y487N | deleterious - low confidence | probably damaging | high | Disease | Disease | Disease | Pathogenic | damaging | tolerated |
| ENST00000373409.8 | rs34993780 | Y487H | deleterious - low confidence | probably damaging | medium | Disease | Disease | Disease | Pathogenic | damaging | tolerated |
| ENST00000373409.8 | rs34993780 | Y487D | deleterious - low confidence | probably damaging | high | Disease | Disease | Disease | Pathogenic | damaging | tolerated |
| ENST00000373409.8 | rs371183955 | H488Y | deleterious - low confidence | probably damaging | medium | neutral | neutral | neutral | Pathogenic | damaging | tolerated |
| ENST00000373409.8 | rs1700519300 | H488R | deleterious - low confidence | probably damaging | high | Disease | Disease | Disease | Pathogenic | damaging | tolerated |
| ENST00000373409.8 | rs72551360 | S489C | deleterious - low confidence | probably damaging | medium | Disease | neutral | neutral | benign | damaging | tolerated |
| ENST00000373409.8 | rs72551360 | S489F | deleterious - low confidence | probably damaging | high | Disease | neutral | Disease | Pathogenic | damaging | tolerated |
| ENST00000373409.8 | rs1300515739 | D491A | deleterious - low confidence | probably damaging | high | Disease | Disease | Disease | Pathogenic | damaging | tolerated |
| ENST00000373409.8 | rs747543462 | V492M | deleterious - low confidence | probably damaging | high | Disease | Disease | Disease | benign | damaging | tolerated |
| ENST00000373409.8 | rs2126066962 | I493V | deleterious - low confidence | probably damaging | medium | neutral | neutral | neutral | benign | damaging | tolerated |
| ENST00000373409.8 | rs771600393 | I493T | deleterious - low confidence | probably damaging | medium | Disease | neutral | neutral | benign | damaging | tolerated |
| ENST00000373409.8 | rs1700521067 | G494D | deleterious - low confidence | probably damaging | high | Disease | Disease | Disease | Pathogenic | benign | tolerated |
| ENST00000373409.8 | rs760289864 | V499I | tolerated - low confidence | benign | neutral | neutral | neutral | neutral | benign | benign | tolerated |
| ENST00000373409.8 | rs199723856 | V500M | deleterious - low confidence | possibly damaging | medium | Disease | neutral | neutral | benign | damaging | tolerated |
| ENST00000373409.8 | rs199723856 | V500L | deleterious - low confidence | benign | low | neutral | neutral | neutral | benign | damaging | tolerated |
| ENST00000373409.8 | rs763440969 | L501P | deleterious - low confidence | probably damaging | high | Disease | Disease | Disease | Pathogenic | damaging | tolerated |
| ENST00000373409.8 | rs1268705566 | T502R | tolerated - low confidence | possibly damaging | medium | Disease | neutral | Disease | Pathogenic | damaging | tolerated |
| ENST00000373409.8 | rs764439291 | V503M | deleterious - low confidence | possibly damaging | medium | Disease | neutral | neutral | benign | damaging | tolerated |
| ENST00000373409.8 | rs752117935 | V503A | tolerated - low confidence | benign | low | neutral | neutral | neutral | benign | damaging | tolerated |
| ENST00000373409.8 | rs1575871358 | A504V | tolerated - low confidence | benign | neutral | neutral | neutral | neutral | benign | benign | tolerated |
| ENST00000373409.8 | rs761746377 | F505L | tolerated - low confidence | probably damaging | low | neutral | neutral | neutral | benign | damaging | tolerated |
| ENST00000373409.8 | rs767732319 | F505Y | deleterious - low confidence | probably damaging | medium | neutral | neutral | neutral | benign | damaging | tolerated |
| ENST00000373409.8 | rs1372118451 | I506V | tolerated - low confidence | benign | low | neutral | neutral | neutral | benign | damaging | tolerated |
| ENST00000373409.8 | rs1341191921 | I506T | deleterious - low confidence | benign | low | Disease | neutral | neutral | benign | damaging | tolerated |
| ENST00000373409.8 | rs1575871447 | T507P | tolerated - low confidence | benign | medium | Disease | neutral | Disease | benign | benign | tolerated |
| ENST00000373409.8 | rs1575871447 | T507A | tolerated - low confidence | benign | neutral | neutral | neutral | neutral | benign | benign | tolerated |
| ENST00000373409.8 | rs1575871453 | T507I | tolerated - low confidence | benign | neutral | neutral | neutral | neutral | benign | damaging | tolerated |
| ENST00000373409.8 | rs780493798 | C510G | deleterious - low confidence | benign | medium | Disease | Disease | Disease | benign | benign | tolerated |
| ENST00000373409.8 | rs1700526717 | C510Y | deleterious - low confidence | possibly damaging | medium | Disease | Disease | Disease | benign | benign | tolerated |
| ENST00000373409.8 | rs1042709 | A512P | tolerated - low confidence | probably damaging | medium | Disease | neutral | Disease | benign | benign | tolerated |
| ENST00000373409.8 | rs1700527426 | G514S | tolerated - low confidence | possibly damaging | low | neutral | neutral | neutral | benign | benign | tolerated |
| ENST00000373409.8 | rs1575871592 | Y515C | tolerated - low confidence | benign | neutral | neutral | neutral | neutral | benign | benign | tolerated |
| ENST00000373409.8 | rs867393133 | R516W | deleterious - low confidence | probably damaging | medium | Disease | neutral | Disease | benign | damaging | tolerated |
| ENST00000373409.8 | rs778667717 | R516Q | tolerated - low confidence | possibly damaging | low | Disease | neutral | Disease | benign | damaging | tolerated |
| ENST00000373409.8 | rs778667717 | R516L | deleterious - low confidence | possibly damaging | medium | Disease | Disease | Disease | benign | damaging | tolerated |
| ENST00000373409.8 | rs1559420791 | K517T | deleterious - low confidence | probably damaging | high | Disease | Disease | Disease | benign | damaging | tolerated |
| ENST00000373409.8 | rs772037816 | K517N | deleterious - low confidence | probably damaging | medium | Disease | Disease | neutral | benign | damaging | tolerated |
| ENST00000373409.8 | rs1700529136 | C518Y | deleterious - low confidence | possibly damaging | high | Disease | Disease | Disease | benign | damaging | tolerated |
| ENST00000373409.8 | rs2126067193 | L519F | tolerated - low confidence | benign | neutral | neutral | neutral | neutral | benign | benign | tolerated |
| ENST00000373409.8 | rs1553624227 | G520R | tolerated - low confidence | probably damaging | low | neutral | neutral | neutral | benign | damaging | tolerated |
| ENST00000373409.8 | rs867885761 | G520E | deleterious - low confidence | probably damaging | medium | Disease | neutral | Disease | benign | damaging | tolerated |
| ENST00000373409.8 | rs867885761 | G520A | deleterious - low confidence | probably damaging | medium | neutral | neutral | neutral | benign | damaging | tolerated |
| ENST00000373409.8 | rs553499095 | K521Q | deleterious - low confidence | possibly damaging | medium | neutral | neutral | neutral | benign | damaging | tolerated |
| ENST00000373409.8 | rs553499095 | K521E | deleterious - low confidence | benign | medium | Disease | Disease | Disease | benign | damaging | tolerated |
| ENST00000373409.8 | rs1559420819 | K521R | deleterious - low confidence | benign | low | neutral | neutral | neutral | benign | damaging | tolerated |
| ENST00000373409.8 | rs987494598 | K522N | deleterious - low confidence | probably damaging | medium | neutral | Disease | neutral | benign | damaging | tolerated |
| ENST00000373409.8 | rs746578451 | G523R | deleterious - low confidence | possibly damaging | low | neutral | neutral | neutral | benign | damaging | tolerated |
| ENST00000373409.8 | rs1169660767 | G523A | deleterious - low confidence | benign | medium | neutral | neutral | neutral | benign | damaging | tolerated |
| ENST00000373409.8 | rs577014868 | R524Q | tolerated - low confidence | probably damaging | medium | neutral | neutral | neutral | benign | benign | tolerated |
| ENST00000373409.8 | rs769084242 | V525L | tolerated - low confidence | probably damaging | low | neutral | neutral | neutral | benign | benign | tolerated |
| ENST00000373409.8 | rs1700533882 | K526R | tolerated - low confidence | probably damaging | medium | neutral | neutral | neutral | benign | damaging | tolerated |
| ENST00000373409.8 | rs1575871841 | A528P | tolerated - low confidence | benign | low | neutral | Disease | neutral | benign | benign | tolerated |
| ENST00000373409.8 | rs1575871841 | A528S | tolerated - low confidence | benign | neutral | neutral | neutral | neutral | benign | benign | tolerated |
| ENST00000373409.8 | rs1196632178 | K532R | deleterious - low confidence | probably damaging | medium | neutral | Disease | neutral | benign | damaging | tolerated |
| ENST00000373409.8 | rs768200668 | H534Y | deleterious - low confidence | benign | medium | neutral | Disease | neutral | benign | damaging | tolerated |
| ENST00000373409.8 | rs1350310639 | H534P | deleterious - low confidence | possibly damaging | medium | neutral | Disease | neutral | benign | damaging | tolerated |
